# Revision of the *Theopea* genus group (Coleoptera, Chrysomelidae, Galerucinae), part III: Descriptions of two new genera and nine new species

**DOI:** 10.3897/zookeys.912.47719

**Published:** 2020-02-17

**Authors:** Chi-Feng Lee, Jan Bezděk

**Affiliations:** 1 1Applied Zoology Division, Taiwan Agricultural Research Institute, Taichung 413, Taiwan Taiwan Agricultural Research Institute Taichung Taiwan; 2 Mendel University in Brno, Department of Zoology, Zemĕdĕlská 1, 613 00 Brno, Czech Republic Mendel University in Brno Brno Czech Republic

**Keywords:** *
Borneotheopea
*, leaf beetles, *
Pseudotheopea
*, taxonomic revision

## Abstract

This publication treats species within *Theopea* and closely allied genera that were not covered in the previous two revisions. Three species of *Theopea* Baly, 1864 are treated herein, with *T. bicolor* Kimoto, 1989 and *T. mouhoti* Baly, 1864 redescribed, and *T. bicoloroides***sp. nov.** described. A new genus that we consider closely related to *Theopea*, *Pseudotheopea***gen. nov.**, is described. This new genus can be recognized with the presence of reticulate microsculpture on the vertex of the head and pronotum and presence of an apical spine on each metatibia. The following species are transferred to *Pseudotheopea* as new combinations: *Theopea aeneipennis* Gressitt & Kimoto, 1963, *T. azurea* Gressitt & Kimoto, 1963, *T. clypealis* Medvedev, 2015, *T. nigrita* Medvedev, 2007, *T. smaragdina* Gressitt & Kimoto, 1963, *T. similis* Kimoto, 1989, and *T. subviridis* Medvedev, 2012. *Theopea subviridis* Medvedev, 2012 is regarded as **new synonym** of *Pseudotheopea similis* (Kimoto, 1989). In addition, six new species of *Pseudotheopea* are described: *P. boreri***sp. nov.** from India, *P. gressitti***sp. nov.** from Philippines, *P. hsingtzungi***sp. nov.** from Laos, *P. kimotoi***sp. nov.** from Laos, Thailand, and Vietnam, *P. leehsuehae***sp. nov.** from Laos, and *P. sufangae***sp. nov.** from Taiwan. A second new genus regarded as closely related to *Pseudotheopea*, *Borneotheopea***gen. nov.**, can be recognized by possessing uniform antennae in both sexes and lacking an apical spine on each metatibia. Two new species of *Borneotheopea* are described from Borneo: *B. jakli***sp. nov.** and *B. kalimantanensis***sp. nov.**

## Introduction

Species within the genus *Theopea* Baly, 1864 occur in the Oriental Region from north India to Malaysia and Indonesia (Borneo, Sumatra, and Java) and also in the eastern Palaearctic (China) and the Philippines. *Theopea* includes 32 species and two subspecies (Nie et al. 2017). The genus and presumed closely related genera are currently undergoing revision. The first paper (Lee and Bezděk 2018) was devoted to the east Asian species lacking modified clypeus in males and the *T. sauteri* species group. In total, three species were redescribed, five new species described, and two species transferred from *Hoplosaenidea* Laboissière. The second paper (Lee and Bezděk 2019) treated species from Sundaland and the Philippines and redefined the genus. Seventeen species are recognized and classified into four species groups, including seven new species. Eight species were removed from *Theopea* and regarded as species *incertae sedis*.

This research deals with the remaining species that were not treated in the first two papers, including *Theopea aeneipennis* Gressitt & Kimoto, 1963, *T. azurea* Gressitt & Kimoto, 1963, *T. bicolor* Kimoto, 1989, *T. clypealis* Medvedev, 2015, *T. mouhoti* Baly, 1864, *T. nigrita* Medvedev, 2007, *T. smaragdina* Gressitt & Kimoto, 1963, *T. similis* Kimoto, 1989, and *T. subviridis* Medvedev, 2012. In addition, a number of undescribed species are described based on material deposited at various museums. After evaluating the taxonomic status of all species, two new genera, *Pseudotheopea* gen. nov. and *Borneotheopea* gen. nov., are described that conform to modern phylogenetic genus concepts.

## Materials and methods

The abdomens of adults were separated from the bodies and boiled in 10% KOH solution, followed by washing in distilled water to clear and soften genitalia. The genitalia were then dissected from the abdomen, mounted on slides in glycerin, and studied and drawn using a Leica M165 stereomicroscope. For detailed examination a Nikon ECLIPSE 50i microscope was used.

At least two pairs from each species were examined to delimit variability of diagnostic characters. For species collected from more than one locality, at least one pair from each locality was examined. Length was measured from the anterior margin of the eye to the elytral apex, and width at the greatest width of the elytra.

Specimens were available for study and deposited in the following institutions:

**NHMUK**The Natural History Museum, London, UK [Michael Geiser];

**BPBM**Bernice P. Bishop Museum, Hawaii, USA [James Boone];

**CAS**California Academy of Sciences, California, USA [David H. Kavanaugh];

**FREY** The collection of Georg Frey, Naturhistorisches Museum, Basel, Switzerland [Matthias Borer];

**HNHM**Hungarian Natural History Museum, Budapest, Hungary [Ottó Merkl];

**IZAS**Institute of Zoology, Academia Sinica, Beijing, China [Rui-E Nie];

**JBCB** Jan Bezděk collection, Brno, Czech Republic;

**LMCM** Lev N. Medvedev collection, Moscow, Russia;

**MSNG**Museo Civico di Storia Naturale “Giacomo Doria”, Genova, Italy [Roberto Poggi];

**MNHUB**Museum für Naturkunde, Leibniz-Institut für Evolutions- und Biodiversitätsforschung an der Humboldt-Universität zu Berlin, Berlin, Germany [Johannes Frisch];

**NHMB**General collection, Naturhistorisches Museum, Basel, Switzerland [Matthias Borer];

**NMNS**National Museum of Natural Science, Taichung, Taiwan [Jing-Fu Tsai];

**NMPC**National Museum, Praha, Czech Republic [Lukáš Sekerka];

**PAHC** Paul Aston collection, Hong Kong, China;

**RBCN** Ron Beenen collection, Nieuwegein, The Netherlands;

**SEHU**Laboratory for Systematic Entomology, Hokkaido University, Sapporo, Japan [Masahiro Ohara];

**SMNS**Staatliches Museum für Naturkunde Stuttgart, Stuttgart, Germany [Wolfgang Schwaller];

**TARI**Applied Zoology Division, Taiwan Agricultural Research Institute, Taichung, Taiwan [Chi-Feng Lee];

**USNM**Smithsonian Institution, National Museum of Natural History, Washington, U.S.A. [Alexander S. Konstantinov];

**ZSM**Zoologische Staatssammlung München, Munich, Germany [Michael Balke].

Exact label data are cited for all type specimens of previously described species; a double slash (//) divides the data on different labels and a single slash (/) divides the data in different rows. Other comments and remarks are in square brackets: [p] – preceding data are printed, [h] – preceding data are handwritten, [w] – white label, [y] – yellow label, [r] – red label, [y] – yellow label.

## Taxonomy

### Theopea pulchella group

**Remarks.** This species group was defined by Lee and Bezděk (2019). Three species are added to this group.

**Included species.***Theopea bicolor* Kimoto, *T. elegantula* Baly, *T. fairmairei* Duvivier, *T. houjayi* Lee and Bezděk, *T. kedenburgi* Weise, *T. mouhoti* Baly, *T. pulchella* Baly, *T. tsoui* Lee and Bezděk, *T. yuae* Lee and Bezděk, and *T. bicoloroides* sp. nov.

#### Theopea bicolor

Taxon classificationAnimaliaColeopteraChrysomelidae

Kimoto, 1989

804F0C62-E90D-5A52-857B-2D8019323DBC

Figs 1A–C , 2

Theopea bicolor Kimoto, 1989: 199 (Vietnam); Mohamedsaid & Costant, 2007 (Thailand).

##### Type.

Holotype ♂ (BPBM): “VIET NAM. 20 km / N. of Pleiku / 650m. 9.V.1960 [p, w] // L. W. Quate / Collector [p, w] // Theopea / bicolor / n. sp. [h, w] // HOLOTYPE [p, r]”.

##### Other material.

**THAILAND**. Chiang Mai: 3♂♂, 2♀♀ (SEHU), Chiang Dao Valley, 2.V.1980, leg. Y. Komiya; 5♀♀ (SEHU), same locality, 24.V.1983, leg. Y. Komiya; 2♀♀ (SEHU), same but with “leg. H. Akiyama; 3♀♀ (SEHU), same locality, 30.V.1983, leg. Y. Komiya; 1♀ (SEHU), same but with “leg. K. Ikeda”; 1♀ (SEHU), same but with “H. Akiyama”; 4♀♀ (NHMB), same locality, 10-16.V.1991, leg. V. Kubáň; 1♂, 7♀♀ (1♂, 6♀♀: NHMB; 1♀: MSNG), same locality, 17-24.V.1991, leg. V. Kubáň; 4♂♂ (SEHU), Doi Pui, 28.IV.-1.V.1980, leg. Y. Komiya; 1♀ (NMPC), Doi Suthep, 19-22.-IV.1991, leg. S. Bílý; 1♂ (JBCB), Doi Suthep to Doi Pui, 18°49N 99°00E, 19.-23.IV.1991, leg. L. Dembický; 1♂ (MSNG), Palong, 19°55’N 99°06’E, 750 m, 26-28.V.1991, leg. V. Kubáň; Kanchanaburi: 1♂, 4♀♀ (SEHU), Ban Nong Bang, 15.V.1985, leg. Y. Komiya; Mae Hong Son: 6♂♂ (JBCB), Ban Huai Po, 19°19N 97°59E, 1600-2000 m, 9.-16.V.1991, leg. L. Dembický; 1♀ (NHMUK), same locality, 9-16.V.1992, leg. J. Horák; 33♂♂, 2♀♀ (24♂♂, 2♀♀: NHMUK; 9♂♂: JBCB), Ban Si Lang, 1200 m, 1-8.V.1992, leg. J. Horák; 1♀ (JBCB), Kiwlom-pass near Soppong, 19°26N 98°19E, 1400 m, 23.VI.-2.VII.2002, leg. R. and H. Fouqué; 1♀ (JBCB), SE of Soppong, 19°27N 98°20E, 1500 m, 23.-27.V.1999, leg. M. Řiha; 3♂♂, 1♀ (NHMB), Soppong-Pai, 1800 m, leg. Pacholátko; Nan: 1♂ (JBCB), Ban Huay Kon env., 27.V.-10.VI.2002, leg. P. Průdek, M. Obořil; 1♂ (NHMUK), Doi Phuka N.P., V.2000, leg. local collector; 1♀ (SEHU), Mae Kamme Forest, 17.V.1985, leg. Y. Komiya; 1♂ (SEHU), Nan Watershed Res. Station, 17.V.1985, leg. Y. Komiya; 1♂ (SEHU), Wiang Sa, 15.V.1993, leg. S. Ohmomo; 2♀♀ (SEHU), Wieng Ko Sai N.P., 18.V.1985, leg. Y. Komiya; Prachinburi: 1♀ (HNHM), Sakaerat Ecol. Research Institute, 4.VI.2001, leg. E. Harváth and G. Szirákl; VIETNAM. Daklak: 1♂ (MSNG), 12 km SW of Buon Ma Thout, Lake Eakao, 400 m, 26-27.IV.1986, leg. L. Medvedev.

##### Redescription.

**Figure 1.**
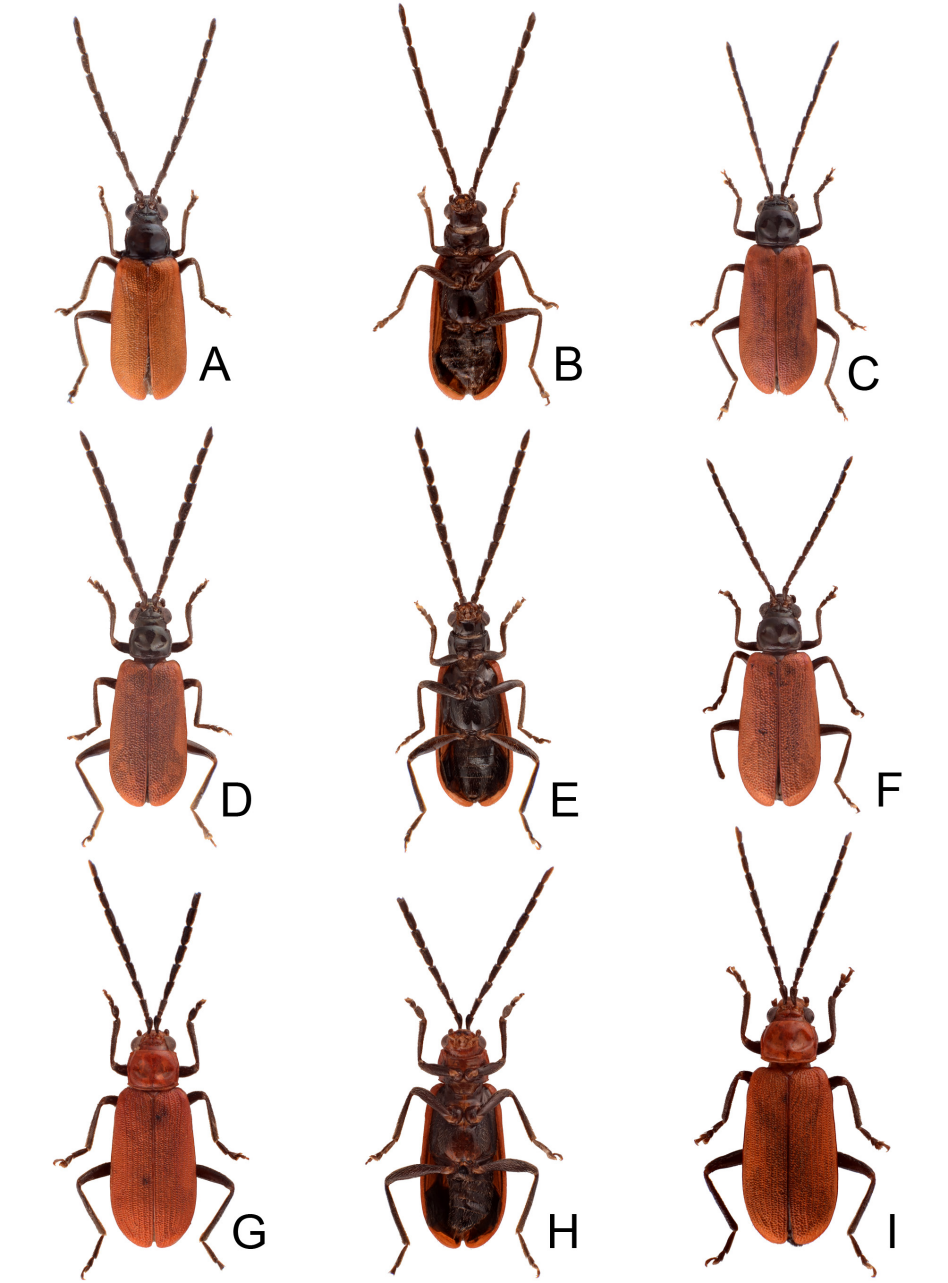
Habitus of *Theopea bicolor*, *T. bicoloroides* sp. nov., and *T. mouhoti*. **A***T. bicolor*, male, dorsal view **B** Same, ventral view **C***T. bicolor*, female, dorsal view **D***T. bicoloroides* sp. nov., male, dorsal view **E** Same, ventral view **F***T. bicoloroides* sp. nov., female, dorsal view **G***T. mouhoti*, male **H** Same, ventral view **I***T. mouhoti*, female, dorsal view.

**Figure 2.**
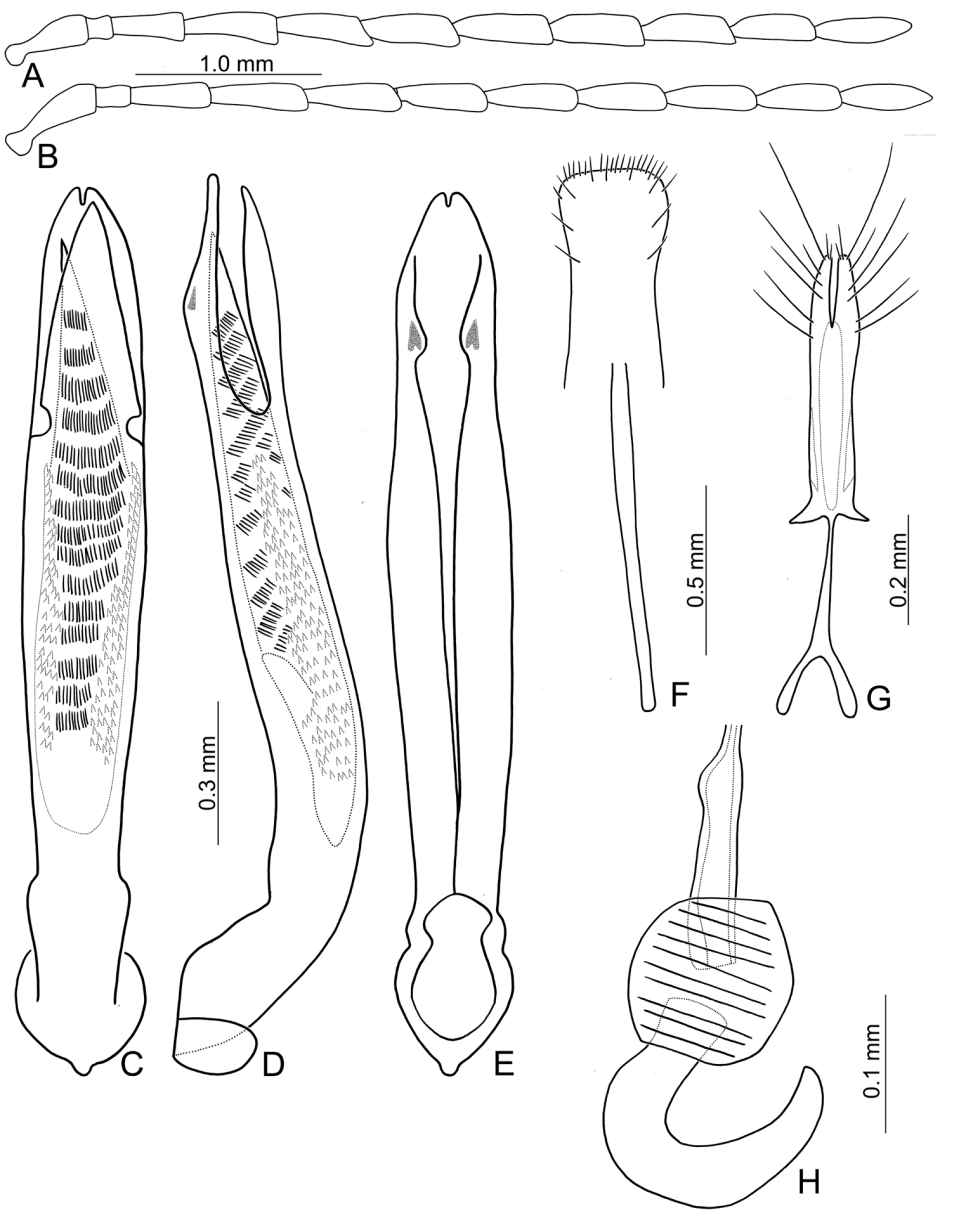
Diagnostic characters of *Theopea bicolor*. **A** Antenna, male **B** Antenna, female **C** Aedeagus, dorsal view **D** Aedeagus, lateral view **E** Aedeagus, ventral view **F** Abdominal ventrite VIII **G** Gonocoxae **H** Spermatheca.

Length 5.8–6.2 mm, width 1.9–2.2 mm. Body color (Fig. 1A–C) dark brown or blackish brown except elytra reddish brown. Antennae filiform in males, but antennomeres VI–VIII slightly swollen (Fig. 2A), length ratios of antennomeres I–XI 1.0: 0.3: 0.8: 1.1: 1.2: 1.2: 1.2: 1.1: 1.1: 1.0: 1.2, length to width ratios of antennomeres I–XI 2.8: 1.2: 2.5: 3.3: 3.6: 3.4: 3.4: 3.2: 3.5: 3.4: 4.1; filiform in females (Fig. 2B), length ratios of antennomeres I–XI 1.0: 0.3: 0.8: 1.0: 1.0: 0.9: 0.9: 0.9: 0.9: 0.8: 0.9, length to width ratios of antennomeres I–XI 3.0: 1.4: 3.1: 3.5: 3.5: 3.2: 3.4: 3.4: 3.6: 3.4: 3.7. Elytra elongate, parallel-sided, 2.0× longer than wide; disc with dense, coarse punctures, arranged into longitudinal rows, with one weak longitudinal ridge between two longitudinal rows of punctures, basally abbreviated. Tarsomeres I of front legs slightly swollen in males; subparallel in females. Aedeagus (Fig. 2C–E) slender, 6.5× longer than wide; sides widest at middle, gradually narrowed towards basal 1/4, gradually and apically narrowed towards apical 1/5, parallel between apical 1/5 and 1/12, apex with shallow notch; tectum well sclerotized, basally broadened, as broad as aedeagus, with hollow area behind base of tectum; moderately curved in lateral view; ventral surface with deep notch from near apex, apically extending into basal opening, more approximate at apical 1/5; triangular sclerites small; internal sac with one median, elongate sclerite, 0.7× as long as aedeagus, apically tapering from basal 1/3, apex acute, connected with short broad sclerite at base, disc with dense transverse rows of hair-like setae and with one pair of elongate, longitudinal rows of stout setae at sides. Gonocoxae (Fig. 2G) elongate, widest at apical 1/6, both gonocoxae joined from basal 1/8 to apical 1/7; apices narrowly rounded, each gonocoxa with eight setae along lateral margin from apex to apical 1/6; with one pair of short lateral processes at basal 2/5. Ventrite VIII (Fig. 2F) elongate and well sclerotized; disc with several long setae at sides and near apical margin, and with dense, short setae along apical margin; spiculum extremely slender. Receptacle of spermatheca (Fig. 2H) strongly swollen; pump slender and strongly curved; proximal spermathecal duct deeply inserted into receptacle, narrow and short.

##### Remarks.

Populations from Laos and southwest China were misidentified. They represent *Theopea bicoloroides* sp. nov. (see below).

##### Diagnosis.

**Figure 3.**
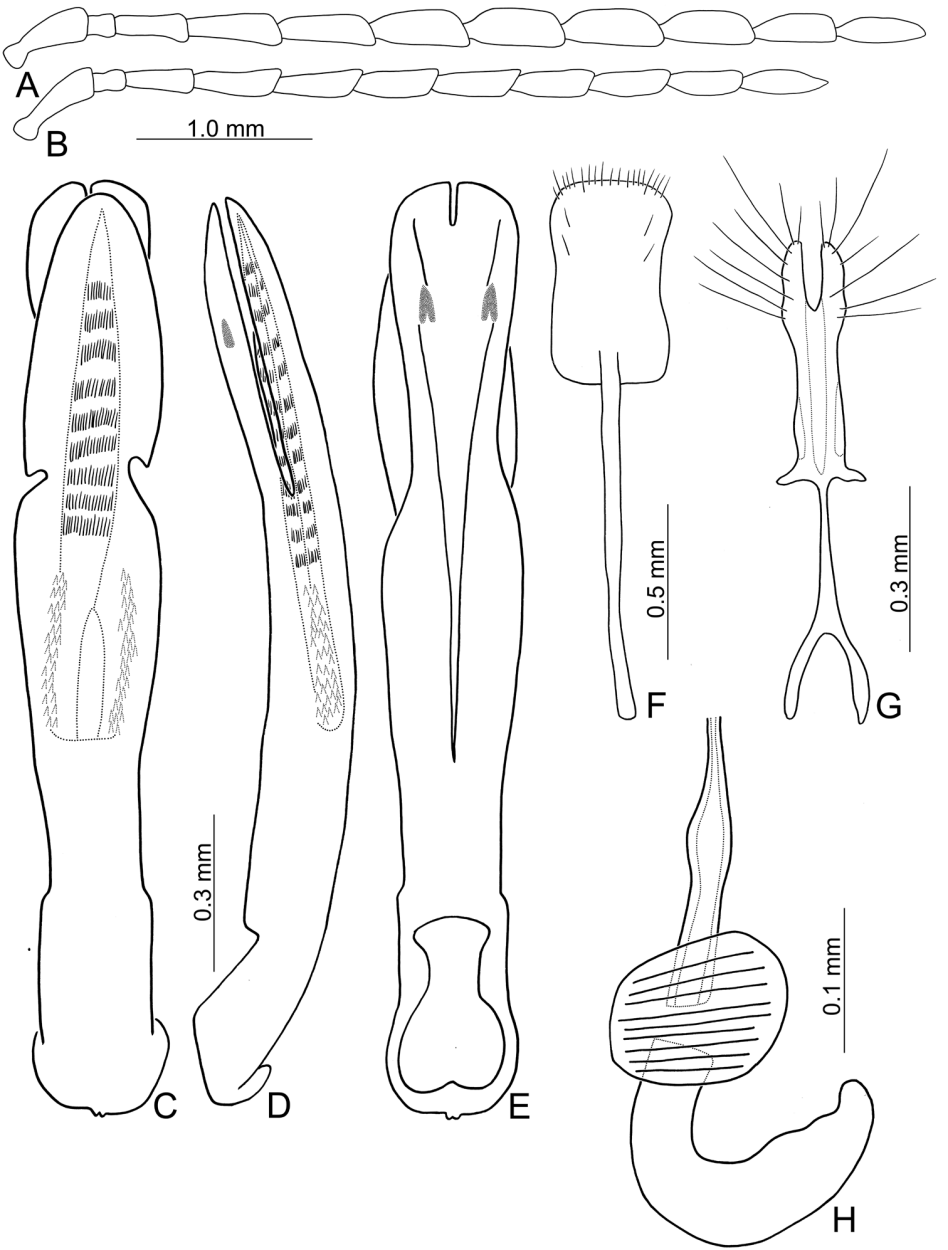
Diagnostic characters of *Theopea bicoloroides* sp. nov. **A** Antenna, male **B** Antenna, female **C** Aedeagus, dorsal view **D** Aedeagus, lateral view **E** Aedeagus, ventral view **F** Abdominal ventrite VIII **G** Gonocoxae **H** Spermatheca.

**Figure 4.**
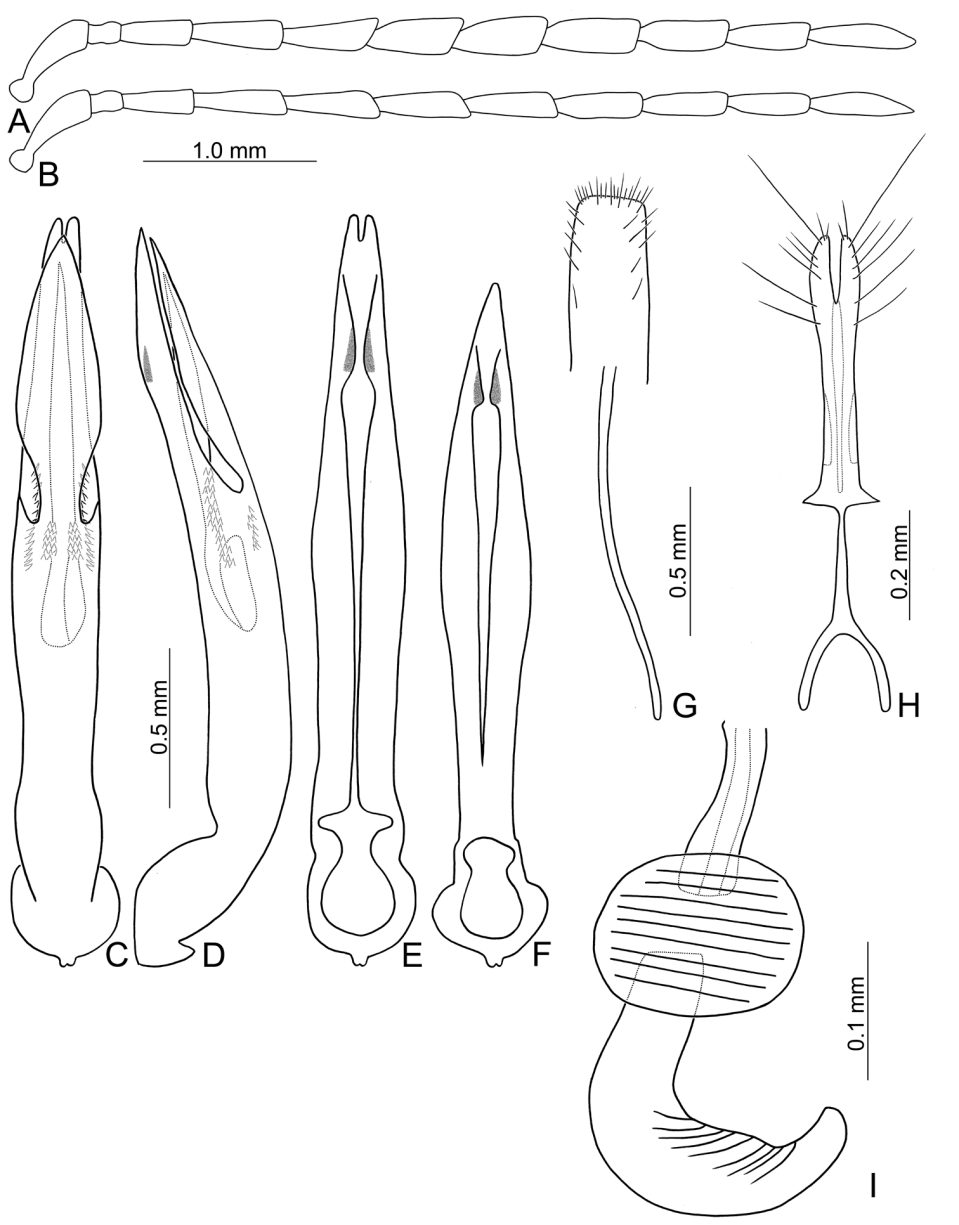
Diagnostic characters of *Theopea mouhoti*. **A** Antenna, male **B** Antenna, female **C** Aedeagus, dorsal view **D** Same, lateral view **E** Same, ventral view **F** Aedeagus, variation, ventral view **G** Abdominal ventrite VIII **H** Gonocoxae **I** Spermatheca.

*Theopea bicolor* Kimoto, *T. bicoloroides* sp. nov., and *T. mouhoti* Baly are characterized by their reddish brown elytra. *Theopea bicolor* and *T. bicoloroides* sp. nov. (Fig. 1A–F) can be easily separately from *T. mouhoti* (Fig. 1G–I) by the dark brown or blackish brown head, prothorax, and scutellum, and indistinct ridges in the elytra. Besides, males of *T. bicolor* and *T. bicoloroides* sp. nov. possess a median elongate sclerite internally in the aedeagus that is covered with transverse rows of hair-like setae (Figs 2C, D; 3C, D). This differs from those of *T. mouhoti*, which lacks such hair-like setae (Fig. 4C, D). *Theopea bicolor* differs from *T. bicoloroides* sp. nov. by the relatively slender antennae (Fig. 2A) in males (length to width ratios of antennomeres V-X more than 3.0 in *T. bicolor*, relatively broader antenna (Fig. 3A), less than 3.0 in *T. bicoloroides* sp. nov.), the narrowly rounded apex of the ventral surface of the aedeagus (Fig. 2E) (broadly rounded apex of aedeagus in *T. bicoloroides* sp. nov. (Fig. 3E)), endophallic sclerite broad and without longitudinal groove in lateral view (Fig. 2D) (dorso-ventrally flattened and with longitudinal groove in lateral view in *T. bicoloroides* sp. nov. (Fig. 3D)), and slender notch at apex of gonocoxae (Fig. 2G) (broad notch at apex of gonocoxae in *T. bicoloroides* sp. nov. (Fig. 3G)).

##### Distribution.

Thailand, Vietnam.

#### Theopea bicoloroides sp. nov.

Taxon classificationAnimaliaColeopteraChrysomelidae

AF809A3E-C48C-52F3-85E7-DBDD43BA9D96

http://zoobank.org/93660DB1-59A6-4C78-A338-A546A6CA2717

Figs 1D–F , 3

Theopea bicolor Kimoto, 1989: 199 (part); Medvedev, 2000: 178 (Laos); Bezděk, 2012: 401 (China: Yunnan) .

##### Types.

Holotype ♂ (NMPC), **LOAS**. 20 km NW Louang Namhta, 21°09.2’N 101°18.7’E, 800-1100 m, 5-11.V.1987, leg. M. Štrba and R. Hergovits; Paratypes. 1♂ (NMPC), same data as holotype; LAOS. Boli Kham Xai: 1♀ (RBCN), Ban Nok env., 18°08.7’N 104°28.1’E, Route no 8, 220 m, 9-14.V.1998, leg. E. Jendek, and O. Sausa; 1♂ (HNHM), Phou Khao Kouay NBCA, Tad Leuk Waterfall, 280 m, 11-12.IV.1998, leg. O. Merkl and G. Csorba (identified as *Theopea bicolor* by Medvedev (2000)); Hua Phan: 3♂♂, 6♀♀ (JBCB), 25km SE Vieng Xai (by road), Ban Kangpabong env., 20°19’N 104°25E, 14.-18.V.2001, leg. J. Bezděk; **CHINA**. Yunnan: 2♀♀ (TARI), Mohan (磨憨), 14.V.2016, leg. Y.-T. Wang.

*Theopea bicolor*: one paratype ♂ (ZSM), labeled: “Laos 1963 / Umgeb. Vanky [p, w] // Theopea / bicolor / n. sp. [h, w] // PARATYPE [p, b]”.

##### Description.

Length 6.1–6.5 mm, width 2.3–2.5 mm. Body color (Fig. 1D–F) dark brown or blackish brown except elytra reddish brown. Antennae filiform in males, but antennomere VI–IX strongly swollen (Fig. 3A), length ratios of antennomeres I–XI 1.0: 0.3: 0.8: 1.0: 1.0: 1.1: 1.1: 1.1: 1.0: 0.9: 1.0, length to width ratios of antennomeres I–XI 2.8: 1.3: 2.6: 3.2: 3.2: 2.7: 2.7: 2.7: 2.7: 2.8: 3.4; more slender in females (Fig. 3B), length ratios of antennomeres I–XI 1.0: 0.3: 0.8: 0.9: 1.0: 0.9: 0.9: 0.9: 0.9: 0.8: 1.0, length to width ratios of antennomeres I–XI 3.0: 1.5: 2.9: 3.3: 3.1: 3.0: 3.3: 3.2: 3.4: 3.1: 3.5. Elytra elongate, parallel-sided, 1.9× longer than wide; disc with dense, coarse punctures arranged into longitudinal rows, with one weak longitudinal ridge between two longitudinal rows of punctures, basally abbreviated. Tarsomeres I of front legs slightly swollen in males; subparallel in females. Aedeagus (Fig. 3C–E) slender, 6.4× longer than wide; sides strongly narrowed at apical 1/4 in ventral view, apical margin truncate, with shallow notch; tectum well sclerotized, basally broadened, broader than aedeagus, with hollow area at base of tectum; slightly curved in lateral view; ventral surface with deep notch from near apex, apically extending into basal 2/5; triangular sclerites small; internal sac with one median, elongate sclerite, 0.6× as long as aedeagus, dorso-ventrally flattened, apically tapering from basal 1/3, apex acute, connected by short broad sclerite at base, disc with dense, transverse rows of hair-like setae and with one pair of elongate, longitudinal rows of stout setae at sides. Gonocoxae (Fig. 3G) elongate, widest at apical 1/6, gonocoxae combined from basal 1/8 to apical 1/7; apices narrowly rounded, each gonocoxa with eight setae along lateral margin from apex to apical 1/6; with one pair of short lateral processes at basal 2/5. Ventrite VIII (Fig. 3F) elongate and well sclerotized; disc with several long setae at sides and near apical margin, and with dense, short setae along apical margin; spiculum extremely slender. Receptacle of spermatheca (Fig. 3H) strongly swollen; pump slender and strongly curved; proximal spermathecal duct deeply inserted into receptacle, narrow and long.

##### Diagnosis.

*Theopea bicolor* Kimoto, *T. bicoloroides* sp. nov., and *T. mouhoti* Baly are characterized by their reddish brown elytra. *Theopea bicolor* and *T. bicoloroides* sp. nov. (Fig. 1A–F) can be easily separately from *T. mouhoti* by the dark brown or blackish brown head, prothorax, and scutellum, and indistinct ridges on the elytra (Fig. 1G–I). In addition, males of *T. bicolor* and *T. bicoloroides* sp. nov. possess median elongate internal aedeagal sclerites that are covered with transverse rows of hair-like setae (Figs 2C, D; 3C, D). This differs from those of *T. mouhoti* that lack hair-like setae (Fig. 4C, D). *Theopea bicoloroides* sp. nov. differs from *T. bicolor* by the relatively broader antennae in males (Fig. 3A) (length to width ratios of antennomeres V-X less than 3.0× in *T. bicoloroides* sp. nov. but more than 3.0 in *T. bicolor* (Fig. 2A)), broadly rounded apex of the ventral surface of the aedeagus (Fig. 3E) (narrowly rounded apex of ventral surface of aedeagus in *T. bicolor* (Fig. 2E)), endophallic sclerite dorso-ventrally flattened and with longitudinal groove in lateral view (Fig. 3D) (broad and lacking longitudinal groove in lateral view in *T. bicolor* (Fig. 2D)); and a broad notch at the apex of the gonocoxae (Fig. 3G) (slender notch at apex of gonocoxae in *T. bicolor* (Fig. 2G)).

##### Etymology.

This new species is named for the similarity with *Theopea bicolor* Kimoto.

##### Distribution.

China: Yunnan; Laos.

#### Theopea mouhoti

Taxon classificationAnimaliaColeopteraChrysomelidae

Baly, 1864

177CACE4-373A-5708-864B-F61B0A5A9451

Figs 1G–I , 4

Theopea mouhoti Baly, 1864: 238 (Thailand); Wilcox, 1973: 631 (catalogue); Kimoto, 1989: 200 (Laos); Staines & Staines, 1999: 522 (catalogue).

##### Types.

Holotype ♂ (NHMUK, by monotypy), labeled: “Type [p, w, circle label with red border] // Theopea / Mouhoti / Baly / Siam [h, g] // Baly Coll. [p, w]”.

##### Other specimens examined.

**CAMBODIA**. 1♂ (NHMUK), Chautd; **LAOS**. Attapu: 5♂♂, 2♀♀ (NHMUK), Bolaven Plateau, 15 km SE of Ban Huangkong, Nong Lom (Lake) env., 15°02’N 106°35’E, 800 m, 18-30.IV.1999, leg. E. Jendek and O. Šauša; Boli Kham Xai: 1♂, 3♀♀ (RBCN), Ban Nok env., 18°08.7’N 104°28.1’E, Route no 8, 220 m, 9-14.V.1998, leg. E. Jendek, and O. Šauša; Champasak: 4♀♀ (NHMB), Ban Nong Panouan env., 15°02’N 106°31-34’E, 770-800 m, leg. M. Geiser and D. Hauck; Khammouane: 2♂♂, 8♀♀ (NHMB), Ban Khoun Ngeun, 18°07’N 104°29’E, 200 m, 24-29.IV.2001, leg. Pacholátko; Vientiane: 1♂ (ZSM), III.-VI.1963 (identified by Kimoto (1989); **THAILAND**. 4♂♂ (NHMUK); Loei: 1♀ (RBCN), Phu Rua N.P., 17°30’N 101°21’E, 6-9.IV.1999, leg. M. Říha.

##### Redescription.

Length 6.5–8.0 mm, width 2.4–3.1 mm. Body color (Fig. 1G–I) reddish brown; meso- and metathoracic ventrites, abdomen, and legs dark brown or blackish brown; antenna black but antennomere XI reddish brown. Antennae filiform in males, but antennomere VI–VIII moderately swollen (Fig. 4A), length ratios of antennomeres I–XI 1.0: 0.3: 0.7: 0.9: 1.0: 1.0: 1.0: 1.0: 0.9: 0.8: 1.1, length to width ratios of antennomeres I–XI 3.3: 1.5: 2.8: 3.1: 3.1: 2.8: 2.6: 2.6: 3.0: 3.0: 4.5; filiform in females (Fig. 4B), length ratios of antennomeres I–XI 1.0: 0.3: 0.7: 0.9: 0.9: 0.9: 0.9: 0.9: 0.8: 0.8: 1.0, length to width ratios of antennomeres I–XI 3.2: 1.5: 2.8: 3.7: 3.6: 3.6: 3.6: 3.5: 3.8: 3.9: 4.6. Elytra elongate, parallel-sided, 1.8-2.0× longer than wide; disc with dense, coarse punctures, arranged into longitudinal rows, with one distinct longitudinal ridge between two longitudinal rows of punctures. Tarsomeres I of front legs swollen in males; subparallel in females. Aedeagus (Fig. 4C–E) slender, 8.4× longer than wide; sides widest at middle, gradually narrowed towards basal 1/4, moderately and apically narrowed, apex with shallow notch; tectum well sclerotized, basally broadened, broader than aedeagus, with hollow area behind base of tectum; moderately curved in lateral view; ventral surface with deep notch from near apex, apically extending into basal opening, more approximate in apical 1/5; triangular sclerites small; internal sac with one median, elongate sclerite, 0.5× as long as aedeagus, apically tapering from basal 1/3, apex acute, connected by short, broad sclerite at base; with one pair of elongate, longitudinal rows of stout setae, and one pair of short, longitudinal rows of stout setae dorsally and basally. Gonocoxae (Fig. 4H) elongate, widest at apical 1/9, both gonocoxae combined from basal 1/7 to apical 1/7; apices narrowly rounded, each gonocoxa with eight setae along lateral margin from apex to apical 1/6; with one pair of short lateral processes at basal 2/5. Ventrite VIII (Fig. 4G) elongate and well sclerotized; disc with several long setae at sides and near apical margin, and with dense, short setae along apical margin; spiculum extremely slender. Receptacle of spermatheca (Fig. 4I) strongly swollen; pump slender and strongly curved; proximal spermathecal duct deeply inserted into receptacle, narrow and short.

##### Variations.

Males of Laos have apically tapering and truncate apex of aedeagus and its ventral surface have median notch not extending into basal opening (Fig. 4F).

##### Diagnosis.

*Theopea mouhoti* Baly, *T. bicolor* Kimoto, and *T. bicoloroides* sp. nov. are characterized by their reddish brown elytra. *Theopea mouhoti* (Fig. 1G–I) can be easily separately from *Theopea bicolor* and *T. bicoloroides* sp. nov. (Fig. 1A–F) by the reddish brown head, prothorax, and scutellum, and distinct and convex ridges on the elytra. Further, males of *T. mouhoti* have median elongate internal aedeagal sclerites without transverse rows of hair-like setae (Fig. 4C, D). This differs from those of *T. bicolor* and *T. bicoloroides* sp. nov., which possess a median elongate internal aedeagal sclerite with hair-like setae in transverse rows (Figs 2C, D; 3C, D).

##### Distribution.

Cambodia, Laos, Thailand.

#### Pseudotheopea

Taxon classificationAnimaliaColeopteraChrysomelidae

Lee & Bezděk gen. nov.

80496AD4-BB02-568C-9EC2-FF5A487E43C9

http://zoobank.org/94C80C3C-5F26-45CC-B532-AC499817D38D

##### Type species.

*Theopea sauteri* Chûjô, 1935a (here designated)

##### Description.

Body length 4.8–7.2 mm.

***Males***. Head. Eyes moderately large. Anterior part of head not modified or modified (strongly excavated and modified in *P. costata* group). Frontal tubercles prominent, narrow, usually produced at inner anterior angle. Penultimate maxillary palpomere not greatly swollen, apical palpomere conical. Vertex with reticulate microsculpture.

Antenna 11-segmented, filiform and slender, some antennomeres apically expanded or curved in males; antennomere II very short, III long, 1.7–3.5× longer than II, 0.6–1.0× as long as I, 2.4–3.3× as long as wide.

Pronotum quadrate or transverse, 1.2–1.3× as wide as long, broadest at middle, with pair of discal depressions. Anterior pronotal border absent. Lateral margins rounded or subparallel. Disc with reticulate microsculpture.

Elytra. Surface almost glabrous (with scattered erect setae on apical part only) except *P. similis* (Kimoto); punctate and striate, usually with longitudinal ridges between two longitudinal rows of punctures, sometimes ridges reduced or absent in part. Epipleura gradually narrowed to apex. Disc with reticulate microsculpture.

Legs. Procoxae globular, prosternal process reduced to thin depressed ridge but apically expanded, procoxal cavities closed. Protarsomere I more or less swollen. Metatibia simple, with apical spine. Length of metatarsomere I nearly equal to following tarsomeres combined. Tarsal claws appendiculate with basal tooth small and rounded. Metatarsomere I simple.

Abdomen. Last ventrite apically trilobate.

Aedeagus always ventrally flattened, apex with shallow notch. Ventral surface with wide groove, with a constriction formed by two small triangular sclerites that are elongate in some species. Internal sac with median elongate sclerite, divided into two parts; sometimes with single or paired hook-like or longitudinal and apically tapering sclerites.

***Females.*** Antenna slender, unmodified. Protarsomere I not modified. Posterior margin of last ventrite regularly rounded, without incisions. Spermatheca with small receptacle and C-shaped pump. Gonocoxae bifurcate basally, apically convergent, apical part usually with eight long setae. Ventrite VIII longitudinal, longer setae laterally, shorter setae along apical margin, spiculum 1.6-3.5× as long as ventrite VIII.

##### Differential diagnosis.

This new genus possesses the following characters shared with *Theopea* Baly: the punctures on the elytra are striate, with ridges between two longitudinal rows of punctures; spaces between longitudinal rows of punctures broader when ridges are reduced or absent. But *Pseudotheopea* gen. nov. differs from *Theopea* by the presence of reticulate microsculpture on the vertex and pronotum (lacking reticulations in *Theopea*), with apical spine of metatibia (absent in *Theopea*), and antennomeres III-X usually longer and curved in males (antennomeres III-X usually swollen or modified in males of *Theopea*). Genitalic characters that distinguish males of *Pseudotheopea* from those of *Theopea* include the relatively longer tectum (> 0.5× as long as aedeagus) and divided median elongate endophallic sclerite in *Pseudotheopea* (relative shorter tectum and < 0.5× as long as aedeagus and the intact median elongate endophallic sclerite in *Theopea*). In females, the gonocoxae are convergent apically in *Pseudotheopea* (divergent in *Theopea*).

##### Remarks.

All *Theopea* species (11 species) from East Asia studied by Lee and Bezděk (2018) and *T. costata* (Allard) (Lee and Bezděk 2019) are transferred to this new genus. Twelve additional species are recognized as members of *Pseudotheopea* gen. nov. including five species transferred from *Theopea* and seven new species. Two species groups are proposed here (Table 1).

##### Etymology.

This new genus is named for its similarity with the genus *Theopea* Baly.

**Table 1.** Definition of species groups and catalogue of *Pseudotheopea* species.

***Pseudotheopea sauteri* species group**	
Frontoclypeus not modified in males; body metallic blue, longitudinal ridges distinct and few setae on the elytra.	
*P. coerulea* (Gressitt & Kimoto, 1963: 679) (Theopea), **comb. nov.**	China
*P. geiseri* (Lee & Bezděk, 2018: 361) (*Theopea*), **comb. nov.**	India
*P. hainanensis* (Lee & Bezděk, 2018: 361) (*Theopea*), **comb. nov.**	China
*P. laosensis* (Lee & Bezděk, 2018: 363) (*Theopea*), **comb. nov.**	China, Laos, Vietnam
*P. sauteri* (Chûjô, 1935a: 169) (*Theopea*), **comb. nov.**	Taiwan
*P. sekerkai* (Lee & Bezděk, 2018: 372) (*Theopea*), **comb. nov.**	Laos
***Pseudotheopea costata* species group**	
Frontoclypeus modified in males, with concavity between eyes, sometimes with erect processes and setae inside concavity.	
*P. aeneipennis* (Gressitt & Kimoto, 1963: 677) (*Theopea*), **comb. nov.**	China
*P. azurea* (Gressitt & Kimoto, 1963: 677) (*Theopea*), **comb. nov.**	China
*P. boreri* **sp. nov.**	India
*P. clypealis* (Medvedev, 2015: 72) (*Theopea*), **comb. nov.**	Vietnam
*P. costata* (Allard, 1889: 111) (*Ozomena*), **comb. nov.**	Philippines
*P. gressitti* Lee and Bezděk, **sp. nov.**	Philippines
*P. hsingtzungi* **sp. nov.**	Laos
*P. kimotoi* **sp. nov.**	Laos, Thailand, Vietnam
*P. leehsuehae* **sp. nov.**	Laos
*P. smaragdina* (Gressitt & Kimoto, 1963: 680) (*Theopea*), **comb. nov.**	China
*P. sufangae* **sp. nov.**	Taiwan
***Pseudotheopea similis* species group**	
Frontoclypeus not modified in males; longitudinal ridges indistinct and with dense setae on the elytra.	
*P. nigrita* (Medvedev, 2007: 11) (*Theopea*), **comb. nov.**	Thailand
*P. similis* (Kimoto, 1989: 201) (*Theopea*), **comb. nov.**	Laos, Vietnam
= *subviridis* Medvedev, 2012: 67 (*Theopea*) **syn. nov.**	
***Pseudotheopea* species current unassigned to any species group**	
*P. aureoviridis* (Chûjô, 1935b: 85) (*Theopea*), **comb. nov.**	Japan
*P. cheni* (Lee & Bezděk, 2018: 340) (*Theopea*), **comb. nov.**	Taiwan
*P. collaris* (Kimoto, 1989: 75) (*Theopea*), **comb. nov.**	Taiwan
*P. irregularis* (Takizawa, 1978: 129) (*Theopea*), **comb. nov.**	Taiwan
*P. kanmiyai* (Kimoto, 1984: 53) (*Hoplosaenidea*), **comb. nov.**	Taiwan

### Pseudotheopea costata group

**Diagnosis.** Frontoclypeus modified in males, with concavity between eyes, sometimes with erect processes and setae within concavity.

**Included species.***Pseudotheopea aeneipennis* (Gressitt & Kimoto), comb. nov., *P. azurea* (Gressitt & Kimoto), comb. nov., *P. boreri* sp. nov., *P. clypealis* (Medvedev), comb. nov., *P. gressitti* sp. nov., *P. hsingtzungi* sp. nov., *P. kimotoi* sp. nov., *P. leehsuehae* sp. nov., *P. smaragdina* (Gressitt & Kimoto), comb. nov., and *P. sufangae* sp. nov.

#### Pseudotheopea aeneipennis

Taxon classificationAnimaliaColeopteraChrysomelidae

(Gressitt & Kimoto, 1963) comb. nov.

67F0F9A3-7A72-560E-B439-B9D2388CD997

**Figure 5.**
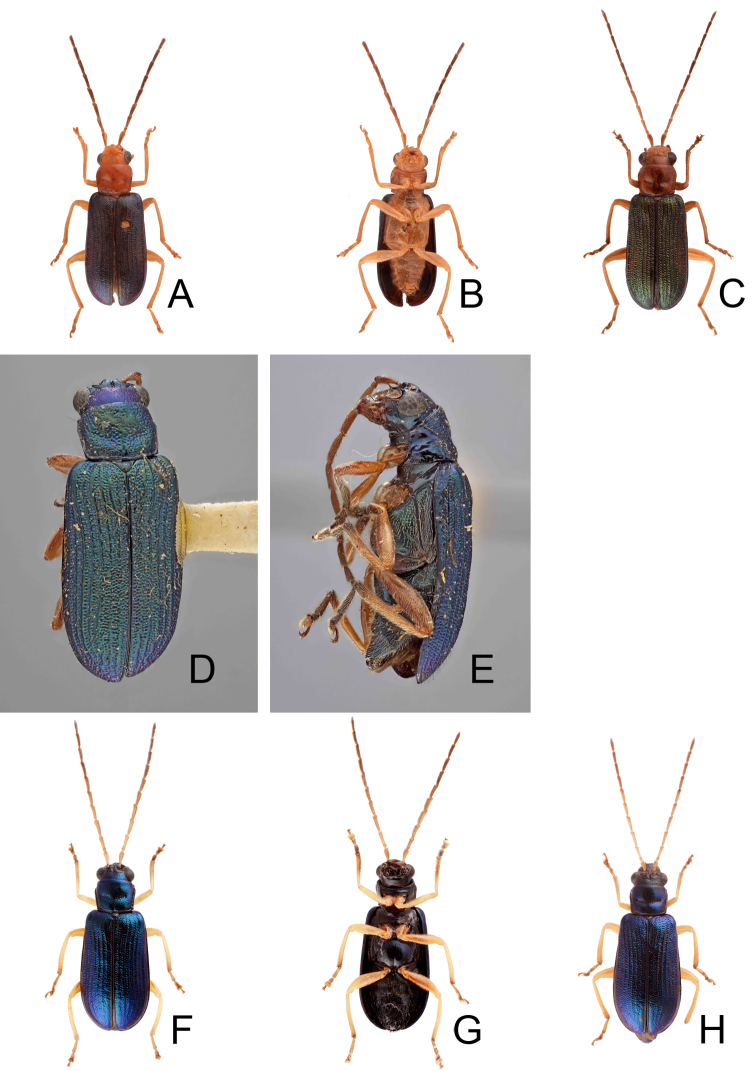
Habitus of *Pseudotheopea aeneipennis*, *P. azurea*, and *P. sufangae* sp. nov. **A***T. aeneipennis*, male, dorsal view **B** Same, ventral view **C***P. aeneipennis*, female, dorsal view **D***P. azurea*, holotype, dorsal view **E** Same, lateral view **F***P. sufangae* sp. nov., male, dorsal view **G** Same, ventral view **H***P. sufangae* sp. nov., female, dorsal view.

**Figure 6.**
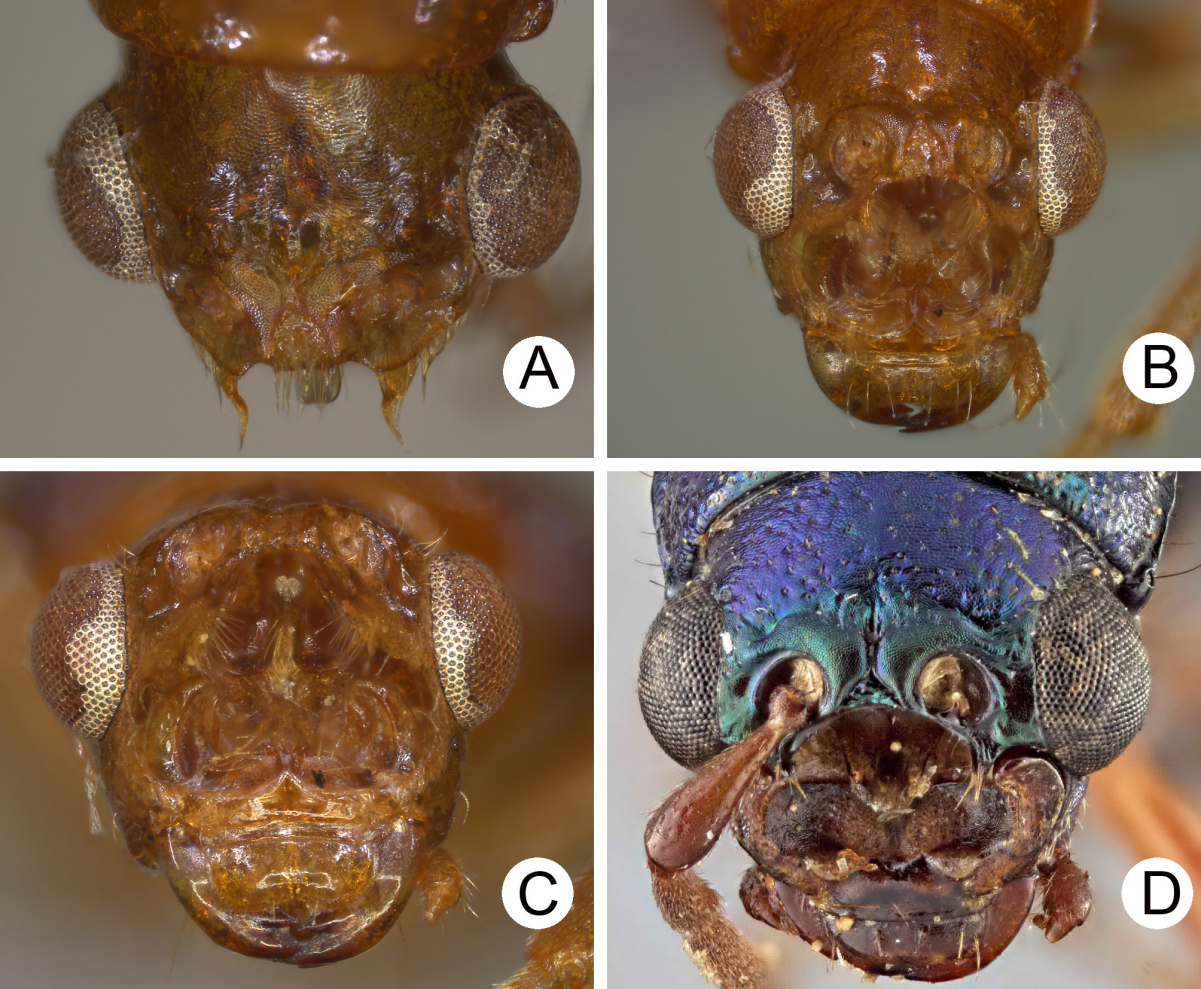
Heads of males of *Pseudotheopea aeneipennis* and *P. azurea*. **A***P. aeneipennis*, dorsal view **B** Same, dorsofrontal view **C** Same, front view **D***P. azurea*, holotype, dorsofrontal view.

**Figure 7.**
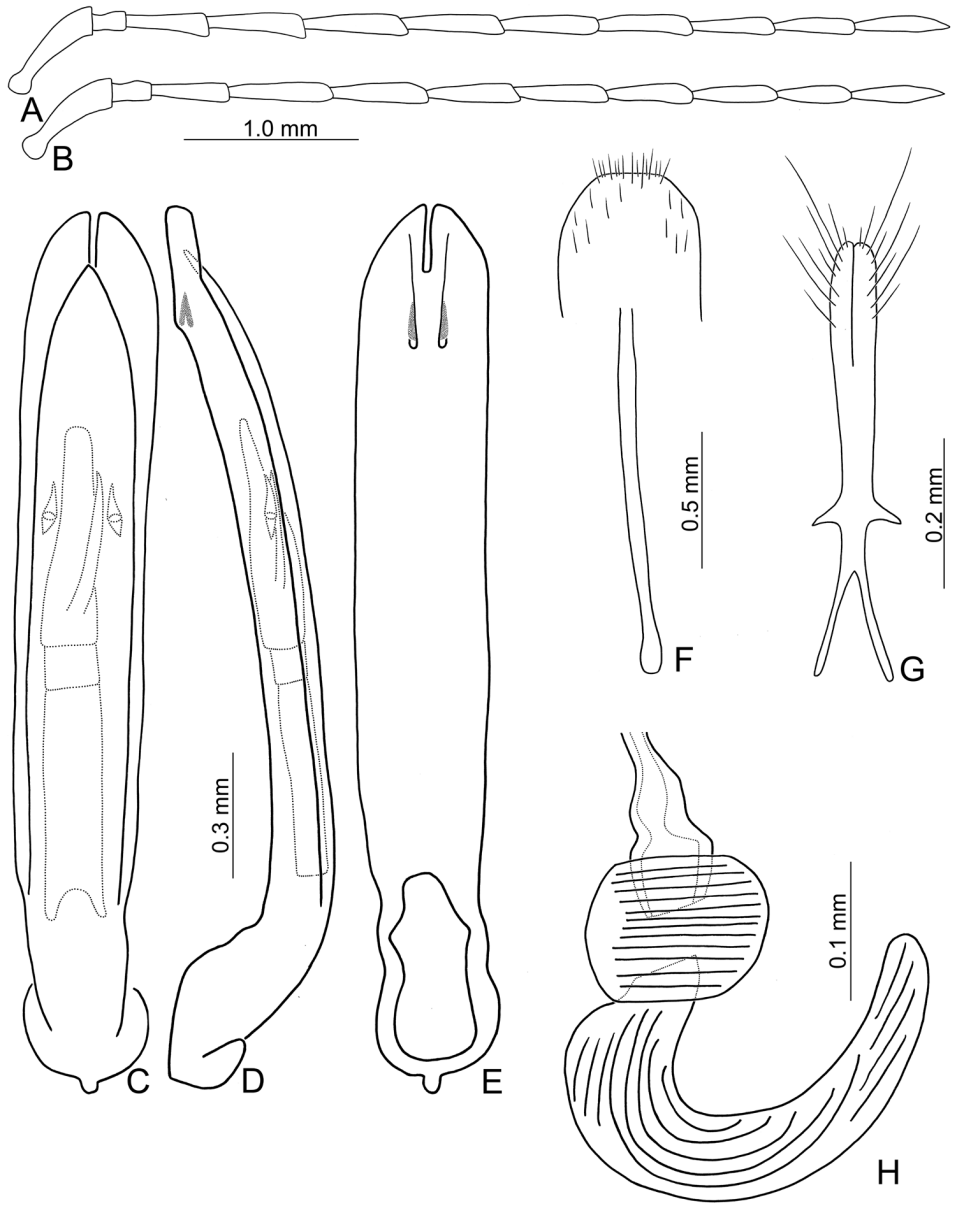
Diagnostic characters of *Pseudotheopea aeneipennis*. **A** Antenna, male **B** Antenna, female **C** Aedeagus, dorsal view **D** Aedeagus, lateral view **E** Aedeagus, ventral view **F** Abdominal ventrite VIII **G** Gonocoxae **H** Spermatheca.

Figs 5A–C , 6A–C , 7

Theopea aeneipennis Gressitt & Kimoto, 1963: 677 (China: Fujian, Jiangxi, Guandong); Wilcox, 1973: 630 (catalogue); Wang et al., 1998: 128 (China: Fujian: Wuyishan); Yang, 2002: 656 (China: Fujian); Yang & Yao, 2002: 447 (China: Hainan Island); Beenen, 2010: 489 (catalogue).

##### Types.

Holotype ♂ (BPBM, by original designation): “Fukian, S. China / Shaowu, TaChuLang / July. 1. 1942 / T. C. Maa [p, w] // HOLOTYPE [P] ♂ / Theopea / aeneipennis [h] / Gressitt and Kimoto [p, r] // Theopea / aeneipennis / holo G and K [h] / J. L. Gressitt det. [p, w]”. Paratypes. 1♀ (CAS): “FUKIEN S. China / Shaowu, Tachulan [p] / 24.VIII.[h]194[p]6[h] T. Maa [p, w] // PARATYPE [p] / Theopea / aeneipennis [h] / Gressitt and Kimoto [p, y] // Theopea / aeneipennis / G and K [h] / Gressitt and Kimoto det. 1961 [p, w]”; 1♀ (BPBM): “FUKIEN S. China / Chungan: Upper / Kuatun 1400 m. / T. C. Maa [p, w] // Aug. 6, 1945 [h, w] // ALLOTYPE [p] / Theopea / aeneipennis [h] / J. L. Gressitt [p, pink label] // Theopea / aeneipennis / ♀ G and K [h] / Gressitt and Kimoto det. 1961 [p, w] // Theopea / sp. nov. 4 / allo. [longitudinal] / aeneipennis [h] / Det. S. Kimoto [p] G and K [h, w]”; 1♀ (NHMUK): “Para- / type [p, w, circle label with yellow border] // FUKIEN, S. China / Chungan, Upper / Kuatun, 1400 m, / T. C. Maa [p, w] // Brit. Mus. / 1963-245. [p, w] // Aug. 6, 1945 [h, w] // PARATYPE [p] / Theopea / aeneipennis [h] / Gressitt and Kimoto [p, y] // aeneipennis [h, w]”; 3♀♀ (CAS): “Tai An Tong, S / Kiangsi pr., S / China VII-6-36 [p, w] // L. Gressitt / Collector [p, w] // PARATYPE [p] / Theopea / aeneipennis [h] / Gressitt and Kimoto [p, y]”; 1♀ (CAS): “CHINA: Kiangsi / Tai-an-hong / VII-4-1936 / J.L. Gressitt [p, w] // L. Gressitt / Collector [p, w] // PARATYPE [p] / Theopea / aeneipennis [h] / Gressitt and Kimoto [p, y]”.

**Other material examined. CHINA**. Fujian: 1♂ (IZAS), Chonganxing Village (崇安星村), Sangan (三港), 740 m, 28.VI.1960, leg. Cai Zuo (左采); 1♂, 1♀ (IZAS), same locality, 740-900 m, 6.VII.1960, leg. Yi-Ran Zhang (張毅然); 1♂ (IZAS), Chonganxing Village (崇安星村), Guadun (掛墩), 840-1210 m, 14.VII.1960, leg. Yi-Ran Zhang (張毅然); 1♀ (IZAS), same locality, 1140 m, 2.VII.1960, leg. Sheng-Qiao Jiang (姜勝巧); 1♀ (IZAS), same locality, 800-1140 m, 22.VII.1960, leg. Fu-Ji Pu (蒲富基); Guangdong: 1♀ (SMNS), Yu-Yueng Nat. Reserve, S Mt. Shi-King-Kong, 24°56’N 112°59’E, 600-1200 m, 28-30.VI.1996, leg. C. Häuser.

##### Redescription.

Length 6.5–6.6 mm, width 2.4-2.5 mm. Body color (Fig. 5A–C) reddish brown or yellowish brown, but antennomeres III-XI more or less darker, elytra greenish bronze. Frontoclypeus (Fig. 6A–C) transverse and deeply excavated between eyes in males, concavity as wide as interspace between eyes; apical margin produced anterior, with clusters of hair-like setae at middle and sides, and convex at sides; with one pair of erect and slender sclerites at center, close to each other, apices rounded; with short, erect, and rounded sclerites insides at middle and sides of basal margin, margined with long hair-like setae and with longitudinal ridges at middle of basal margin. Antennae filiform in males, (Fig. 7A), antennomeres V and VI slightly curved, length ratios of antennomeres I–XI 1.0: 0.3: 0.8: 1.0: 1.0: 1.0: 1.0: 0.9: 0.8: 0.8: 0.9, length to width ratios of antennomeres I–XI 3.7: 1.8: 3.9: 4.4: 5.0: 5.1: 5.3: 5.1: 4.8: 4.6: 5.9; similar in females (Fig. 7B), length ratios of antennomeres I–XI 1.0: 0.4: 0.7: 0.9: 0.9: 0.9: 0.9: 0.8: 0.8: 0.7: 0.9, length to width ratios of antennomeres I–XI 3.7: 1.9: 4.0: 4.7: 4.8: 5.1: 4.9: 4.6: 4,8: 4.2: 5.3. Elytra elongate, parallel-sided, 1.9× longer than wide; disc with dense, coarse punctures, arranged into longitudinal rows, with one indistinct longitudinal ridge between two longitudinal rows of punctures. Tarsomeres I of front legs slightly swollen in males; subparallel in females. Aedeagus (Fig. 7C–E) slender, 7.2× longer than wide; apex with shallow notch; tectum elongate, from apical 1/20 to basal 1/5; dorso-ventrally flattened, slightly curved in lateral view, angular at apical 1/7, straight from apex to apical 1/7; triangular sclerites small; internal sac with elongate, endophallic sclerite complex, 0.5× as long as aedeagus, composed of three sclerites, apical piece as long as basal piece, 0.45× long as entire sclerite; median piece shortest, 0.1× long as entire sclerite; with one elongate, apically sclerite located near base of apical piece; with one pair of short hook-like sclerite at sides. Gonocoxae (Fig. 7G) elongate, both gonocoxae fused from basal 1/4 to apical 1/3; apices convergent and narrowly rounded, each gonocoxa with eight setae along lateral margin from apex to apical 1/6, with lateral processes at basal 2/5. Ventrite VIII (Fig. 7F) elongate and well sclerotized; disc with several long setae at sides and near apical margin, and with dense, short setae along apical margin; spiculum extremely slender. Receptacle of spermatheca (Fig. 7H) tightly joined with pump, pump slender and strongly curved; proximal spermathecal duct deeply inserted into receptacle, narrow and short.

##### Diagnosis.

*Pseudotheopea aeneipennis* (Gressitt and Kimoto) is characterized by its color pattern: reddish brown body with bluish or greenish metallic elytra (Fig. 5A–C). The aedeagus is characterized by its broadly rounded apex, with one additional elongate dorsal sclerite near base of apical piece, and with one pair of small hook-like sclerites at sides near apex of median apical piece (Fig. 7C–E).

##### Distribution.

China (Fujian, Jiangxi, Guandong, Hainan Island).

#### Pseudotheopea azurea

Taxon classificationAnimaliaColeopteraChrysomelidae

(Gressitt & Kimoto, 1963) comb. nov.

3079C31E-08B2-5082-9360-9C124414A141

Figs 5D–E , 6D

Theopea azurea Gressitt & Kimoto, 1963: 677 (China: Guandong); Wilcox, 1973: 630 (catalogue); Wang et al., 1998: 128 (China: Fujian); Yang, 2002: 656 (China: Fujian); Yang & Yao, 2002: 447 (China: Hainan Island); Beenen, 2010: 489 (catalogue).

##### Type.

Holotype ♂ (CAS): “CHINA: Kwang- / tung, Summer / 1950. JL. Gressitt [p, w] // L. Gressitt / Collection [p, w] // HOLOTYPE [p] ♂ / Theopea / azurea [h] / Gressitt and Kimoto [p, r] // Theopea / azurea / holo G and K [h] / J. L. Gressitt det. [p, w] // California Academy / of Sciences / Type / No. [p] 13312 [h, w]”.

**Diagnosis** (based on photographs). Body color (Fig. 5D, E) bluish metallic, mouth parts and antennae dark brown; legs yellowish brown but tibiae and tarsi dark brown. Frontoclypeus (Fig. 6D) transverse and deeply excavated between eyes in males, concavity as wide as interspace between eyes, apico-lateral angles margined with long hair-like setae except along basal margin; with a pair of erect processes at center, almost reaching level of opening.

##### Distribution.

China (Fujian and Guandong).

#### Pseudotheopea boreri sp. nov.

Taxon classificationAnimaliaColeopteraChrysomelidae

97CB2160-0D93-5312-BF00-8112928D35F2

http://zoobank.org/1646E14D-D30C-4144-813C-628D6B014FF8

**Figure 8.**
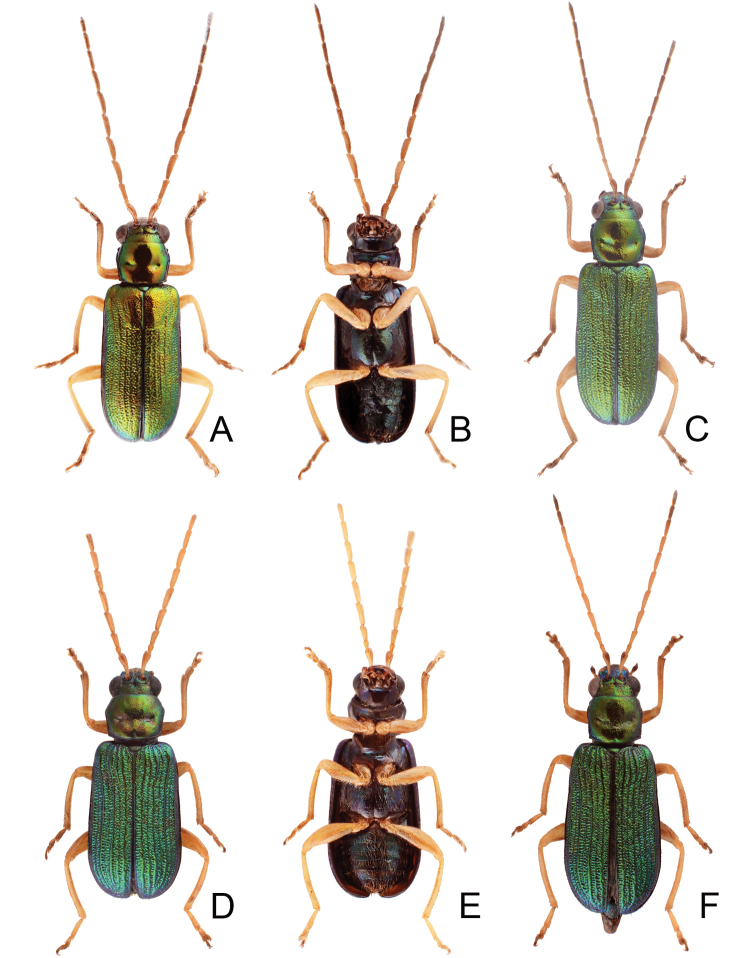
Habitus of *Pseudotheopea boreri* sp. nov. and *P. clypealis*. **A***P. boreri* sp. nov., male, dorsal view **B** Same, ventral view **C***P. boreri*, female, dorsal view **D***P. clypealis*, male, dorsal view **E** Same, ventral view **F***P. clypealis*, female, dorsal view.

**Figure 9.**
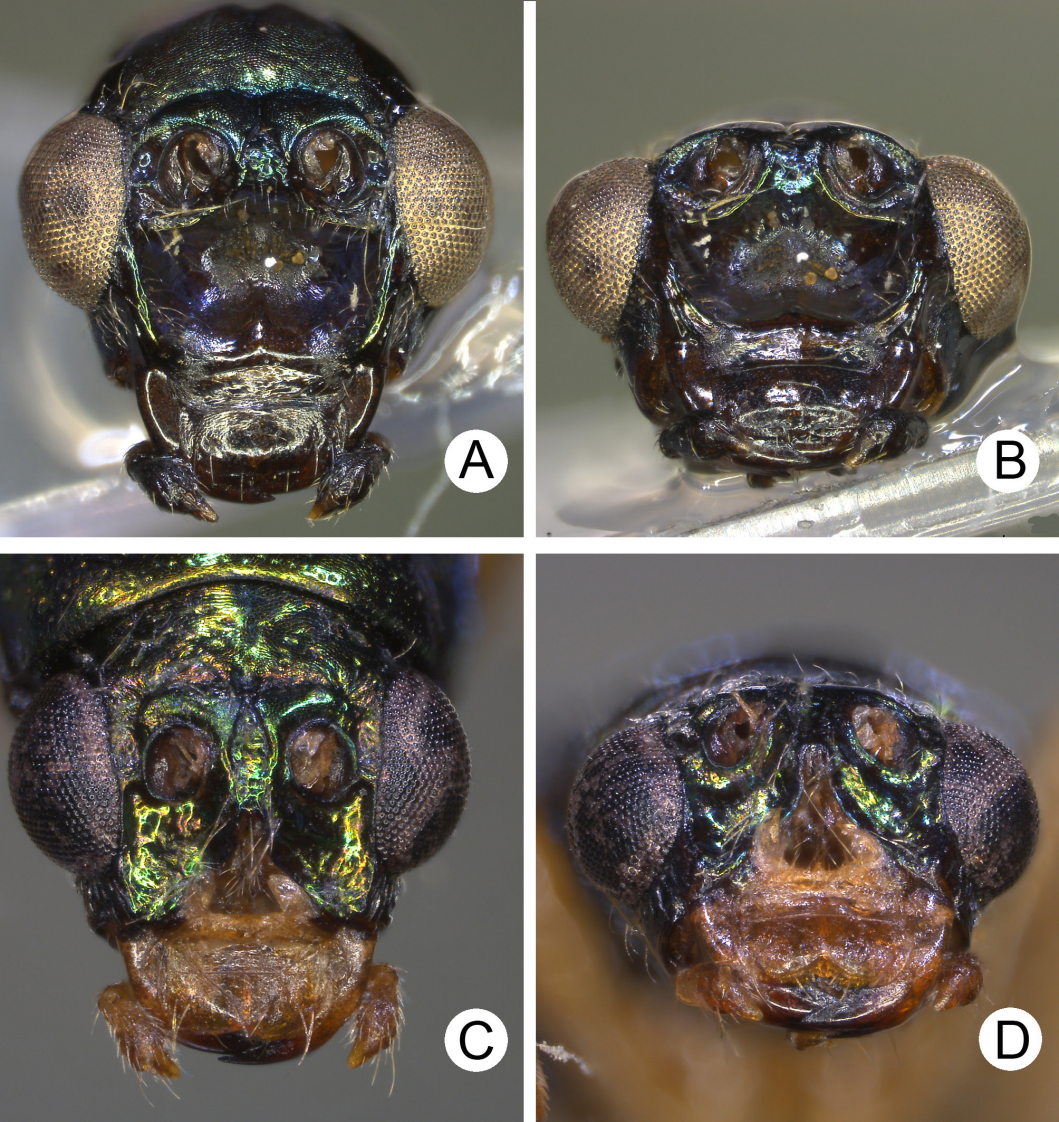
Heads of males of *Pseudotheopea boreri* sp. nov. and *P. clypealis*. **A***P. boreri* sp. nov., dorsofrontal view **B** Same, front view **C***P. clypealis*, dorsofrontal view **D** Same, front view.

**Figure 10.**
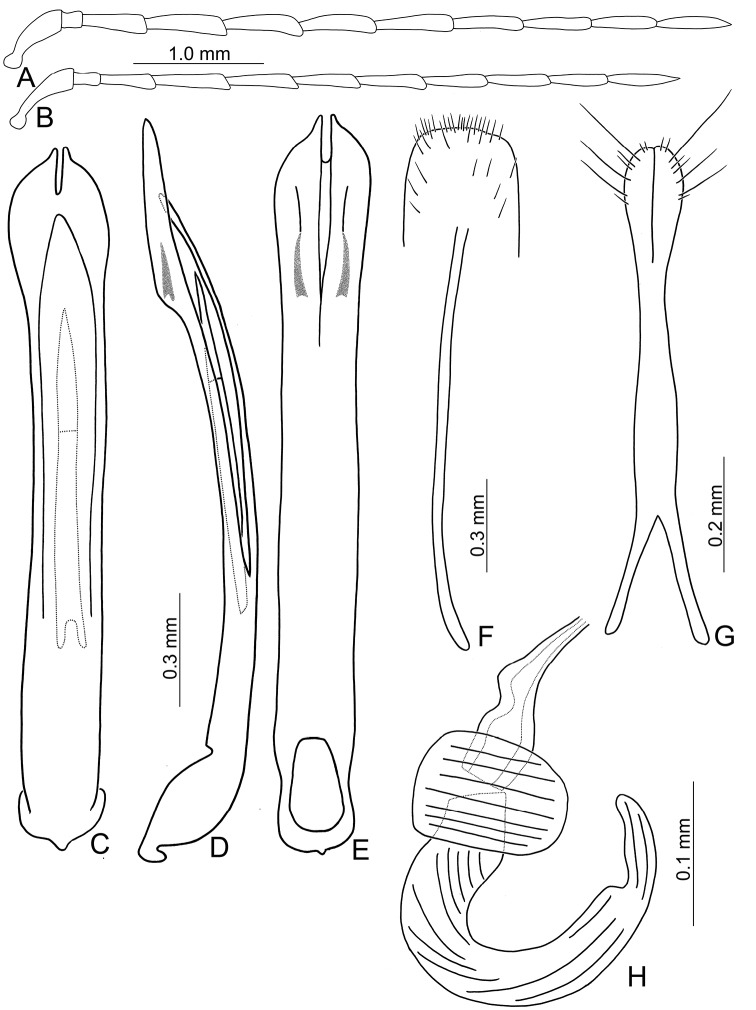
Diagnostic characters of *Pseudotheopea boreri* sp. nov. **A** Antenna, male **B** Antenna, female **C** Aedeagus, dorsal view **D** Aedeagus, lateral view **E** Aedeagus, ventral view **F** Abdominal ventrite VIII **G** Gonocoxae **H** Spermatheca.

Figs 8A–C ; 9A, B ; 10

##### Types.

Holotype ♂ (NHMB), INDIA. Meghalaya, 9 km NW of Jowai, 25°30’N 92°10’E, 1400m, 12.V.1999, leg. Dembický and Pacholátko. Paratypes. 1♂, 2♀♀ (NHMB), same as holotype; **INDIA**. Assam: 1♀ (NMPC), 5 km N of Umrongso, 700 m, 25°27’N 92°43’E, 17.-25.V.1999, leg. J. Rolčík; 1♀ (NHMB), same locality, 21.V.1999, leg. Dembický and Pacholátko”; Meghalaya: 2♀♀ (JBCB), Nokrek N.P., 3km S Daribokgiri, 1400 m, 25°27’N 90°19’E, 26.IV.1999, leg. Rolčík; 1♀ (JBCB), 8 km N of Shillong, 1200 m, 25°38’N 91°54’E, 7.-9.V.2004, leg. R. Businský; 1♂, 1♀ (NHMB), same but with “leg. L. Dembický”.

##### Description.

Length 5.6–6.6 mm, width 2.0–2.6 mm. Body color (Fig. 8A–C) golden green, but legs yellowish brown but apices of tibiae and tarsi darker; mouth parts and antennae dark brown. Frontoclypeus (Fig. 9A, B) transverse and deeply excavated between eyes in males, concavity as wide as interspace between eyes, margined with long hair-like setae except along basal margin; with dense, long hair-like setae at center. Antennae filiform in males, (Fig. 10A), relatively broader than females, antennomeres V and VI slightly curved, length ratios of antennomeres I–XI 1.0: 0.3: 0.9: 1.2: 1.1: 1.1: 1.1: 1.0: 1.0: 0.9: 1.1, length to width ratios of antennomeres I–XI 3.2: 1.3: 3.2: 4.1: 4.4: 4.5: 4.9: 5.1: 5.2: 5.0: 5.8; filiform in females (Fig. 10B), length ratios of antennomeres I–XI 1.0: 0.3: 0.7: 0.9: 0.9: 0.8: 0.8: 0.8: 0.8: 0.8: 0.9, length to width ratios of antennomeres I–XI 3.8: 2.1: 3.7: 4.8: 4.9: 4.4: 4.7: 5.1: 5.3: 4.9: 6.6. Elytra elongate, parallel-sided, 1.8-2.0× longer than wide; disc with dense, coarse punctures, arranged into longitudinal rows, with one indistinct longitudinal ridge between two longitudinal rows of punctures; with distinct convex area behind scutellum in males. Tarsomeres I of front legs swollen in males; subparallel in females. Aedeagus (Fig. 10C–E) extremely slender, 7.7× longer than wide; apex with shallow notch, both apices equal in length; tectum elongate from apical 1/10 to basal 2/5; moderately curved in lateral view, angular at apical 1/4, straight from apex to apical 1/4; triangular sclerites elongate; internal sac with elongate, endophallic sclerite complex, 0.4× as long as aedeagus, composed of two sclerites, apical piece (0.6×) much shorter than basal piece. Gonocoxae (Fig. 10G) elongate, both gonocoxae fused from basal 1/4 to apical 1/5; apices convergent and narrowly rounded, each gonocoxa with eight setae along lateral margin from apex to apical 1/6, some setae extremely short; lateral processes reduced. Ventrite VIII (Fig. 10F) elongate and well sclerotized; disc with several long setae at sides and near apical margin, and with dense, short setae along apical margin; spiculum extremely slender. Receptacle of spermatheca (Fig. 10H) tightly joined with pump, pump slender and strongly curved; proximal spermathecal duct deeply inserted into receptacle, narrow and short.

##### Diagnosis.

*Pseudotheopea boreri* sp. nov. (Fig. 8A–C), *P. clypealis* (Medvedev) (Fig. 8D–F), *P. hsingtzungi* sp. nov. (Fig. 15A–C), and *P. smaragdina* (Gressitt and Kimoto) (Fig. 15D–F), are characterized by their golden green coloration. They can be identified based on their distribution: *P. boreri* sp. nov. from India, *P. clypealis* from Vietnam, *P. hsingtzungi* sp. nov. from Laos, and *P. smaragdina* from China. *Pseudotheopea boreri* sp. nov. (Fig. 8A) is similar to *P. hsingtzungi* sp. nov. (Fig. 15A) and *P. smaragdina* (Fig. 15D) by sharing the indistinct longitudinal ridges on the elytra (convex and distinct longitudinal ridges on the elytra in *P. clypealis* (Fig. 8D)), but differs by the presence of convex area surrounding scutellum and with reduced longitudinal ridges on the elytra in males (Fig. 8A) (without convex area surrounding scutellum on the elytra in those of others (Figs 8D, 15A, D)) and concavity wide between eyes and without erect processes in males (Fig. 9A, B) (concavity wide between eyes with one erect process in those of *P. smaragdina* (Fig. 16C, D); concavity narrowed between eyes and without erect processes in those of *P. hsingtzungi* sp. nov. (Fig. 16A, B)). In males, the internal aedeagal sac of *P. boreri* sp. nov. lacks additional sclerites except the median elongate sclerite (Fig. 10C, D). This structure is shared with males of *P. gressitti* sp. nov. (Fig. 14C, D), and *P. costata* (Allard), *Pseudotheopea boreri* sp. nov. males differ from both species in possessing a dorso-ventrally flattened aedeagus with a sclerotized ventral surface (Fig. 7C–E) (wide aedeagus with membranous ventral surface in *P. gressitti* sp. nov. (Fig. 14C–E)).

##### Etymology.

This new species is dedicated to Matthias Borer (Curator, NHMB), who encouraged the first author to focus his research on leaf beetles.

##### Distribution.

India.

#### Pseudotheopea clypealis

Taxon classificationAnimaliaColeopteraChrysomelidae

(Medvedev, 2015) comb. nov.

B7F77203-3BB9-57AE-B10D-E4D09DFB7BB6

**Figure 11.**
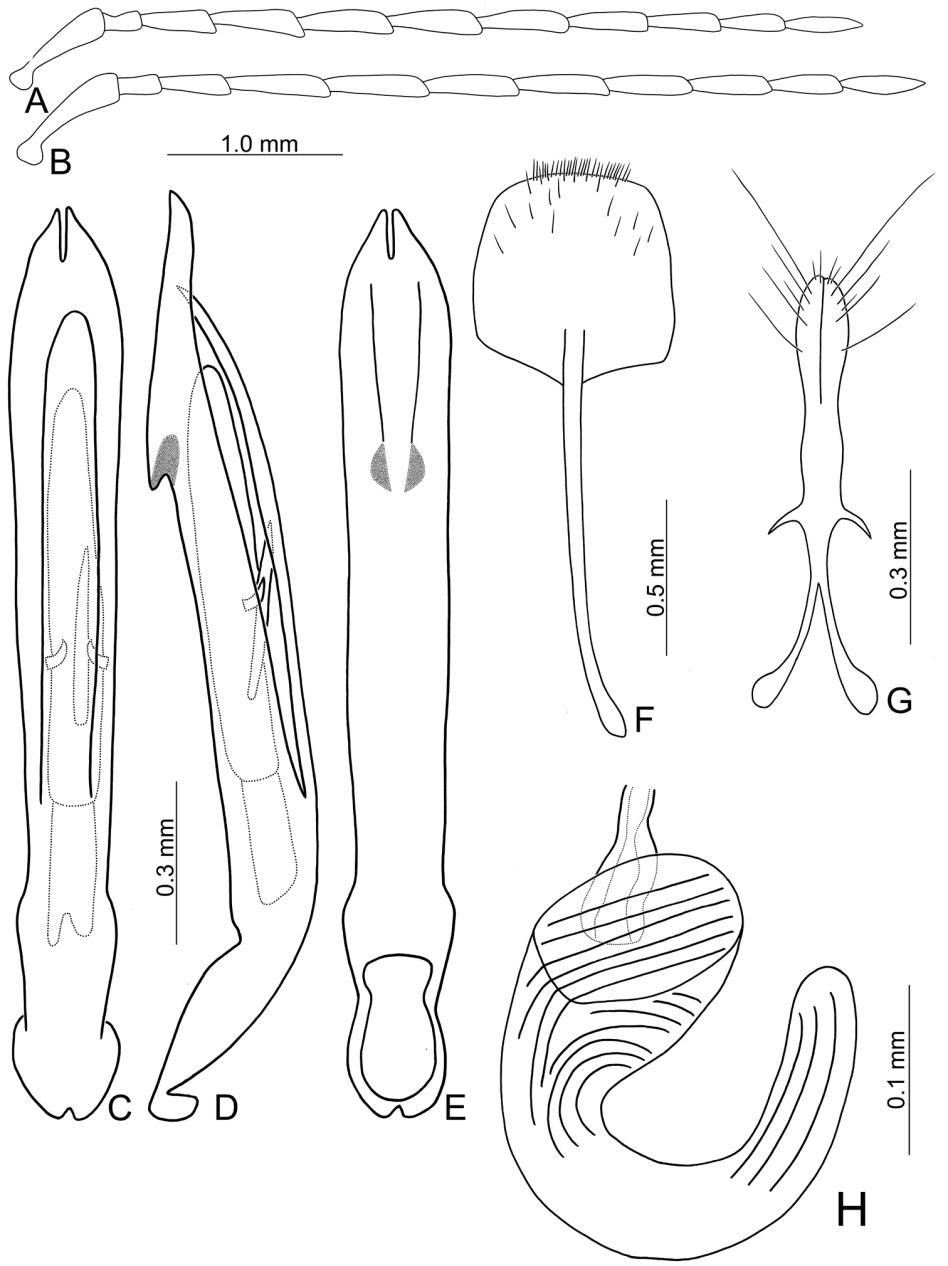
Diagnostic characters of *Pseudotheopea clypealis*. **A** Antenna, male **B** Antenna, female **C** Aedeagus, dorsal view **D** Aedeagus, lateral view **E** Aedeagus, ventral view **F** Abdominal ventrite VIII **G** Gonocoxae **H** Spermatheca.

Figs 8D–F ; 9C, D ; 11

Theopea clypealis Medvedev, 2015: 72 (South Vietnam)

##### Type.

Holotype ♂ (LMCM, based on photographs): “Vietnam Dongnai Pr. / Cat Tien V.99 / leg. A. Polilov [p, w] // HOLOTYPUS / Theopea / clypealis / L. Medvedev [p, r]”.

##### Other specimens examined.

VIETNAM. Kien Giang: 2♂♂, 3♀♀ (TARI), Phu Quoc island, 12-14.IV.2013, leg. Y.-T. Wang; 2♂♂, 1♀ (NMNS), same island, Ding Ba Rd. + Banh Dan Rd., 14.IV.2013, leg. M.-L. Jeng.

##### Redescription.

Length 5.9–6.8 mm, width 2.3–2.6 mm. Body color (Figs 8D–F, 11) golden green, but antennae, mouth parts, and legs yellowish brown, two or three apical antennomeres darker. Frontoclypeus (Fig. 9C, D) transverse and deeply excavated between eyes in males, concavity 0.5× as wide as interspace between eyes, anteriorly narrowed, margined with long hair-like setae and with one erect process at center, margined with hair-like setae; baso-lateral angles covered by rounded membranous sclerites. Antennae filiform in males, (Fig. 11A), antennomeres III-IX slightly curved, length ratios of antennomeres I–XI 1.0: 0.3: 0.7: 0.8: 0.8: 0.8: 0.8: 0.7: 0.7: 0.6: 0.7, length to width ratios of antennomeres I–XI 3.6: 2.0: 3.2: 3.8: 3.8: 3.8: 4.2: 4.3: 3.9: 4.4: 5.3; filiform in females (Fig. 11B), similar to males, length ratios of antennomeres I–XI 1.0: 0.3: 0.6: 0.8: 0.8: 0.7: 0.7: 0.7: 0.6: 0.6: 0.7, length to width ratios of antennomeres I–XI 4.0: 2.0: 3.3: 4.8: 4.9: 4.4: 4.8: 4.7: 4.3: 3.9: 4.5. Elytra elongate, parallel-sided, 1.8× longer than wide; disc with dense, coarse punctures, arranged into longitudinal rows, with one longitudinal ridge between two longitudinal rows of punctures with convex, with distinct and indistinct ridges intertwined. Tarsomeres I of front legs swollen in males; subparallel in females. Aedeagus (Fig. 11C–E) extremely slender, 10.0× longer than wide; apex with shallow notch, both apices equal in length; tectum elongate, from apical 1/10 to basal 2/5; almost straight but moderately curved at basal 1/5 in lateral view, apically curved, angular at apical 1/3; triangular sclerites small; internal sac with elongate, endophallic sclerite complex, 0.6× as long as aedeagus, composed of two sclerites, apical piece (4.0×) much longer than basal piece, one dorsal sclerite slender, 0.3× as long as endophallic sclerite; ventral sclerites absent but one additional pair of hook-like sclerites present. Gonocoxae (Fig. 11G) elongate, both gonocoxae fused from basal 1/3 to apical 1/4; apices convergent and narrowly rounded, each gonocoxa with seven setae along lateral margin from apex to apical 1/6; with one pair of short lateral processes at basal 2/5. Ventrite VIII (Fig. 11F) elongate and well sclerotized; disc with several long setae at sides and near apical margin, and with dense, short setae along apical margin; spiculum extremely slender. Receptacle of spermatheca (Fig. 11H) tightly joined with pump, pump slender and strongly curved; proximal spermathecal duct deeply inserted into receptacle, narrow and short.

##### Diagnosis.

*Pseudotheopea clypealis* (Medvedev) (Fig. 8D–F), *P. boreri* sp. nov. (Fig. 8A–C), *P. hsingtzungi* sp. nov. (Fig. 15A–C), and *P. smaragdina* (Gressitt and Kimoto) (Fig. 15D–F), are characterized by their golden green coloration. They can be identified based on their distribution: *P. boreri* sp. nov. from India, *P. clypealis* from Vietnam, *P. hsingtzungi* sp. nov. from Laos, and *P. smaragdina* from China. *Pseudotheopea clypealis* can be separated from the others by the convex and distinct longitudinal ridges on the elytra (Fig. 8D, F) (indistinct longitudinal ridges on the elytra in others (Figs 8A, C; 15A, B, D, E), and the narrower anterior concavity between eyes (Fig. 9C, D) (broadly rounded anterior margin of concavity between eyes in others (Figs 9A, B; 16A–D). Males of *P. clypealis* are similar to those of *P. smaragdina* in possessing one additional elongate dorsal sclerite and one pair of small lateral hook-like sclerites inside the internal sac (Figs 11C, D; 22D, E). They differ in possessing symmetrical aedeagal apices and a relatively longer apical piece (4.0× longer than basal piece, Fig. 11C, D) (asymmetrical apices and relatively shorter apical piece, as long as basal piece in *P. smaragdina* (Fig. 22D, E)).

##### Distribution.

Vietnam.

#### Pseudotheopea gressitti sp. nov.

Taxon classificationAnimaliaColeopteraChrysomelidae

68C7E17D-DCF6-523C-B922-D0A07F5EC052

http://zoobank.org/B7FF7AC0-9480-4998-8E34-A83824BE7C8D

**Figure 12.**
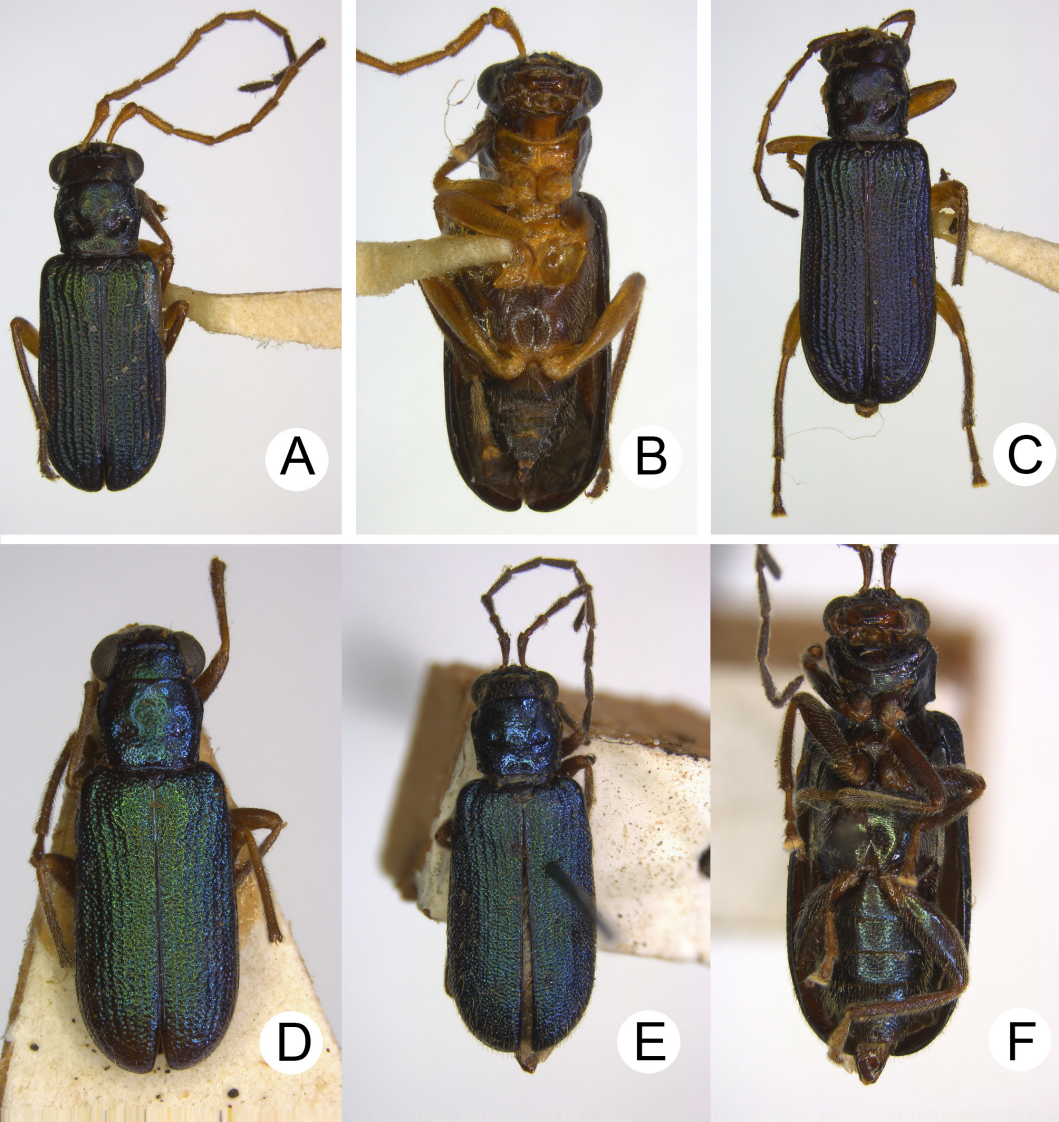
Habitus of *Pseudotheopea gressitti* sp. nov. and *P. similis*. **A***P. gressitti* sp. nov., male, dorsal view **B** Same, ventral view **C***P. gressitti* sp. nov., female, dorsal view **D***P. similis*, male, dorsal view **E***P. similis*, female, dorsal view **F** Same, ventral view.

**Figure 13.**
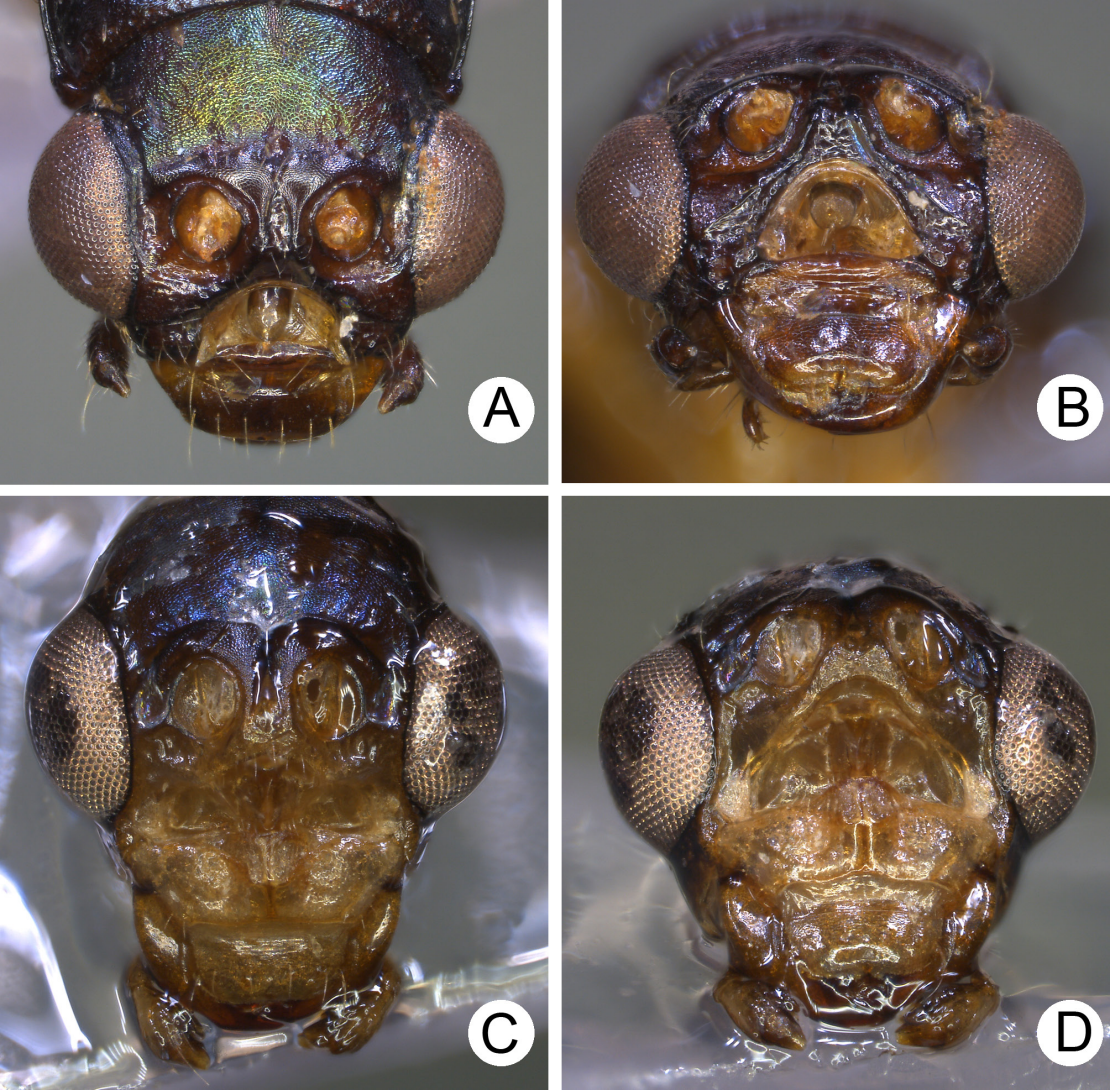
Heads of males of *Pseudotheopea gressitti* sp. nov. and *P. leehsuehae* sp. nov. **A***P. gressitti* sp. nov., dorsofrontal view **B** Same, front view **C***P. leehsuehae* sp. nov., dorsofrontal view **D** Same, front view.

**Figure 14.**
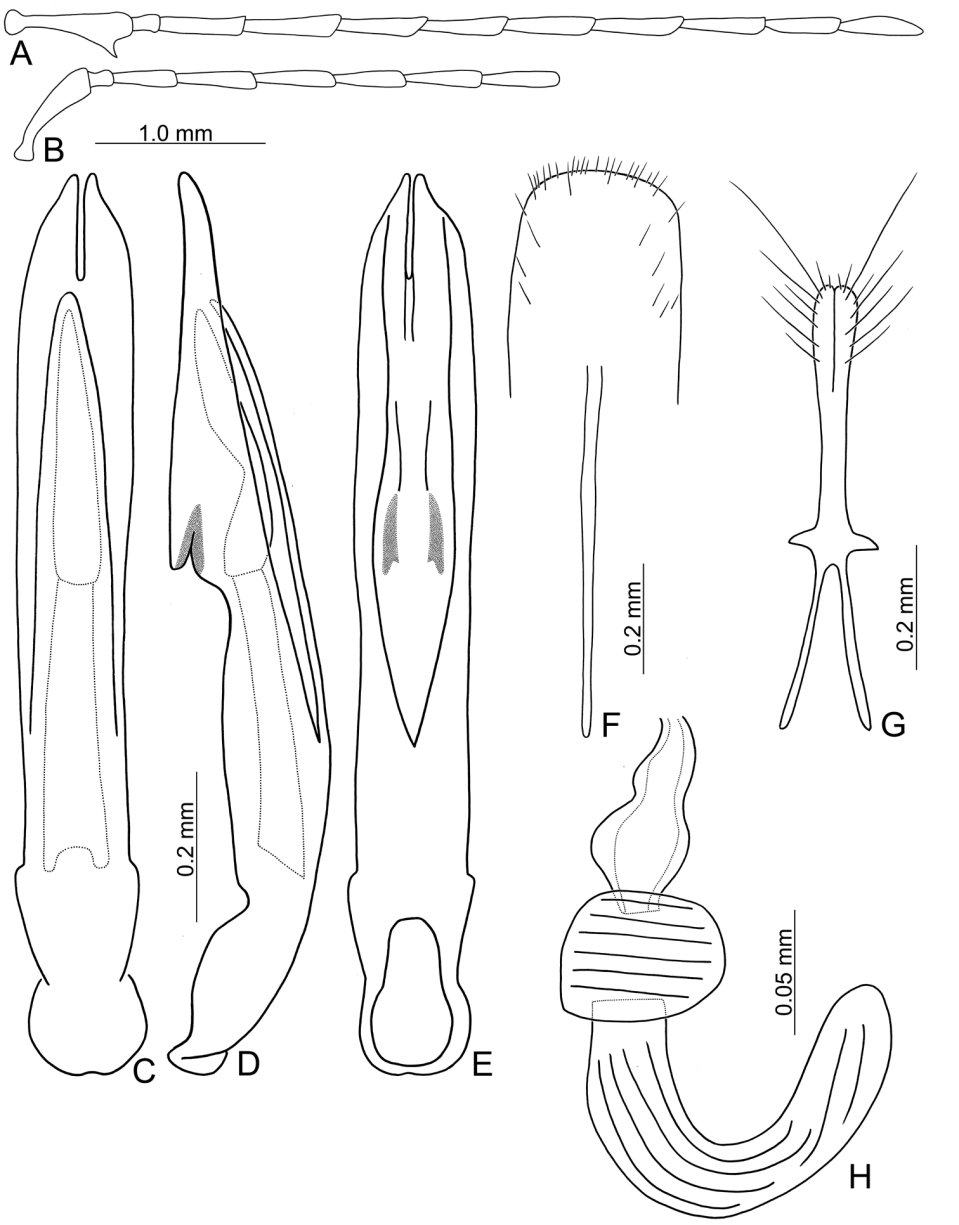
Diagnostic characters of *Pseudotheopea gressitti* sp. nov. **A** Antenna, male **B** Antenna, female **C** Aedeagus, dorsal view **D** Aedeagus, lateral view **E** Aedeagus, ventral view **F** Abdominal ventrite VIII **G** Gonocoxae **H** Spermatheca.

Figs 12A–C ; 13A, B ; 14

##### Types.

Holotype ♂ (USNM), PHILIPPINES, Mindanao: Zamboanga, 1927, leg. Baker. Paratypes. 3♀♀ (USNM), same data as holotype.

##### Description.

Length 5.0-5.7 mm, width 1.8-2.0 mm. Body color (Fig. 12A–C) dark brown; elytra metallic purple, vertex and pronotum with metallic purple reflection, prosternite, mesoventrite, and legs yellowish brown, but tibiae and tarsi darker. Frontoclypeus (Fig. 13A, B) with semi-circular excavation between eyes in males, concavity 0.5× as wide as interspace between eyes, with erect process at center and one pair small processes at baso-lateral angles. Antennae filiform in males (Fig. 14A), with apico-lateral process on antennomere I, length ratios of antennomeres I–XI 1.0: 0.2: 0.7: 0.8: 0.7: 0.7: 0.7: 0.7: 0.7: 0.6: 0.7, length to width ratios of antennomeres I–XI 4.8: 1.6: 4.6: 5.2: 4.9: 4.5: 4.8: 4.9: 4.8: 4.8: 4.5; without apico-lateral process of antennomere I in females (Fig. 14B), relatively shorter than males, length ratios of antennomeres I–VIII (IX-XI lost) 1.0: 0.2: 0.6: 0.7: 0.8: 0.7: 0.7: 0.7, length to width ratios of antennomeres I–XI 4.0: 1.3: 3.7: 4.5: 5.1: 4.7: 4.8: 4.9. Elytra elongate, parallel-sided, 1.9-2.0× longer than wide; disc with dense, coarse punctures, arranged into longitudinal rows, with one distinct longitudinal ridge between two longitudinal rows of punctures. Tarsomeres I of front legs swollen in males; subparallel in females. Aedeagus (Fig. 14C–E) slender, 8.1× longer than wide; apex with shallow notch; tectum elongate, from apical 1/7 to basal 1/3; straight from apex to apical 2/5 in lateral view, angular at apical 2/5; ventral surface membranous from apex to basal 2/5, triangular sclerites small; internal sac with elongate endophallic sclerite, 0.8× as long as aedeagus, composed of two sclerites, apical piece (0.9×) a little shorter than basal piece. Gonocoxae (Fig. 14G) elongate, both gonocoxae fused from basal 1/4 to apical 2/5; apices convergent and narrowly rounded, each gonocoxa with eight setae along lateral margin from apex to apical 1/6, four much longer than others; lateral processes reduced. Ventrite VIII (Fig. 14F) elongate and well sclerotized; disc with several long setae at sides and near apical margin, and with dense, short setae along apical margin; spiculum extremely slender. Receptacle of spermatheca (Fig. 14H) strongly swollen; pump slender and strongly curved; proximal spermathecal duct deeply inserted into receptacle, narrow and short.

##### Diagnosis.

*Pseudotheopea gressitti* sp. nov. is similar to *P. costata* (Allard) in possessing a semi-circular concavity between the eyes in males that includes one erect process at center and one pair of small processes at the baso-lateral angles of the concavity (Fig. 13A, B). However, *P. gressitti* sp. nov. can be recognized by the small body sizes (5.0–5.7 mm long, 7.0–7.2 mm in P. *costata*), metallic purple dorsum (Fig. 12A, C) (reddish brown dorsum in *P. costata*), and a lateral apical process of antennomere I in males (Fig. 14A) (without the lateral process on apex of antennomere I in those of *P. costata*). Males of *P. gressitti* sp. nov. are similar to those of *P. costata* and *P. boreri* sp. nov. based on a median elongate sclerite of the internal sac (Figs 10C, D; 14C, D). Males of *P. gressitti* sp. nov. are similar to those of *P. costata*, based on the broad aedeagi in lateral view (Fig. 14D) (dorso-ventrally flattened in lateral view in those of *P. boreri* sp. nov. (Fig. 10D)). But males differ from those of *P. costata* in possessing smaller triangular sclerites and membranous areas on the ventral surface of the aedeagus extending into basal 1/3 (Fig. 10E) (larger triangular sclerites and membranous areas on the ventral surface of aedeagus only reaching basal 1/2 in *P. costata*).

##### Etymology.

This new species is dedicated to late Dr. J. Linsley Gressitt for his great contribution to the taxonomy of oriental Cerambycidae and Chrysomelidae.

##### Distribution.

Philippines: Mindanao.

#### Pseudotheopea hsingtzungi sp. nov.

Taxon classificationAnimaliaColeopteraChrysomelidae

E14FF08C-E22E-5080-8701-F5640CC643E5

http://zoobank.org/B0707A1E-F90D-45C7-B578-33344BB022D3

**Figure 15.**
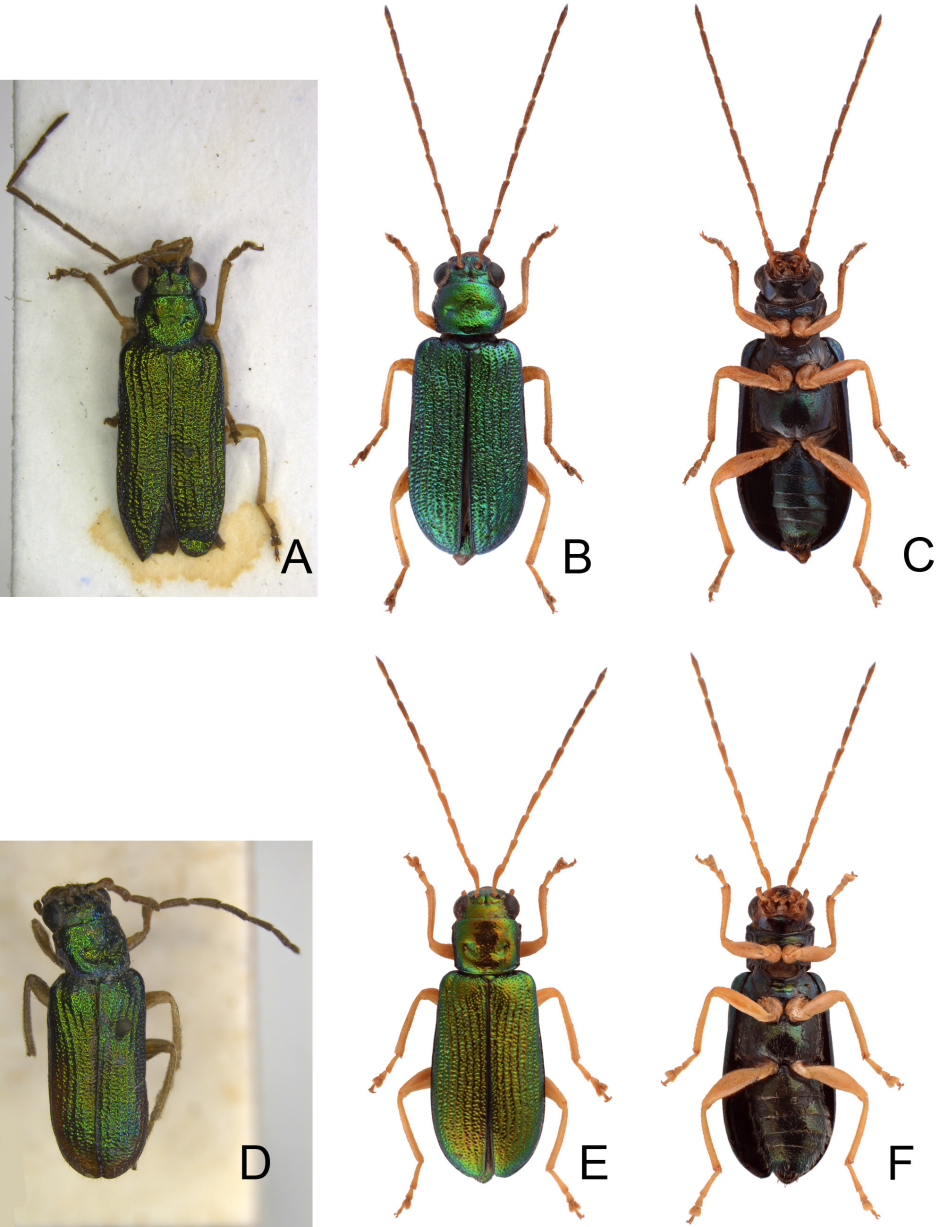
Habitus of *Pseudotheopea hsingtzungi* sp. nov. and *P. smaragdina*. **A***P. hsingtzungi* sp. nov., male, dorsal view **B***P. hsingtzungi* sp. nov., female, dorsal view **C** Same, ventral view **D***P. smaragdina*, male, dorsal view **E***P. smaragdina*, female, dorsal view **F** Same, ventral view.

**Figure 16.**
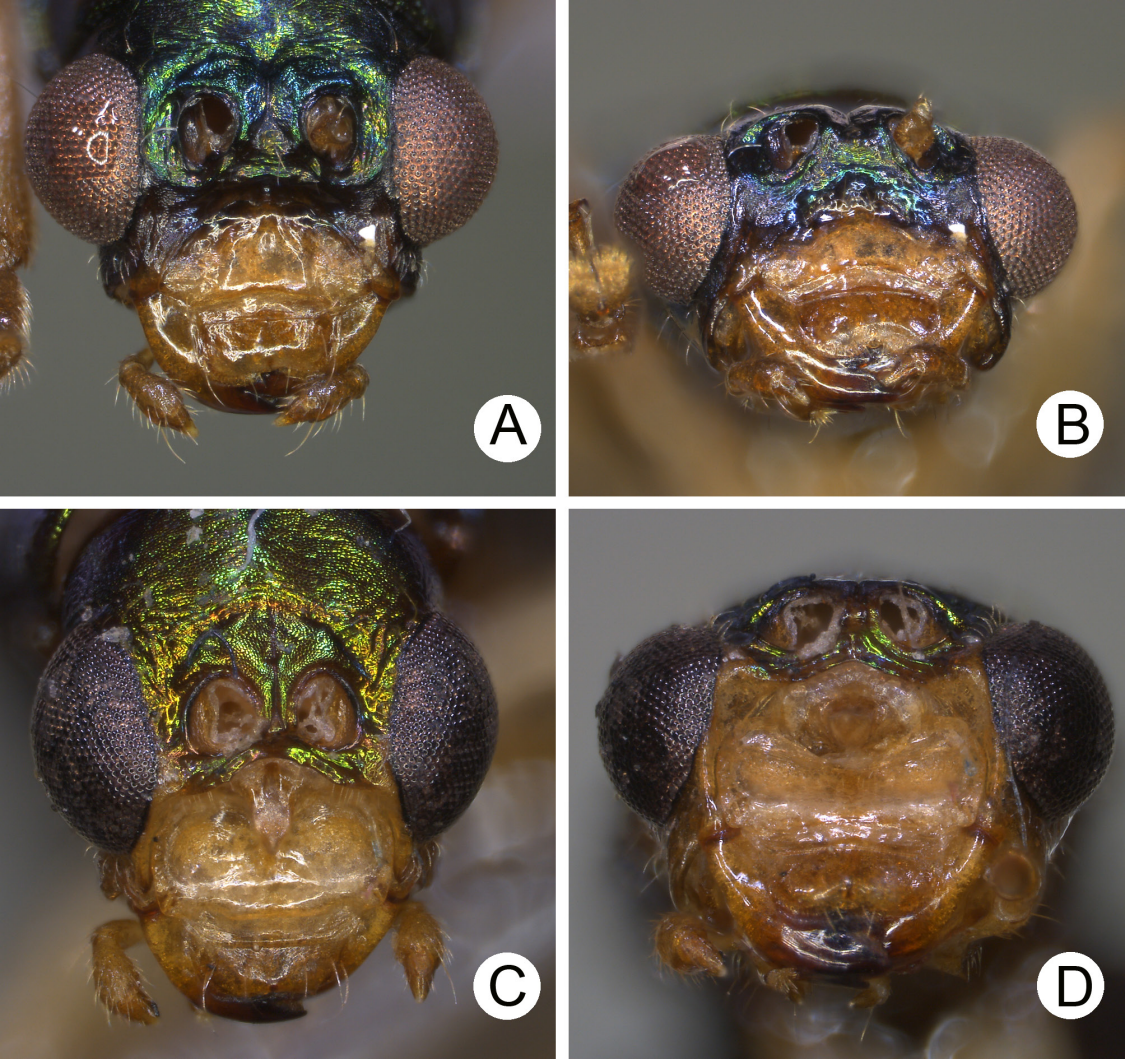
Heads of males of *Pseudotheopea hsingtzungi* sp. nov. and *P. smaragdina*. **A***P. hsingtzungi* sp. nov., dorsofrontal view **B** Same, front view **C***P. smaragdina*, dorsofrontal view **D** Same, front view.

**Figure 17.**
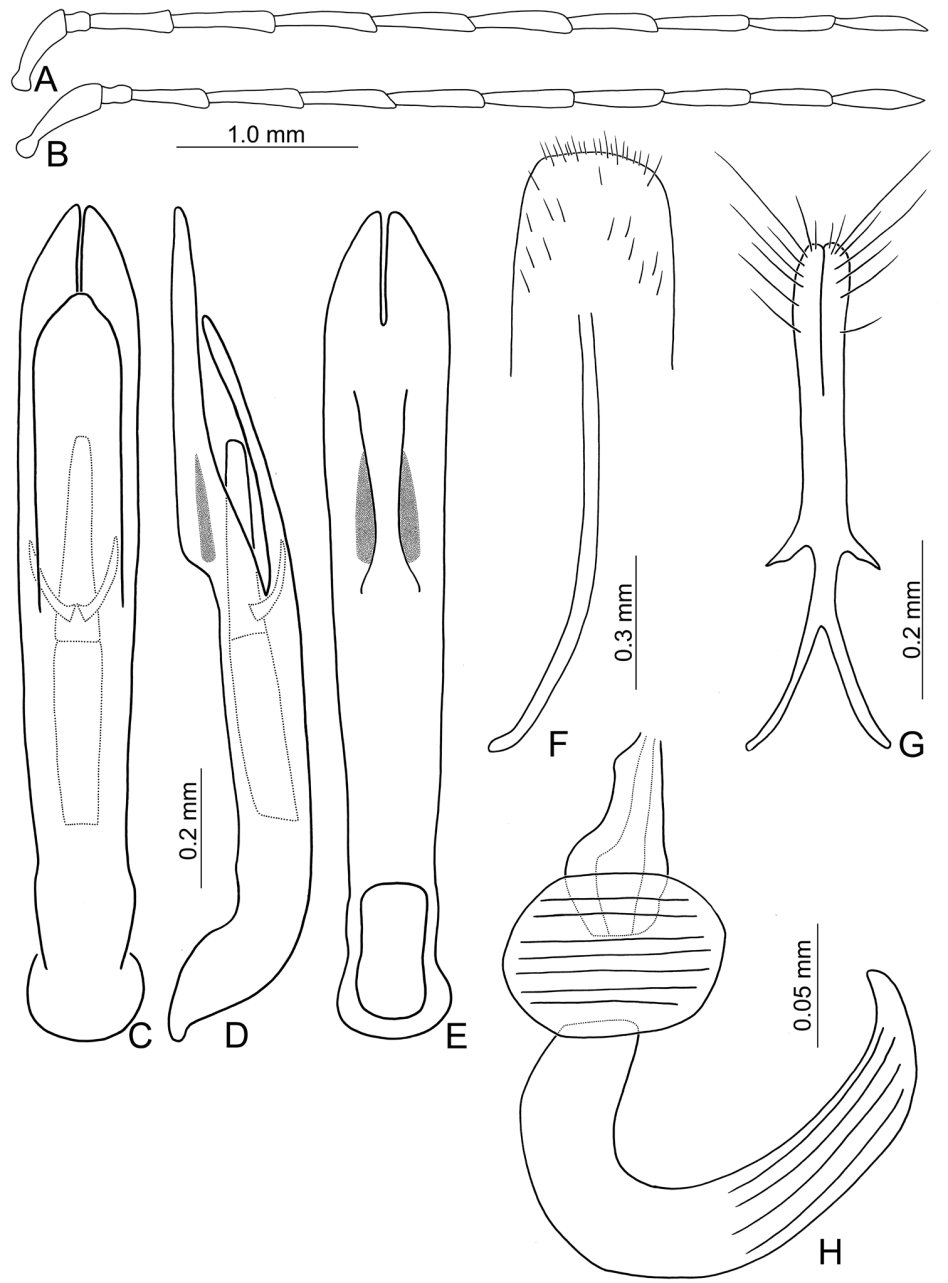
Diagnostic characters of *Pseudotheopea hsingtzungi* sp. nov. **A** Antenna, male **B** Antenna, female **C** Aedeagus, dorsal view **D** Aedeagus, lateral view **E** Aedeagus, ventral view **F** Abdominal ventrite VIII **G** Gonocoxae **H** Spermatheca.

Figs 15A–C ; 16A, B ; 17

Theopea sauteri : Medvedev, 2000: 178 (part, misidentification).

##### Types.

Holotype ♂ (NHMUK), LAOS. Hua Phan: Ban Saluei, Phou Pane (Mt.), 20°12’N 104°01’E, 1300-1900 m, 3-30.IV.2014, leg. C. Holzschuh. Paratypes. **LAOS**. Champassak: 1♀ (HNHM), Dong Hua Xao NBCA, bank of Nam Phak river, 15°59’N 105°55’E, 280 m, 28-29.III.1998, leg. O. Merkl and G. Csorba (identified as *Theopea sauteri* by Medvedev (2000)); Hua Pan: 1♀ (JBCB), Ban Kangpabong env., 25km SE Vieng Xai (by road), 20°19’N 104°25’E, 14-18.V.2001, leg. J. Bezděk; 1♀ (NHMB), Phou Pane Mt., 20°13’N 104°00’E, 1350-1500 m, 1-16.VI.2009, leg. M. Brancucci; Oudomxai: 1♀ (NHMB), Oudom Xai (17 km NEE), 20°45’N 102°09’E, 1100 m, 1-9.V.2002, leg. V. Kubáň.

##### Description.

Length 5.8–6.2 mm, width 2.1–2.4 mm. Body color (Fig. 15A–C) golden green, but mouthparts and legs yellowish brown, antennae dark brown. Frontoclypeus (Figs 16A–16B) with transverse deep groove between eyes in males, concavity 0.5× as wide as interspace between eyes. Antennae filiform in males (Fig. 17A), antennomere I smaller than others, length ratios of antennomeres I–XI 1.0: 0.3: 1.0: 1.2: 1.2: 1.2: 1.2: 1.1: 1.1: 1.0: 1.1, length to width ratios of antennomeres I–XI 3.3: 1.2: 3.8: 4.8: 5.7: 5.5: 5.5: 5.4: 6.1: 5.6: 6.2; similar but slightly shorter in females (Fig. 17B), length ratios of antennomeres I–XI 1.0: 0.3: 0.8: 0.9: 0.9: 0.9: 0.9: 0.9: 0.9: 0.8: 0.9, length to width ratios of antennomeres I–XI 3.6: 1.7: 4.0: 4.9: 5.3: 5.3: 5.1: 5.4: 5.3: 4.9: 4.7. Elytra elongate, parallel-sided, 1.8-1.9× longer than wide; disc with dense, coarse punctures, arranged into longitudinal rows, with one distinct longitudinal ridge between two longitudinal rows of punctures. Tarsomeres I of front legs swollen in males; subparallel in females. Aedeagus (Fig. 17C–E) extremely slender, 7.2× longer than wide; apex with deep notch; tectum elongate, from apical 1/9 to middle; almost straight in lateral view, moderately curved near base, angular at apical 2/5; triangular sclerites elongate; internal sac with elongate, endophallic sclerite complex, 0.5× as long as aedeagus, composed of two sclerites, apical piece as long as basal piece, one pair of dorsal sclerite hook-like, connected near base of apical piece; ventral sclerites absent. Gonocoxae (Fig. 17G) elongate, both gonocoxae fused from basal 1/4 to apical 1/4; apices convergent and narrowly rounded, each gonocoxa with eight setae along lateral margin from apex to apical 1/6; with one pair of short lateral processes at basal 2/5. Ventrite VIII (Fig. 17F) elongate and well sclerotized; disc with several long setae at sides and near apical margin, and with dense, short setae along apical margin; spiculum extremely slender. Receptacle of spermatheca (Fig. 17H) strongly swollen; pump slender and strongly curved; proximal spermathecal duct shallowly inserted into receptacle, narrow and short.

##### Diagnosis.

*Pseudotheopea hsingtzungi* sp. nov. (Fig. 15A–C), *P. boreri* sp. nov. (Fig. 8A–C), *P. clypealis* (Medvedev) (Fig. 8D–F), and *P. smaragdina* (Gressitt and Kimoto) (Fig. 15D–F), are characterized by their golden green coloration. They can be identified based on their distribution: *P. boreri* sp. nov. from India, *P. clypealis* from Vietnam, *P. hsingtzungi* sp. nov. from Laos, and *P. smaragdina* from China. *Pseudotheopea hsingtzungi* sp. nov. (Fig. 15A, B) is similar to *P. boreri* sp. nov. (Fig. 8A, C) and *P. smaragdina* (Fig. 15D, E) based on the shared indistinct longitudinal ridges on the elytra (convex and distinct longitudinal ridges on the elytra in *P. clypealis* (Fig. 8D, F)), but differs by having the concavity narrower between the eyes in males (Fig. 16A, B) (concavity wide between eyes in others (Figs 9A, B; 16C, D)). Males of *P. hsingtzungi* sp. nov. are similar to those of *P. kimotoi* sp. nov. in possessing elongate triangular aedeagal sclerites (Figs 17D, E; 20E, F) but differ in the presence of one pair of long hook-like lateral sclerites of the median elongate sclerite, lacking small spines near the apex of the median elongate sclerite, and the median division (Fig. 17C, D) (lacking hook-like sclerites at sides of median elongate sclerite, with small spines near apex of median elongate sclerite, and undivided in those of *P. kimotoi* sp. nov. (Fig. 20D, E)).

##### Etymology.

The new species is dedicated to Mr. Hsing-Tzung Cheng, who is a member of the Taiwan Chrysomelid Research Team (TCRT) for inventorying leaf beetles.

##### Distribution.

Laos.

#### Pseudotheopea kimotoi sp. nov.

Taxon classificationAnimaliaColeopteraChrysomelidae

D3A047A9-2396-570E-A75D-E9C3F536B26A

http://zoobank.org/5A493FF3-7DD0-4DCB-81FF-893F6FCA9420

**Figure 18.**
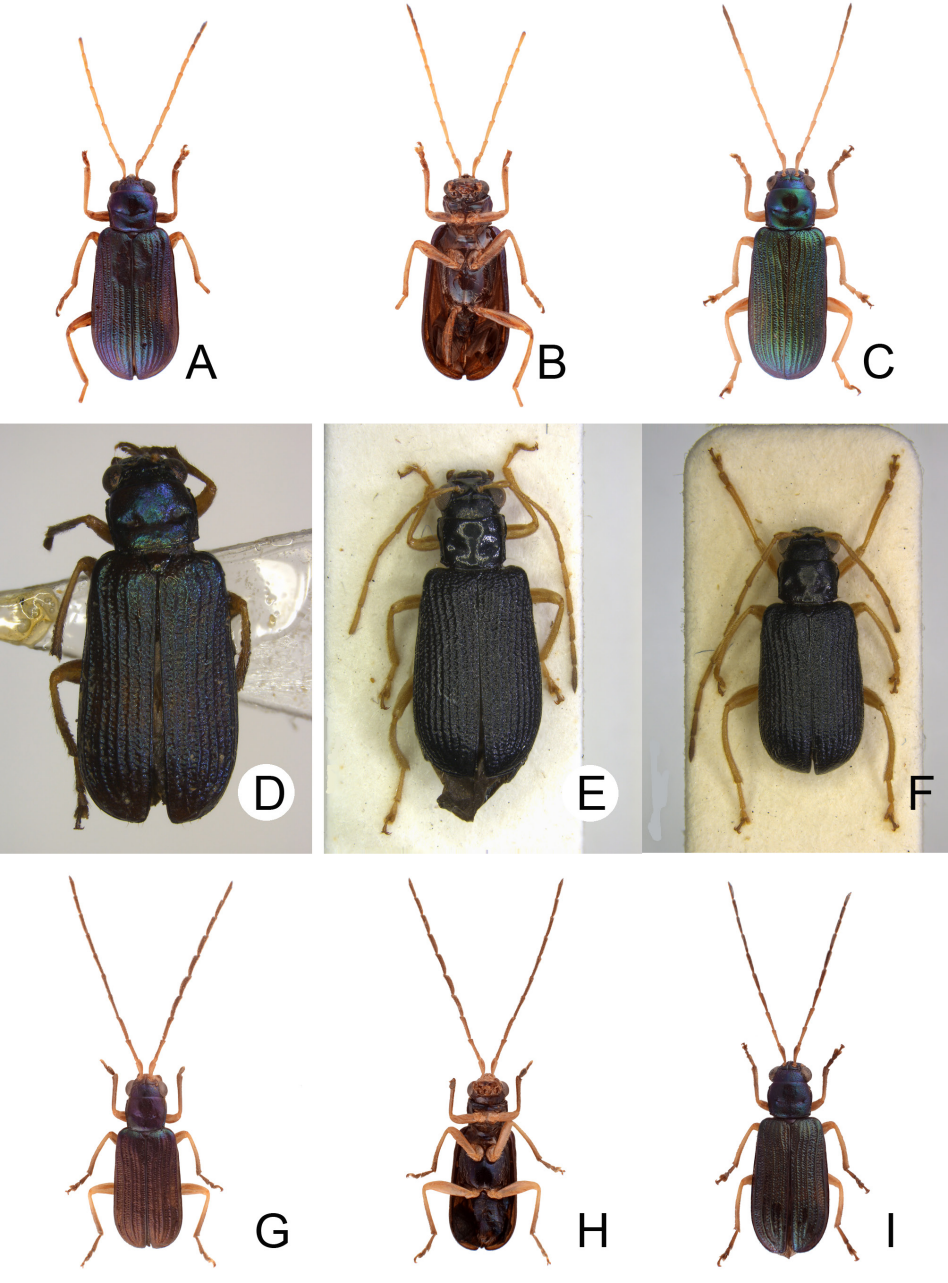
Habitus of *Pseudotheopea kimotoi* sp. nov., *P. nigrita*, and *P. leehsuehae* sp. nov. **A***P. kimotoi* sp. nov., male, dorsal view **B** Same, ventral view **C***P. kimotoi* sp. nov., female, dorsal view **D***P. kimotoi* sp. nov., male, from Dalat **E***P. nigrita*, holotype, dorsal view **F***P. nigrita*, male, dorsal view **G***P. leehsuehae* sp. nov., male, dorsal view **H** Same, ventral view **I***P. leehsuehae* sp. nov., female, dorsal view.

**Figure 19.**
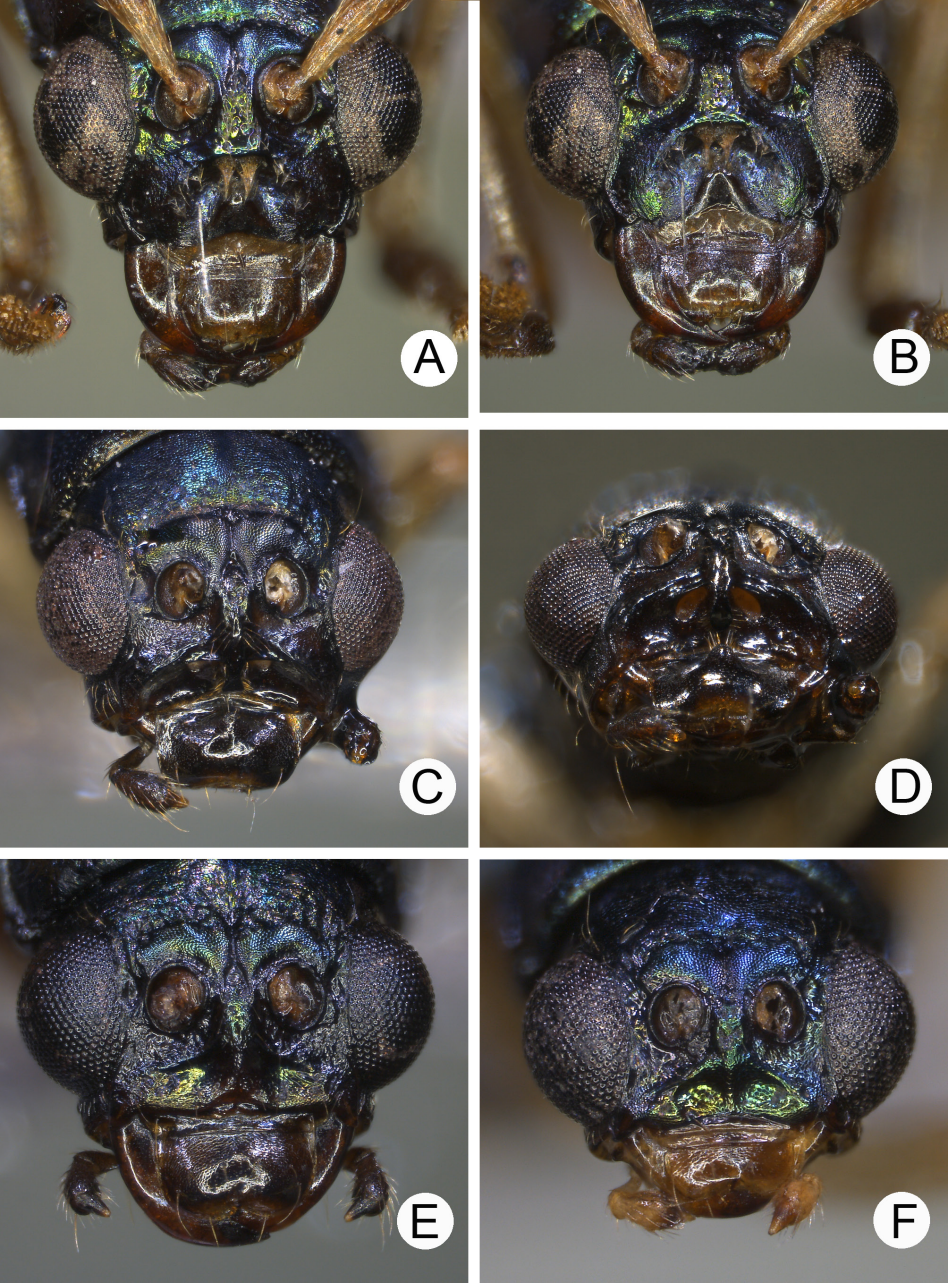
Heads of *Pseudotheopea kimotoi* sp. nov., *P. sufangae* sp. nov., and *P. sauteri*. **A***P. kimotoi* sp. nov., male, dorsofrontal view **B** Same, front view **C***P. sufangae* sp. nov., male, dorsofrontal view **D** Same, front view **E***P. sufangae* sp. nov., female, dorsofrontal view **F***P. sauteri*, female, dorsofrontal view.

**Figure 20.**
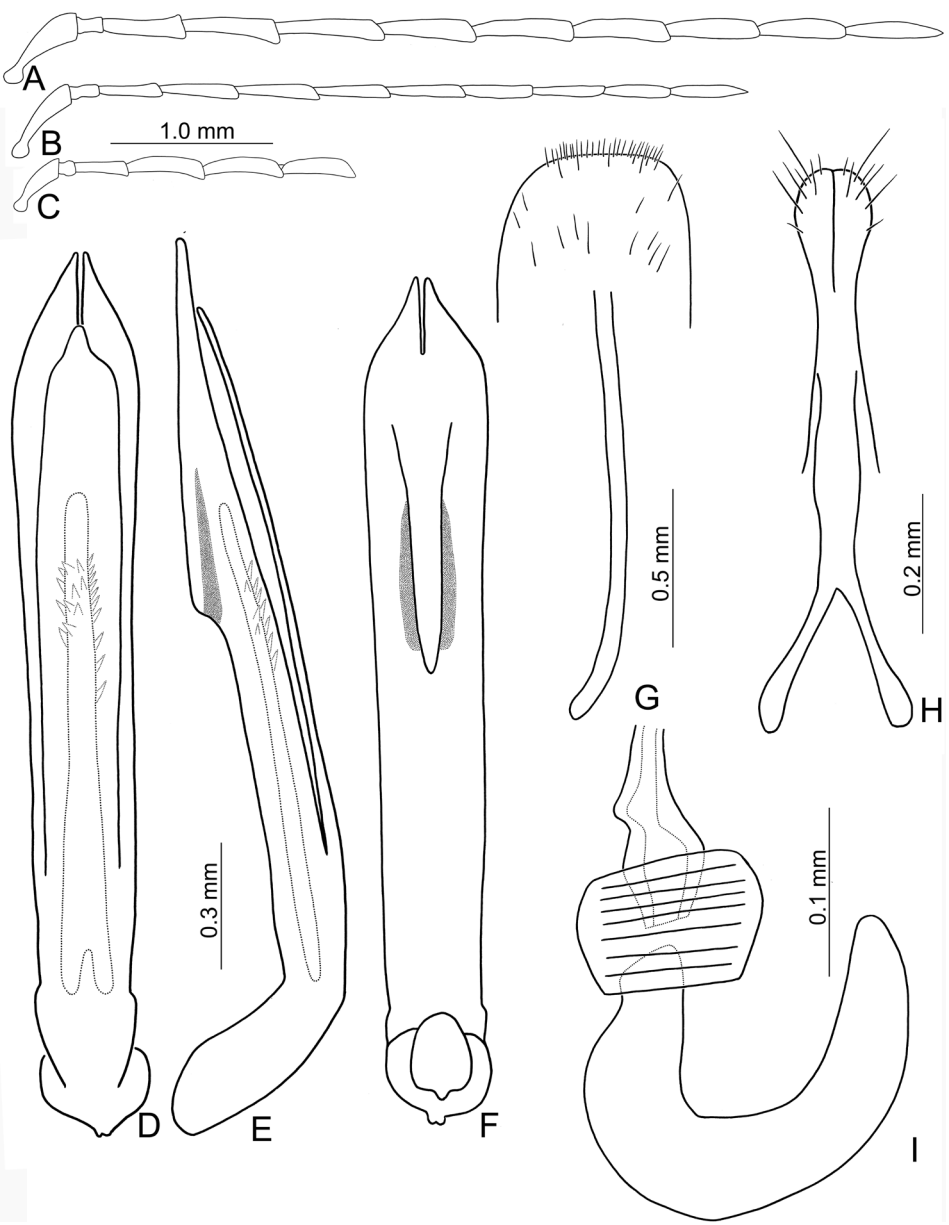
Diagnostic characters of *Pseudotheopea kimotoi* sp. nov. **A** Antenna, male **B** Antenna, female **C** Antenna, male, from Dalat **D** Aedeagus, dorsal view **E** Aedeagus, lateral view **F** Aedeagus, ventral view **G** Abdominal ventrite VIII **H** Gonocoxae **I** Spermatheca.

Figs 18A–D ; 19A, B ; 20

Theopea sauteri : Kimoto, 1989: 200 (part); Medvedev, 2000: 178 (part).

##### Types.

Holotype ♂ (BPBM): “VIET NAM: 7km SE / of Dilinh (Djiring) / 990m, 2.V.1960 [p, w] // ♂ [p, w] // R. E. Leech / Collector / BISHOP [p, w] // Theopea / sauteri / Chujo [h] / Det. S. Kimoto, 19 [p] 87 [h, w]”. This specimen was misidentified as *Theopea sauteri* by Kimoto (1989). Paratypes. **LAOS**. Boli Kham Xai: 1♀ (JBCB), Ban Nape (8km NE), ~600 m, 18°21’N 105°08’E, 1-18.V.2001, leg. C. L. Peša; Champassak: 1♂, 5♀♀ (HNHM), Dong Hua Xao NBCA, 2 km S of Ban Nong Luang, bank of Touay-Guai Stream, 15°04’N 106°13’E, 800 m, 1-5.IV.1998, leg. O. Merkl and G. Csorba (identified as *Theopea sauteri* by Medvedev (2000)); Hua Pan: 3♀♀ (JBCB), Ban Kangpabong env., 25km SE Vieng Xai (by road), 20°19’N 104°25’E, 14-18.V.2001, leg. J. Bezděk; Khammouane: 2♀♀ (RBCN), Nakai env., Rout no 8, 17°42.8’N 105°09.1’E, 560 m, 4-8.V.1998, leg. E. Jendek and O. Šauša; Louangphrabang: 4♀♀ (NHMB), Thong Khan, 19°33’N 101°58’E, 750 m, 11-21.V.2002, leg. V. Kubáň; **THAILAND**. Loei: 1♀ (NMPC), Phu Kradung N.P., 16-17.V.1999, leg. D. Hauck; **VIETNAM**. 1♀ (ZSM), Tam Dao, 1982, leg. L. Medvedev; Cao Bang: 1♂ (NMPC), Bao-Lac; Lam Dong: 1♂, 1♀ (BPBM), 6 km S Dalat, 1400-1500 m, 6.VI.-7.VII.1961, leg. N. R. Spencer, identified as *Theopea sauteri* by Kimoto (1989); Ninh Binh: 1♀ (NHMB), Cuc Phuong N.P., 21-27.V.1996, leg. Pacholátko and Dembický.

##### Description.

Length 6.6-7.5 mm, width 2.6-3.2 mm. Body color (Fig. 18A–D) metallic blue or purple, but antennae and legs yellowish brown, mouth parts dark brown. Frontoclypeus (Fig. 19A, B) transverse and weakly excavated between eyes in males, semi-circular, the annular concavity 0.8× as wide as interspace between eyes, with cluster of long setae near middle of anterior margin, some shorter setae scattered along anterior margin. Antennae filiform in males (Fig. 20A), but relatively broader than those of females (Fig. 15A), length ratios of antennomeres I–XI 1.0: 0.3: 0.9: 1.1: 1.2: 1.0: 1.1: 1.0: 1.0: 0.9: 1.1, length to width ratios of antennomeres I–XI 3.3: 1.6: 3.4: 4.4: 4.9: 4.2: 4.8: 4.5: 4.7: 4.8: 4.7; filiform in females (Fig. 20B), length ratios of antennomeres I–XI 1.0: 0.3: 0.7: 0.9: 1.0: 0.9: 0.9: 0.8: 0.8: 0.8: 0.9, length to width ratios of antennomeres I–XI 4.2: 2.2: 4.1: 5.1: 5.6: 5.4: 5.7: 5.1: 5.5: 5.2: 6.2. Elytra elongate, parallel-sided, 1.7-1.9× longer than wide; disc with dense, coarse punctures, arranged into longitudinal rows, with one distinct and convex longitudinal ridge between two longitudinal rows of punctures, with convex area behind scutellum, ridges reduced at convex area. Tarsomeres I of front legs strongly swollen in males; subparallel in females. Aedeagus (Fig. 20D–F) slender, 8.4× longer than wide; apex with shallow notch; tectum elongate, from apical 1/13 to basal 1/3; almost straight in lateral view, angular at apical 2/5, moderately curved near base; triangular sclerites elongate; internal sac covered with stout teeth, with elongate endophallic sclerite, 0.5× as long as aedeagus, some small, stout teeth at apical 1/8 to 2/5. Gonocoxae (Fig. 20H) elongate, both gonocoxae fused from basal 1/4 to apical 1/5; apices convergent and narrowly rounded, each gonocoxa with eight setae along lateral margin from apex to apical 1/6, four much longer than others; lateral processes reduced. Ventrite VIII (Fig. 20G) elongate and well sclerotized; disc with several long setae at sides and near apical margin, and with dense, short setae along apical margin; spiculum extremely slender. Receptacle of spermatheca (Fig. 20I) strongly swollen; pump slender and strongly curved; proximal spermathecal duct deeply inserted into receptacle, narrow and short.

##### Variation.

One male collected from Dalat has a smaller body (5.3 mm long, 2.2 mm wide) and the convex area on the elytra is indistinct and with longitudinal ridges (Fig. 20D), antennomeres IV-VI are curved (VII-XI lost, Fig. 20C), and the frontoclypeus lacks a concavity.

##### Diagnosis.

*Pseudotheopea kimotoi* sp. nov. is similar to *P. clypealis* (Medvedev) and *P. leehsuehae* sp. nov. based on the convex and distinct longitudinal ridges on the elytra but differs in having all longitudinal ridges convex and distinct (Fig. 18A, C, D) (intertwined with convex distinct ridges and weak indistinct ones in others (Figs 8D, F; 18F, H), with a convex area surrounding the scutellum and longitudinal ridges reduced on the convex area in males (Fig. 18A) (without convex area surrounding scutellum in those of others (Figs 8D, 18F)) and the shallow concavity between it in males (Fig. 19A, B) (deep concavity in others (Figs 9C, D; 13C, D)). Males of *P. kimotoi* sp. nov. (Fig. 20E, F) are similar to those of *P. hsingtzungi* sp. nov. (Fig. 17D, E) in possessing elongate triangular aedeagal sclerites but differ in the absence of lateral sclerites attached to the median elongate sclerite, with small spines near apex of median elongate sclerite, which is undivided (Fig. 20D, E) (with one pair of hook-like sclerites at sides of median elongate sclerite, without small spines near apex of median elongate sclerite, and divided at middle in those of *P. hsingtzungi* sp. nov. Fig. 17C, D)).

##### Etymology.

This new species is dedicated to late Dr. Shinsaku Kimoto for his great contribution to taxonomy of oriental and Palaearctic Chrysomelidae.

##### Distribution.

Laos, Thailand, Vietnam.

#### Pseudotheopea leehsuehae sp. nov.

Taxon classificationAnimaliaColeopteraChrysomelidae

8F7275A9-7EDE-5B63-B1DA-6F55E80F6608

http://zoobank.org/4A9146B2-EDC1-4E51-9C51-5109CA37CB18

**Figure 21.**
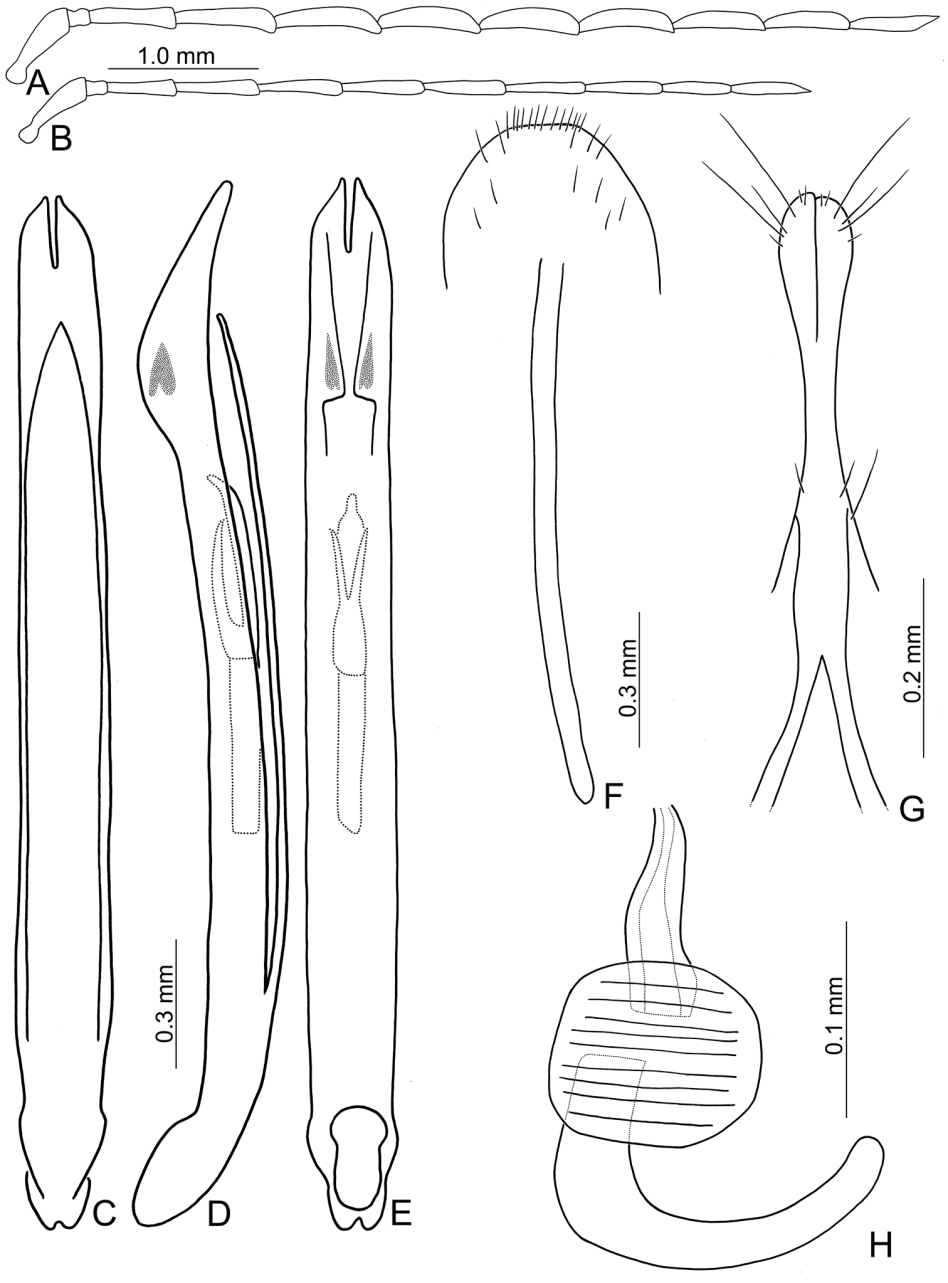
Diagnostic characters of *Pseudotheopea leehsuehae* sp. nov. **A** Antenna, male **B** Antenna, female **C** Aedeagus, dorsal view **D** Aedeagus, lateral view **E** Aedeagus, ventral view **F** Abdominal ventrite VIII **G** Gonocoxae **H** Spermatheca.

Figs 13C, D ; 18F–H ; 21

##### Types.

Holotype ♂ (NHMB), LAOS, Louang Namtha, Namtha → Muang Sing, 21°09’N 101°19’E, 900-1200 m, 5-31.V.1997, leg. V. Kubáň. Paratypes. 5♂♂ (NHMB), same as holotype; **LAOS**. Houa Phan: 1♂ (NHMB), Ban Saluei → Phou Pane Mt., 20°12-13.5’N 103°59.5’-104°01’E, 1340-1870 m, 10.V.-16.VI.2009, leg. M. Brancucci and local coll.; Louang Namtha: 1♂ (NMPC), 20 km BW Louang Namtha, 21°09.2’N 101°18.7’E, 800-1100 m, 5.-11.V.1987, leg. M. Štrba and Hergovits; Phongsali: 1♂ (JBCB), Boun Tai (10km SE), 16-25.V.2004, leg. Lao collector; Xaisomboun: 1♀ (NMPC), Phou Khao Khouay N.P., Tad Leuk, 18°23’N 103°04’E, 150-200 m, 15.-21.V.2001, leg. E. Jendek and O. Šauša

##### Description.

Length 4.8–5.9 mm, width 1.8–2.4 mm. Body color metallic purple (Fig. 18F–H), legs yellowish brown, tarsi darker; mouth parts and antennae dark brown or blackish brown. Frontoclypeus (Fig. 13C, D) transverse and deeply excavated between eyes in males, concavity transverse and as wide as interspace between eyes; with small membranous sclerite covering sides; membranous sclerite covering most shallow areas of concavity, also with one pair of erect membranous sclerites, concavity with short hair-like setae along margin. Antennae filiform in males (Fig. 21A), relatively broader than females, antennomeres III-X slightly curved, length ratios of antennomeres I–XI 1.0: 0.2: 1.0: 1.1: 1.2: 1.1: 1.1: 1.1: 1.0: 1.0: 1.0, length to width ratios of antennomeres I–XI 3.3: 1.3: 4.0: 4.8: 4.9: 4.8: 5.0: 5.1: 5.1: 4.9: 5.5; slender and straight in females (Fig. 21B) length ratios of antennomeres I–XI 1.0: 0.3: 0.8: 1.0: 1.0: 1.0: 1.0: 1.0: 1.0: 1.0: 1.0, length to width ratios of antennomeres I–XI 3.4: 1.5: 3.9: 5.5: 5.7: 5.4: 5.9: 6.5: 6.2: 5.4: 6.5. Elytra elongate, parallel-sided, 1.7–1.9× longer than wide; disc with dense, coarse punctures arranged into longitudinal rows, with convex longitudinal ridges between rows of punctures, distinct and indistinct ridges intertwined. Tarsomeres I of front legs swollen in males; subparallel in females. Aedeagus (Fig. 21C–E) extremely slender, 11.9× longer than wide; parallel-sided; apex with shallow notch, both apices equal in length; tectum elongate, from apical 1/9 to basal 1/4; slightly curved in lateral view, angular at apical 1/5; triangular sclerites small; internal sac with elongate, endophallic sclerite complex, 0.3× as long as aedeagus, composed of two sclerites, apical piece as long as basal piece, with one pair of hook-like sclerites combined at base of apical piece ventrally. Gonocoxae (Fig. 21G) elongate; apices convergent and narrowly rounded, each gonocoxa with six setae along lateral margin from apex to apical 1/6, three much longer than others; lateral processes reduced, with one or two setae near base; base membranous. Ventrite VIII (Fig. 21F) elongate and well sclerotized; disc with several long lateral setae, and with dense, short setae along apical margin; spiculum extremely slender. Receptacle of spermatheca (Fig. 21H) strongly swollen; pump slender and strongly curved; proximal spermathecal duct deeply inserted into receptacle, narrow and short.

##### Diagnosis.

*Pseudotheopea leehsuehae* sp. nov. (Fig. 18F, H) is similar to *P. clypealis* (Medvedev) (Fig. 8D, F) based on the convex, distinct ridges and weak indistinct ridges intertwined on the elytra, but *P. leehsuehae* sp. nov. can be separated from *P. clypealis* by its metallic purple body (Fig. 18F, H) (golden green body in *P. clypealis* (Fig. 8D, F)), and concavity covered with a membranous sclerite and with one pair of erect rounded sclerites (Fig. 13C, D) (without such structures in *P. clypealis* (Fig. 9C, D)). Males of *P. leehsuehae* sp. nov. are characterized by their extremely elongate aedeagi (11.7× longer than wide) and one pair of hook-like sclerites arising from the middle of the ventral surface of the apical piece of the endophallic sclerite complex (Fig. 21C–E).

##### Etymology.

The new species is dedicated to Mrs. Hueh Lee, who is a member of the Taiwan Chrysomelid Research Team (TCRT) for inventorying leaf beetles.

##### Distribution.

Laos.

#### Pseudotheopea smaragdina

Taxon classificationAnimaliaColeopteraChrysomelidae

(Gressitt & Kimoto, 1963) comb. nov.

7045C69F-DA63-5BD4-83F1-2B0CB8EAC3D0

**Figure 22.**
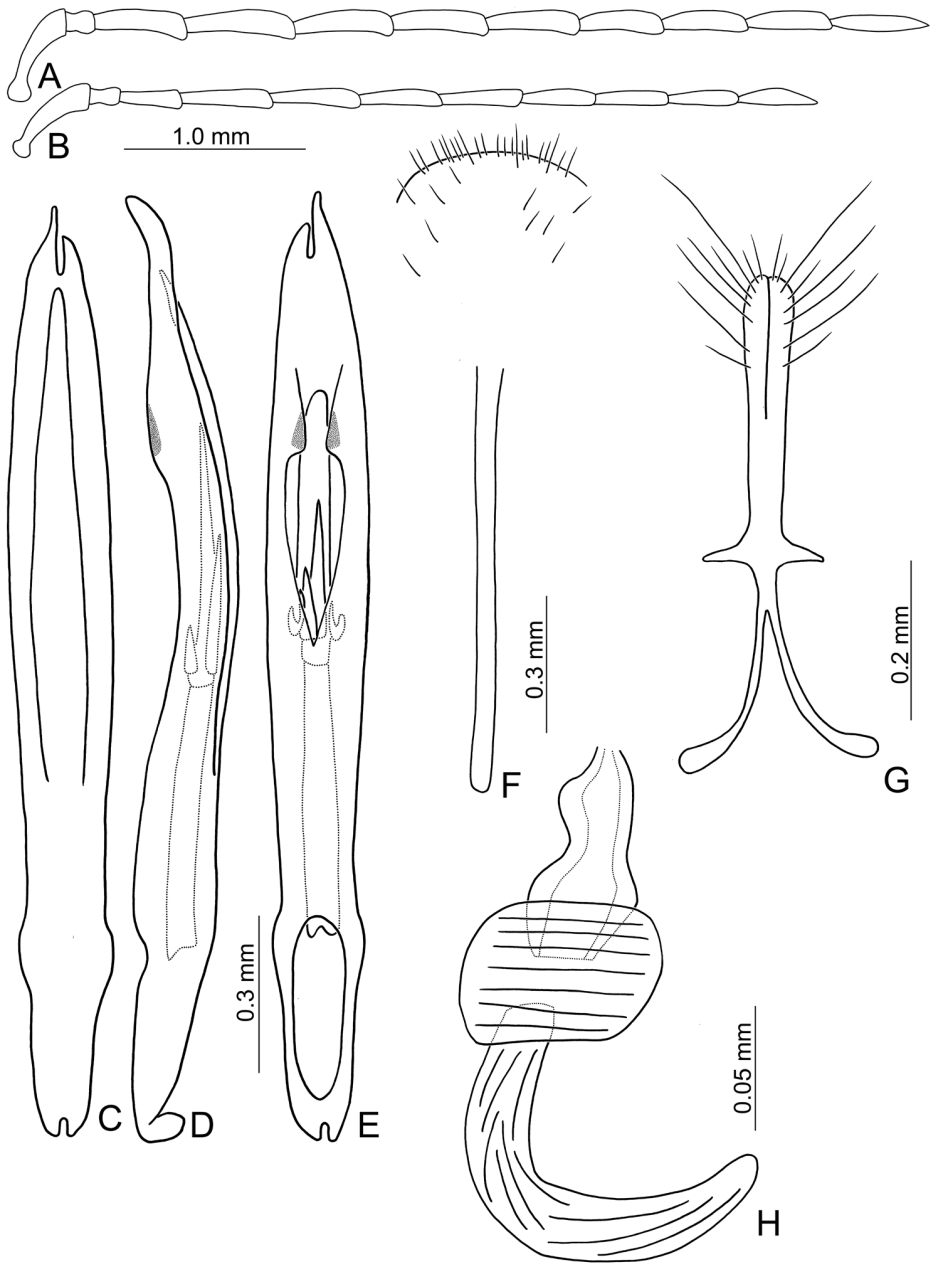
Diagnostic characters of *Pseudotheopea smaragdina*. **A** Antenna, male **B** Antenna, female **C** Aedeagus, dorsal view **D** Aedeagus, lateral view **E** Aedeagus, ventral view **F** Abdominal ventrite VIII **G** Gonocoxae **H** Spermatheca.

Figs 15D–F ; 16C, D ; 22

Theopea smaragdina Gressitt & Kimoto, 1963: 680 (China: Hainan Island, Guandong); Wilcox, 1973: 631 (catalogue); Wang *et al*., 1998: 129 (China: Fujian: Wuyishan); Yang, 2002: 656 (China: Fujian); Yang & Yao, 2002: 447 (China: Hainan Island); Aston, 2009: 24 (Hong Kong); Beenen, 2010, 489 (catalogue).

##### Types.

Holotype ♂ (CAS): “HAINAN I. China / Tahau VIII [p] 6 [h] 1935 / L. Gressitt [p, w] // L. Gressitt / Collection [p, w] // HOLOTYPE [p] ♂ / Theopea / smaragdina [h] / Gressitt and Kimoto [p, r] // Theopea holo- / sp. nov. 2 / smaragdina [h] / Det. S. Kimoto [p] G and K [h, w]” // California Academy / of Sciences / Type / No. [p] 12422 [h, w]”. Paratypes. 1♀ (NHMUK): “Para- / type [p, w, circle label with yellowish border] // CHINA: Kwang- / tung [= Guangdong], Fei-ha-fei- / loi. VII-1-1956 / J. L. Gressitt [p, w] // Brit. Mus. / 1963-245. [p, w] // L. Gressitt / Collection [p, w] // PARATYPE [p] / Theopea / smaragdina [h] / Gressitt and Kimoto [p, y] // Theopea / smaragdina / G and K [h] / Gressitt and Kimoto det. 196[p] 2[h]”; 1♂ (MNHUB): “China, Canton, [p] / Fati 10.V.10 [h] / Mell S. V. [p, y] // PARATYPE [p] / Theopea / smaragdina [h] / Gressitt and Kimoto [p, y]”; 1♀ (CAS): “HAINAN I. China / Tahau. VII[p] 6[h] 1935 / L. Gressitt [p, w] // L. Gressitt / Collection [p, w] // PARATYPE [p] / Theopea / smaragdina [h] / Gressitt and Kimoto [p, y] // Theopea / smaragdina / G and K [h] / Gressitt and Kimoto det. 196[p]2 [h, w]”.

##### Other specimens examined.

**CHINA**. Guangdong: 1♂ (SEHU), 廣州 (Guangzhou), 16.IV.1983, leg. A. Tanaka; 6♀♀ (NMPC), Guangzhou, Baiyunshan vill., 23°09’47”-10’30”N 113°13’27”-17’44”E, 50-250 m, 27.VI.2014, leg. J. Hájek, J. Růžička and M. Tkoč; Hong Kong: 1♀ (PAHC), Nam Chung, 8.V.2009, leg. P. Aston; 9♀♀ (BPBM), Soko island, Tai-A-Chan, 23-25.V.1988, coll. C. O’Connell; 2♂♂, 1♀ (PAHC), Sha Lo Tung, 10.V.2012, leg. P. Aston; 1♀ (PAHC), same but with “3.V.2014”.

##### Redescription.

Length 5.7–6.7 mm, width 2.2–2.4 mm. Body color (Fig. 15D–F) golden green, but antennae, mouth parts, and legs yellowish brown, five or six apical antennomeres darker. Frontoclypeus (Fig. 16C, D) transverse and deeply excavated between eyes in males, concavity as wide as interspace between eyes; with one erect process at center, apically tapering; one pair of membranous areas surrounding erect process, mesally connected; with several erect hair-like setae at sides of anterior margin. Antennae filiform in males, but relatively broader than those of female (Fig. 22A), antennomeres III-IX slightly curved, length ratios of antennomeres I–XI 1.0: 0.3: 0.9: 1.1: 1.0: 0.9: 0.9: 0.8: 0.8: 0.8: 1.0, length to width ratios of antennomeres I–XI 3.8: 1.3: 3.4: 4.1: 4.5: 4.2: 4.1: 4.1: 4.3: 4.6: 6.4; filiform in females (Fig. 22B), length ratios of antennomeres I–XI 1.0: 0.3: 0.6: 1.0: 1.0: 0.9: 0.9: 0.8: 0.8: 0.7: 0.9, length to width ratios of antennomeres I–XI 3.4: 1.7: 3.1: 4.7: 4.6: 5.0: 4.8: 4.9: 4.9: 4.6: 5.3. Elytra elongate, parallel-sided, 1.8-1.9× longer than wide; disc with dense, coarse punctures, arranged into longitudinal rows, with distinct longitudinal ridge between two longitudinal rows of punctures. Tarsomeres I of front legs swollen in males; subparallel in females. Aedeagus (Fig. 22C–E) extremely slender, 9.4× longer than wide; apex with shallow notch, both apices not equal in length; tectum elongate, from apical 1/12 to basal 2/5; almost straight in lateral view, apically curved, angular at apical 1/4; triangular sclerites small; internal sac with elongate, endophallic sclerite complex, 0.6× as long as aedeagus, composed of two sclerites, apical piece as long as basal piece, two dorsal sclerites unequal in length; ventral sclerites present. Gonocoxae (Fig. 22G) elongate, both gonocoxae fused from basal 1/4 to apical 1/3; apices convergent and narrowly rounded, each gonocoxa with eight setae along lateral margin from apex to apical 1/6; with one pair of short lateral processes at basal 2/5. Ventrite VIII (Fig. 22F) longitudinal and well sclerotized; disc with several long setae at sides and near apical margin, and with dense, short setae along apical margin; spiculum extremely slender. Receptacle of spermatheca (Fig. 22H) strongly swollen; pump slender and strongly curved; proximal spermathecal duct deeply inserted into receptacle, narrow and short.

##### Diagnosis.

*Pseudotheopea smaragdina* (Gressitt and Kimoto) (Fig. 15D–F), *P. boreri* sp. nov. (Fig. 8A–C), *P. clypealis* (Medvedev) (Fig. 8D–F), and *P. hsingtzungi* sp. nov. (Fig. 15A–C), are characterized by their golden green coloration. They can be identified based on their distribution: *P. boreri* sp. nov. from India, *P. clypealis* from Vietnam, *P. hsingtzungi* sp. nov. from Laos, and *P. smaragdina* from China. *Pseudotheopea smaragdina* (Fig. 15D, E) is similar to *P. hsingtzungi* sp. nov. (Fig. 15A, B) and *P. boreri* sp. nov. (Fig. 8A, 8C) in sharing the indistinct longitudinal ridges on the elytra (convex and distinct longitudinal ridges on the elytra in males *P. clypealis*), but it differs by having a wider concavity between the eyes bearing one erect process in males (Fig. 16C, D) (concavity wide between eyes without erect processes in males of *P. boreri* sp. nov. (Fig. 9A, B); concavity narrowed between eyes and without erect processes in males of *P. hsingtungi* sp. nov. (Fig. 16A, B)). Males of *P. smaragdina* are similar to those of *P. clypealis* with one additional elongate aedeagal sclerite and one pair of small lateral hook-like sclerites inside the internal sac (Figs 11C, D; 22D, E). They differ in having asymmetrical apices of the aedeagus and a relatively shorter apical piece (as long as basal piece) of the median elongate sclerite (Fig. 22C–E) (symmetric apices and relatively longer apical piece, 4.0× as long as basal piece in *P. clypealis* (Fig. 11C–E)).

##### Distribution.

China (Hainan Island, Fujian, Hong Kong).

#### Pseudotheopea sufangae sp. nov.

Taxon classificationAnimaliaColeopteraChrysomelidae

271A3B7E-F122-5BD6-AA0F-DF31867FEEAF

http://zoobank.org/E2AAABD6-8244-4AAB-A48D-BE4AF1E1E9B2

**Figure 23.**
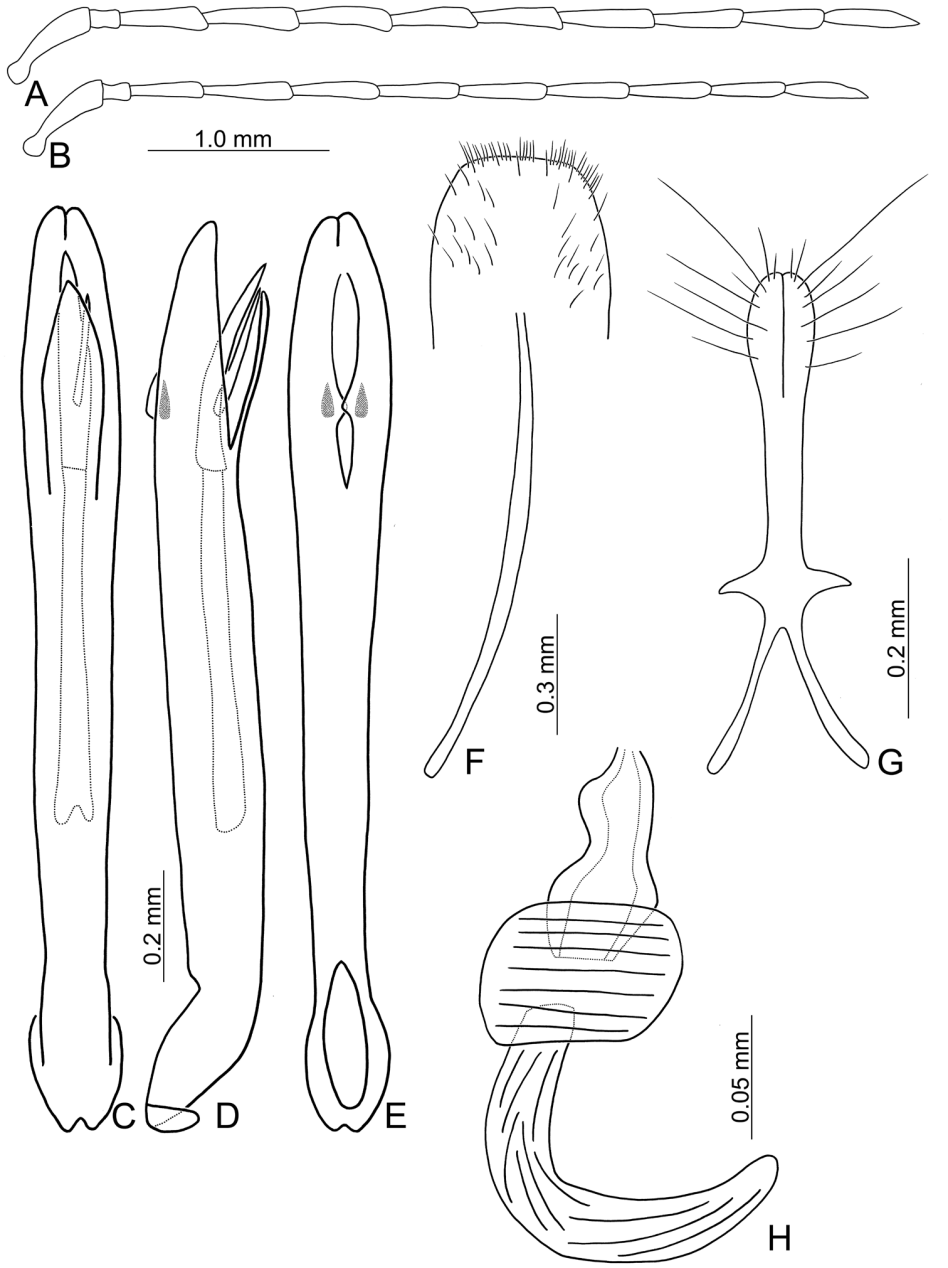
Diagnostic characters of *Pseudotheopea sufangae* sp. nov. **A** Antenna, male **B** Antenna, female **C** Aedeagus, dorsal view **D** Aedeagus, lateral view **E** Aedeagus, ventral view **F** Abdominal ventrite VIII **G** Gonocoxae **H** Spermatheca.

Figs 5F–H ; 19C–E ; 23

Theopea sauteri : Chûjô, 1962: 158 (misidentification).

##### Types.

Holotype ♂ (TARI), **TAIWAN**, Pingtung, Tahanshan (大漢山), 30.V.2014, leg. Y.-T. Chung; Paratypes. **TAIWAN**. Chiayi: 1♂ (TARI), Fenchihu (奮起湖), 25.V.2013, leg. W.-C. Liao; Hualien: 1♀ (NMPC), 15 km W of Yuli (玉里), 475 m, 7.VI.2008, leg. F. and L. Kantner; Ilan: 5♂♂ (HNHM), Fushan Botanical Garden (福山植物園), 8-11.IV.2002, leg. O. Merkl; 1♂, 8♀♀ (TARI), same locality, 3-9.VII.2013, leg. Y.-T. Wang; 1♀ (TARI), Songluoshan (松蘿山), 4.VI.2017, leg. Y.-T. Wang; Kaoshiung: 3♀♀ (NMNS), Shanping (扇平), 1.VI.1987, leg. C. W. and L. B. O’Brien; 1♂ (TARI), same locality, 11.IV.2015, leg. W.-C. Liao; 1♀ (TARI), Tengchih (藤枝), 7.IX.2012, leg. W.-C. Liao; 1♀ (TARI), same locality, 6.VIII.2013, leg. B.-X. Guo; 1♀ (TARI), same locality, 10.VIII.2013, leg. W.-C. Liao; Nantou: 1♀ (HNHM), Fuhosho (茅埔庄, = Wucheng 五城), VI.1909, leg. Sauter; 1♂ (NMNS), Howang (合望), 14-16.VIII.2002, leg. W.-T. Yang; 5♂♂, 7♀♀ (SEHU), Lienhwachih (蓮花池), 5-7.V.1978, leg. Y. Komiya; 4♀♀ (TARI), same locality, 23-26.V.1980, leg. K. S. Lin and B. H. Chen; 8♂♂, 11♀♀ (NMNS), same locality, 9.IV.-19.V.1998, leg. C. S. Lin and W. T. Wang; 2♀♀ (NMNS), same but with “6.VII.-12.VIII.1998”; 1♂ (NMNS), same but with “26.II.-21.III.2001”; 2♂♂ (NMNS), same but with “21.III.-9.IV.2001”; 10♂♂ (NMNS), same but with “2.V.-12.VI.2001”; 2♂♂ (NMNS), same but with “5.V.-10.VI.2002”; 1♂, 1♀ (NMNS), same but with “10.VI.-9.VII.2002”; 8♂♂, 2♀♀ (NMNS), same but with “4.III.-6.V.2003”; 4♀♀ (NMNS), same but with “6.V.-10.VI.2003”; 1♂, 1♀ (NMNS), same but with “10.VI.-7.VII.2003”; 1♂, 1♀ (NMNS), same but with “7.VII.-4.VIII.2003”; 1♀ (NMNS), same but with “4.VIII.-8.IX.2003”; 7♂♂, 8♀♀ (NMNS), same but with “10.V.-12.VII.2004”; 2♂♂ (NMNS), same but with “13.XII.2004-10.I.2005”; 1♀ (NMNS), same but with “7.III.-11.IV.2005”; 4♂♂, 2♀♀ (NMNS), same but with “11.IV.-2.V.2005”; 1♂, 1♀ (NMNS), same but with “2.V.-6.VI.2005”; 1♀ (NMNS), same but with “6.VI.-4.VII.2005”; 1♀ (TARI), same locality, 10.III.2013, leg. W.-C. Liao; 1♂ (SEHU), Nanshanchi (南山溪), 12.V.1977, leg. J. Ito; 3♂♂ (SEHU), same locality, 8.V.1978, leg. Y. Komiya; Pingtung: 1♂ (TARI), Lanren River (欖仁溪), 7.IV.2012, leg. Y.-H. Peng and Y.-C. Lan; 1♂ (TARI), Nanjenshan (南仁山), 4.III.2010, M.-L. Jeng; 1♂, 1♀ (TARI), same locality, 27.III.-5.IV.2010, leg. M.-L. Jeng; 1♀ (TARI), same locality, 18.IV.2010, leg. M.-L. Jeng; 1♂ (TARI), Tahanshan (大漢山), 14.VIII.2011, leg. Y.-T. Wang; 1♀ (TARI), same locality, 25.V.2013, leg. Y.-T. Chung; 1♂, 1♀ (TARI), same locality, 30.V.2013, leg. Y.-T. Chung; 1♂ (TARI), same locality, 9.VI.2013, leg. Y.-T. Chung; 1♀ (TARI), same locality, 3.VII.2013, leg. B.-X. Guo; 1♂ (TARI), same locality, 23.V.2014, leg. Y.-T. Chung; 1♀ (TARI), same locality, 30.V.2014, leg. Y.-T. Chung; Taipei: 3♂♂, 2♀♀ (HNHM), Neitong Forest Recreation Area (內洞森林遊憩區), 6 km S of Wulai (烏來), 7.IV.2002, leg. G. Fábián and O. Merkl; 1♀ (TARI), Pinglin (坪林), 6.V.2007, leg. S.-F. Yu; Taitung: 1♂, 1♀ (TARI), Chihpen (知本), 24.V.2013, leg. J.-C. Chen; 1♂ (TARI), Shouka (壽卡), 19.IV.2015, leg. W.-C. Liao; 1♀ (NMNS), Tyokakurai (= Chaochia, 紹家), 28.VII.1936, identified as *Theopea sauteri* by Chûjô (1962); Taoyuan: 1♂ (FREY), Monte Rara (= Lalashan, 拉拉山), VI.1939, leg. Arakawa.

##### Description.

Length 5.3–6.7 mm, width 2.3–2.8 mm. Body color (Fig. 5F–H) metallic blue or purple, antennae and legs yellowish brown, mouthparts dark brown. Frontoclypeus (Fig. 19C, D) transversely deeply excavated between eyes, concavity 0.8× as wide as interspace between eyes; with one longitudinal ridge from middle of anterior margin to basal 1/3, with hair-like setae along lateral margins of longitudinal ridge; one pair of membranous areas near sides of longitudinal ridge and anterior margin; with one small rounded process at center of labrum, disc with several hair-like setae. Antennae filiform in males, but relatively broader than those of females (Fig. 23A), length ratios of antennomeres I–XI 1.0: 0.3: 0.8: 0.9: 0.9: 0.9: 0.9: 0.9: 0.9: 0.8: 1.0, length to width ratios of antennomeres I–XI 4.2: 1.2: 3.6: 3.9: 4.2: 4.2: 4.2: 4.9: 4.6: 4.2: 5.8; filiform in females (Fig. 23B), length ratios of antennomeres I–XI 1.0: 0.3: 0.7: 0.9: 0.9: 0.8: 0.9: 0.8: 0.8: 0.7: 0.8, length to width ratios of antennomeres I–XI 3.6: 1.7: 3.9: 4.9: 5.3: 5.0: 5.3: 5.0: 4.8: 4.9: 5.5. Elytra elongate, parallel-sided, 1.7× longer than wide; disc with dense, coarse punctures, arranged into longitudinal rows, with one distinct longitudinal ridge between two longitudinal rows of punctures. Tarsomeres I of front legs slightly swollen in males; subparallel in females. Aedeagus (Fig. 23C–E) extremely slender, 9.7× longer than wide; apex with shallow incision; tectum short, from apical 1/12 to 1/4; almost straight in lateral view, slightly curved at base; triangular sclerites small; internal sac with elongate, endophallic sclerite complex, 0.6× as long as aedeagus, composed of two sclerites, apical piece (0.7×) much shorter than basal piece, dorsal sclerite well developed, 0.5× as long as apical piece. Gonocoxae (Fig. 23G) elongate, both goncoxae fused from basal 1/4 to apical 1/4; apices convergent and narrowly rounded, each gonocoxa with eight setae along lateral margin from apex to apical 1/6; with one pair of short lateral processes at basal 2/5. Ventrite VIII (Fig. 23F) elongate and well sclerotized; disc with several long setae at sides and near apical margin, and with dense, short setae along apical margin; spiculum extremely slender. Receptacle of spermatheca (Fig. 23H) strongly swollen; pump slender and strongly curved; proximal spermathecal duct deeply inserted into receptacle, narrow and short.

##### Diagnosis.

*Pseudotheopea sufangae* sp. nov. (Fig. 5F–H) is similar to *P. azurea* (Gressitt and Kimoto) (Fig. 5D, E) based on distinct but not convex ridges on the elytra. It differs by possessing a broad concavity between the eye with a median ridge (Fig. 19C, D) (narrow concavity between eyes and without ridge in *P. azurea* (Fig. 6A)). Males of *P. sufangae* sp. nov. are characterized by its incised aedeagal apex (with notch in other species), and presence of only one additional elongate dorsal sclerite near the base of the apical piece of the aedeagus (Fig. 23C–E). Females of *P. sufangae* sp. nov. are similar to those of the sympatric species, *P. sauteri*. Both lack sexually dimorphic characters but female *P. sufangae* differ in having the frons elevated above the clypeus (Fig. 19E) (frons as same height as clypeus in females of *P. sauteri* (Fig. 19F)).

##### Etymology.

The new species is dedicated to Mrs. Su-Fang Yu, who is a member of the Taiwan Chrysomelid Research Team (TCRT) for her contribution to the diversity of leaf beetles.

##### Distribution.

Taiwan.

### Pseudotheopea similis group

**Diagnosis.** Frontoclypeus not modified in males, elytra with short dense hair.

**Included species.***Pseudotheopea nigrita* (Medvedev), comb. nov. and *P. similis* (Kimoto), comb. nov.

#### Pseudotheopea nigrita

Taxon classificationAnimaliaColeopteraChrysomelidae

(Medvedev, 2007) comb. nov.

8E58DDCC-D2E1-5685-AB78-DC969D17928C

Figs 18D, E ; 24

Theopea nigrita Medvedev, 2007: 11 (Thailand).

##### Type.

Holotype ♀ (SMNS): “W-THAILAND, Klong / Lan NP, 50 km SW / Kamphaeng Phet, 2.-5. / VII.1997, leg. J. REJSEK // HOLOTYPUS [p] / Theopea / nigrita m. [h] / L. Medvedev det. [p] 2006 [h, w]”.

##### Other specimens examined.

**THAILAND.** Mae Hong Son: 1♂, 1♀ (JBCB), Ban Huai Po, 19°19N 97°59E, 1600-2200 m, 17.-23.V.1991, leg. L. Dembický; 3♂♂ (JBCB), Kiwlom-pass near Soppong, 19°26N 98°19E, 1400 m, 23.VI.-2.VII.2002, leg. R. and H. Fouqué.

##### Redescription.

**Figure 24.**
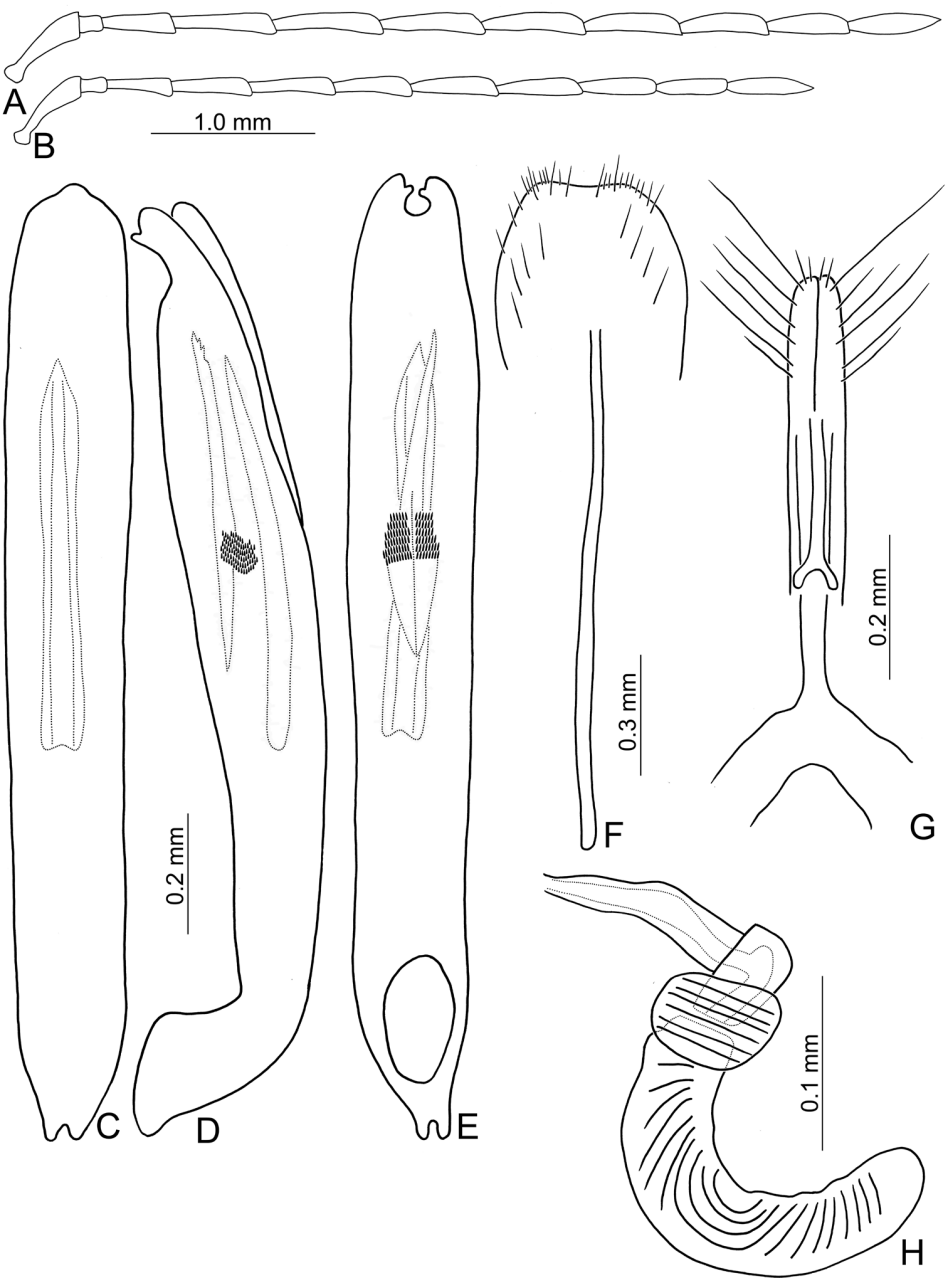
Diagnostic characters of *Pseudotheopea nigrita*. **A** Antenna, male **B** Antenna, female **C** Aedeagus, dorsal view **D** Aedeagus, lateral view **E** Aedeagus, ventral view **F** Abdominal ventrite VIII **G** Gonocoxae **H** Spermatheca.

Length 5.6–5.9 mm, width 2.3–2.5 mm. Body color (Fig. 18D, E) black, antennae and legs pale yellow, two or three apical antennomeres, and one or two apical tarsomeres darker. Antennae filiform in male, antennomeres VII-XI slightly curved (Fig. 24A), length ratios of antennomeres I–XI 1.0: 0.3: 0.8: 1.2: 1.3: 1.1: 1.2: 1.1: 1.0: 0.9: 1.0, length to width ratios of antennomeres I–XI 3.4: 1.6: 3.5: 4.9: 5.2: 4.8: 4.9: 4.8: 4.4: 4.1: 4.4; filiform and shorter in females (Fig. 24B), length ratios of antennomeres I–XI 1.0: 0.3: 0.8: 0.9: 1.0: 0.9: 1.0: 1.0: 0.9: 0.8: 1.0, length to width ratios of antennomeres I–XI 3.6: 1.8: 3.8: 4.2: 4.7: 4.3: 5.0: 4.9: 4.4: 4.3: 5.5. Elytra elongate, parallel-sided, 1.6-1.7× longer than wide; disc with dense, coarse punctures, arranged into longitudinal rows, with one indistinct longitudinal ridge between two longitudinal rows of punctures, with dense, short setae along ridges. Tarsomeres I of front legs slightly swollen in males; subparallel in females. Aedeagus (Fig. 24C–E) slender, 7.8× longer than wide; apex with shallow, broad notch, with a pair of small processes forming circular cavity; tectum short, from apex to apical 1/3, as broad as aedeagus; almost straight in lateral view, strongly curved at base, moderately curved near apex; triangular sclerites absent; internal sac with elongate, endophallic sclerite complex, 0.4× as long as aedeagus, undivided; with one ventral sclerite elongate, 0.85× as long as elongate, endophallic sclerite complex, with lateral expansion at apical 1/3, composed of dense short setae, apically tapering. Gonocoxae (Fig. 24G) elongate, both gonocoxae fused from basal 1/3 to near apex; apices convergent and narrowly rounded, each gonocoxa with eight setae along lateral margin from apex to apical 1/6; with one pair of short lateral processes at basal 2/5, recurved and combined, extending to apex. Ventrite VIII (Fig. 24F) elongate and well sclerotized; disc with several long setae at sides and near apical margin, and with dense, short setae along apical margin; spiculum extremely slender. Receptacle of spermatheca (Fig. 24H) strongly swollen; pump slender and strongly curved; proximal spermathecal duct deeply inserted into receptacle, broad and short, strongly curved near base.

##### Diagnosis.

*Pseudotheopea nigrita* (Medvedev) is easily recognized by its characteristic black color and dense setae on the elytra. In addition, a number of autapomorphic characters among genitalic structures are diagnostic, including the circular notch of the aedeagal apex; broad tectum, characteristic shape of the ventral sclerite, the recurved and combined lateral processes of the gonocoxae, and strongly curved proximal spermathecal duct of spermatheca.

##### Remarks.

Most setae are missing from the elytra of the holotype (Fig. 18E), but these setae are dense on the elytra (Fig. 18F) of other specimens examined.

##### Distribution.

Thailand.

#### Pseudotheopea similis

Taxon classificationAnimaliaColeopteraChrysomelidae

(Kimoto, 1989) comb. nov.

342D7707-0FEE-538E-897E-90664896ED15

Figs 12D–F ; 25

Theopea similis Kimoto, 1989: 201.
Theopea sauteri : Kimoto, 1989: 200 (part, misidentification).
Theopea subviridis Medvedev, 2012: 67. syn. nov.

##### Types.

*Theopea similis*. Holotype ♀ (BPBM): “LAOS. Vientiane / 31.V-3.VI.1960 [p, w] // S. Quate and / L. Quate / Collectors [p, w] // Theopea / similis / n. sp. [h, w] // HOLOTYPE [p, r]”.

*Theopea subviridis*. Holotype ♂ (LMCM, based on photographs): “S Vietnam, N. Dongnai Pr. / Nam Cat Tien Nat. Park / Exped. Russ.-Vietnamese / Tropical Centre / at light HLQ450 / leg. D. Fedorenko .X.2004 [p, w] // HOLOTYPUS / Theopea / subvidiridis / L. Medvedev [p, r]”.

##### Other specimens examined.

LAOS. 1♂ (BPBM), Umgeb. Vanky, 1963, identified as *Theopea sauteri* by Kimoto (1989).

##### Redescription.

**Figure 25.**
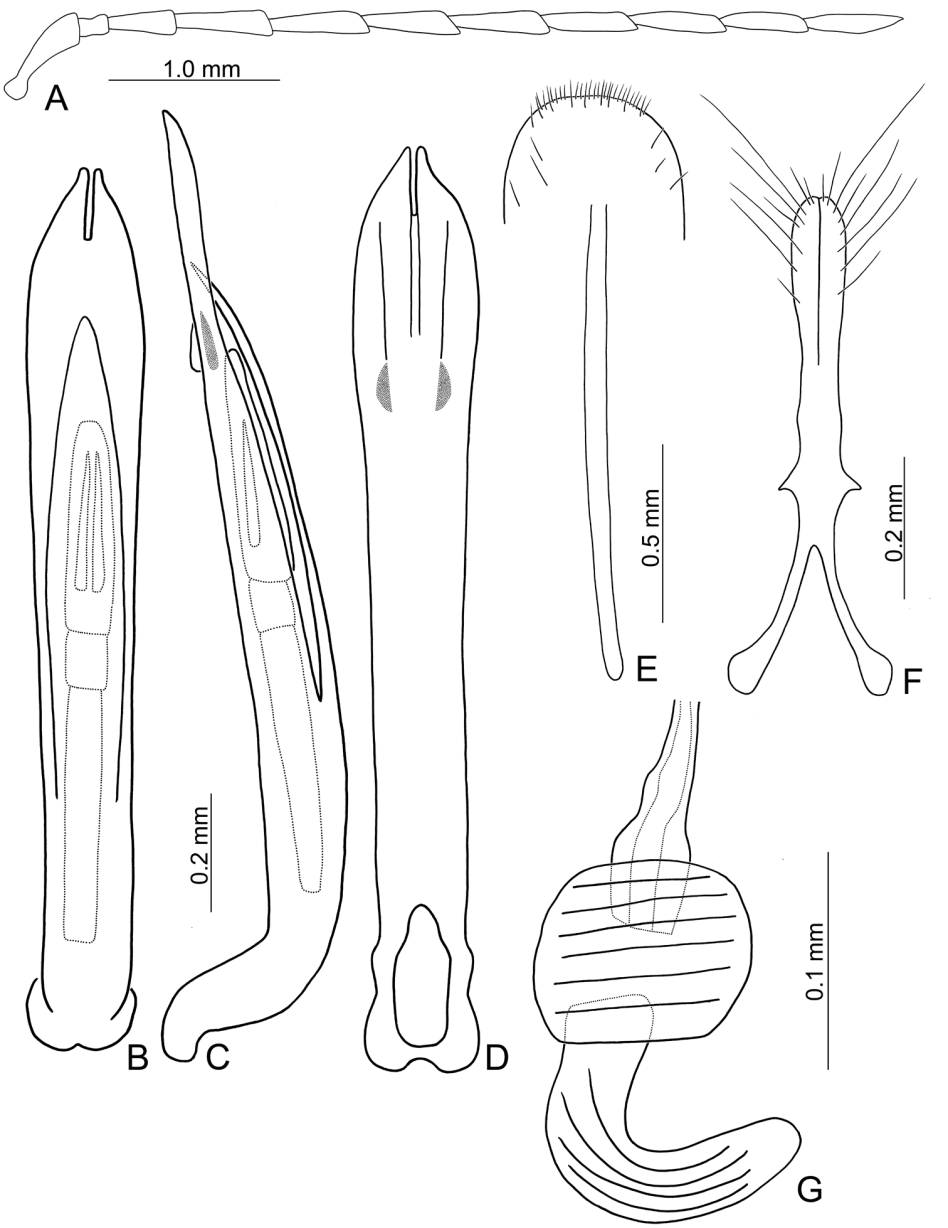
Diagnostic characters of *Pseudotheopea similis*. **A** Antenna, female **B** Aedeagus, dorsal view **C** Aedeagus, lateral view **D** Aedeagus, ventral view **E** Abdominal ventrite VIII **F** Gonocoxae **G** Spermatheca.

Length 5.0–6.4 mm, width 1.8–2.3 mm. Body color (Fig. 12D–F) metallic blue or purple, but antennae, legs, and mouth parts dark brown. Antennae filiform in females (Fig. 25A, lost in males), length ratios of antennomeres I–XI 1.0: 0.3: 0.7: 1.0: 1.0: 1.0: 1.0: 1.0: 1.0: 0.8: 1.0, length to width ratios of antennomeres I–XI 3.3: 1.6: 2.9: 4.0: 4.1: 3.9: 4.0: 4.6: 4.8: 4.4: 5.0. Elytra elongate, parallel-sided, 1.9-2.0× longer than wide; disc with dense, coarse punctures, arranged into longitudinal rows, with one indistinct longitudinal ridge between two longitudinal rows of punctures, with dense, short setae along ridges. Tarsomeres I of front legs slightly swollen in males; subparallel in females. Aedeagus (Fig. 25B–D) slender, 9.0× longer than wide; apex with shallow incision; tectum long, from apical 1/7 to basal 2/5; almost straight in lateral view, strongly curved at base; triangular sclerites small; internal sac with elongate, endophallic sclerite complex, 0.5× as long as aedeagus, composed of three sclerites, basal piece longest, 0.5× long as entire sclerite, apical piece a little shorter than basal piece, 0.4× long as entire sclerite, median piece shortest, 0.1× long as entire sclerite; with one pair of dorsal sclerites elongate, 0.5× as long as apical piece. Gonocoxae (Fig. 25F) elongate, both gonocoxae fused from basal 1/3 to apical 1/3; apices convergent and narrowly rounded, each gonocoxa with eight setae along lateral margin from apex to apical 1/6; with one pair of short lateral processes at basal 2/5. Ventrite VIII (Fig. 25E) elongate and well sclerotized; disc with several long setae at sides and near apical margin, and with dense, short setae along apical margin; spiculum extremely slender. Receptacle of spermatheca (Fig. 25G) strongly swollen; pump slender and strongly curved; proximal spermathecal duct deeply inserted into receptacle, narrow and short.

**Variation.** Reticulate microsculpture on pronotum is more or less reduced on specimens from Vietnam. A specimen from South Vietnam is metallic green in colour.

##### Diagnosis.

*Pseudotheopea similis* (Kimoto) is similar to *P. irregularis* (Takizawa) based on the indistinct ridges on the elytra and metallic blue body. This species is characterized by the presence of dense, short erect setae on the elytra. Males of *P. similis* are similar to those of *P. collaris* in the presence of paired dorsal aedeagal sclerites, but differ in the bifurcate and symmetrical apices (Fig. 25B–D) (apices obliquely truncate apex in those of *P. collaris*).

##### Remarks.

*Pseudotheopea similis* is easily recognized by the presence of dense erect setae on the elytra, which is also found on the holotype of *Theopea subviridis*. Such a characteristic feature supports their synonymy.

##### Distribution.

Laos, Vietnam.

#### Borneotheopea

Taxon classificationAnimaliaColeopteraChrysomelidae

Lee & Bezděk gen. nov.

F680F7D3-1E9C-578B-9A5B-5C2ECE1A5D65

http://zoobank.org/91CB6F48-EF54-4B12-881C-19CFADB1877E

##### Type species.

*Borneotheopea jakli* sp. nov. (here designated)

##### Redescription.

Body length 4.6–5.8 mm.

***Males.*** Head. Eyes moderately large. Anterior part of head not modified. Frontal tubercles prominent, narrow, usually produced at inner anterior angle. Penultimate maxillary palpomere not greatly swollen, apical palpomere conical. Vertex with reticulate microsculpture. Antenna 11-segmented, filiform and slender, uniform in both sexes; antennomere II very short, III long, 2.0–3.5× longer than II, 0.6–0.8× as long as I, 2.8–3.4× as long as wide. Pronotum square or transverse, 1.1–1.2× as wide as long, broadest at middle, with pair of discal depressions. Anterior pronotal border absent. Lateral margins rounded or subparallel. Disc with reticulate microsculpture.

Elytra. Surface almost glabrous (with scattered erect setae on apical part only); punctate and striate, usually with longitudinal ridges between two longitudinal rows of punctures, sometimes ridges reduced or absent in part. Epipleura gradually narrowed to apex. Disc with reticulate microsculpture.

Legs. Procoxae globular, prosternal process apically expanded, procoxal cavities closed. Protarsomere I more or less swollen. Metatibia simple, without apical spine. Length of metatarsomere I nearly equal to following tarsomeres combined. Tarsal claws appendiculate with basal tooth small and rounded. Metatarsomere I simple.

Abdomen. Last ventrite apically trilobate.

Aedeagus always ventrally flattened, apex with shallow notch. Ventral surface entirely sclerotized. Internal sac with median elongate sclerite, undivided; with single pair of large lateral sclerites.

***Females.*** Antennae slender, unmodified. Protarsomere I not modified. Posterior margin of last ventrite regularly rounded, without incisions. Spermatheca with small receptacle and C-shaped pump. Gonocoxae with split and convergent apex, apical part usually with eight long setae, base bifurcate. Ventrite VIII longitudinal, with long setae at sides and short setae along apical margin, spiculum 2.4× as long as ventrite VIII.

##### Differential diagnosis.

This new genus possesses the following characters shared with *Theopea* and *Pseudotheopea* gen. nov.: the punctures on the elytra are striate and ridges are present between two longitudinal rows of punctures; the spaces between two longitudinal rows of punctures are broader when ridges are reduced or absent. *Borneotheopea* gen. nov. is similar to *Pseudotheopea* gen. nov. based on the presence of reticulate microsculpture on the vertex and pronotum (lacking reticulate microsculpture in *Theopea*) and convergent apices of the gonocoxae in females (diverge apices in those of *Theopea*). However, *Borneotheopea* gen. nov. can be separated from other genera by the antennomeres III-X not modified in males (antennomeres III-X usually longer and curved in those of *Pseudotheopea* gen. nov.); absence of an apical spine on the metatibia (presence of apical spine on the metatibia in *Pseudotheopea* gen. nov.); swollen or modified in those of *Theopea*); broader aedeagus, < 6.0× longer than wide (> 7.0× longer than wide in *Pseudotheopea* gen. nov. and > 6.0× longer than wide in *Theopea*); the ventral surface entirely sclerotized and unmodified (with deep groove, short hollow area, hollow area in *Theopea*, or wide groove in *Pseudotheopea* gen. nov.); and with the undivided median elongate endophallic sclerite in males (divided median elongate sclerite in *Pseudotheopea* gen. nov.).

##### Etymology.

This new genus is named for its distribution combined with the genus *Theopea*.

##### Included species.

Two new species are found in Borneo: *Borneotheopea jakli* sp. nov. and *B. kalimantanensis* sp. nov.

#### Borneotheopea jakli sp. nov.

Taxon classificationAnimaliaColeopteraChrysomelidae

22765A0B-2397-5FD7-82A6-7789C828B638

http://zoobank.org/5A45681E-5605-4790-8732-5BDA28138BB0

Figs 26A–C ; 27

##### Types.

Holotype ♂ (NMPC), **INDONESIA**. South Kalimantan: Kandagan distr., 17 km NE of Laksado vill., 900 m, 3-22.IX.1997, leg. S. Jákl. Paratypes. 16♂♂, 2♀♀ (JBCB), same data as holotype.

##### Description.

**Figure 26.**
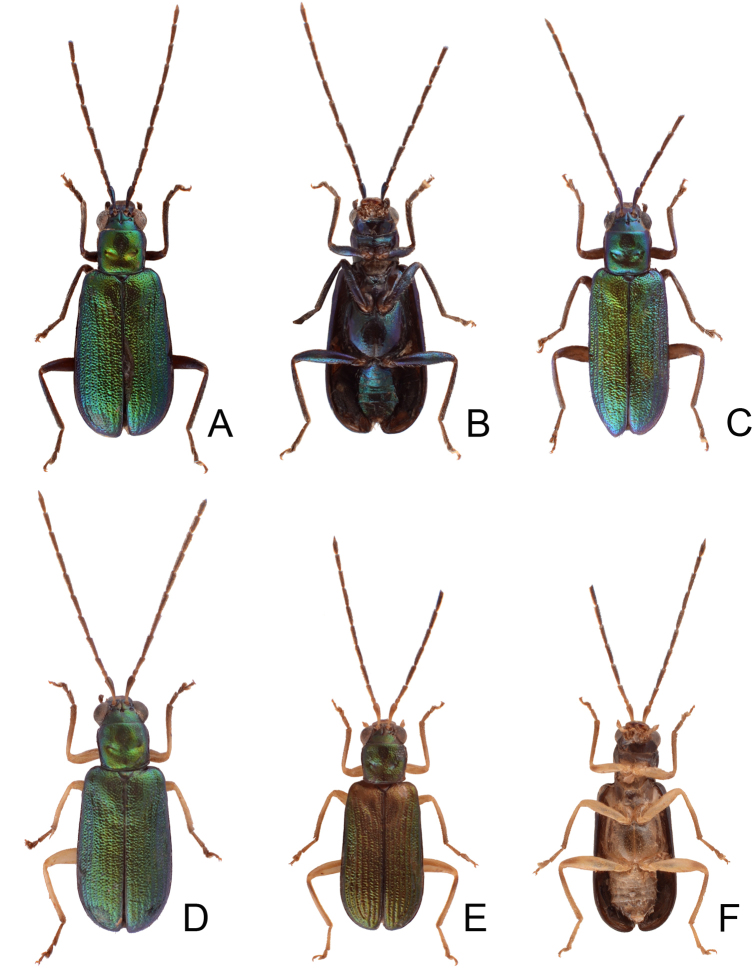
Habitus of *Borneotheopea jakli* sp. nov. and *B. kalimantanensis* sp. nov. **A***B. jakli* sp. nov., male, dorsal view **B** Same, ventral view **C***B. jakli* sp. nov., female, dorsal view **D***B. jakli* sp. nov., male, color variation, dorsal view **E***B. kalimantanensis* sp. nov., male, dorsal view **F** Same, ventral view.

**Figure 27.**
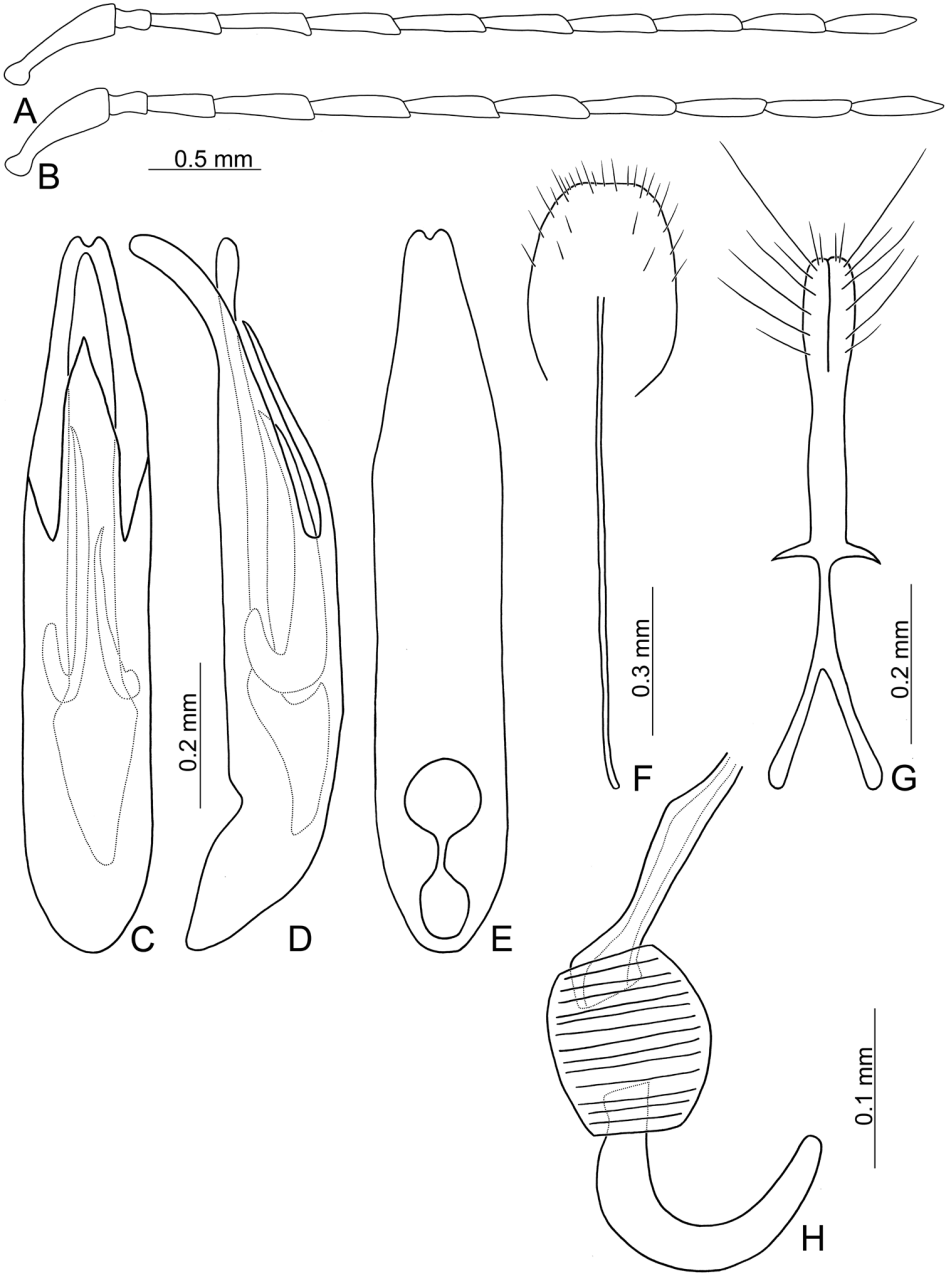
Diagnostic characters of *Borneotheopea jakli* sp. nov. **A** Antenna, male **B** Antenna, female **C** Aedeagus, dorsal view **D** Aedeagus, lateral view **E** Aedeagus, ventral view **F** Abdominal ventrite VIII **G** Gonocoxae **H** Spermatheca.

Length 4.6–5.0 mm, width 1.7–1.8 mm. Body color (Fig. 26A–C) metallic blue or green; ventral part, mouth parts, and antennae dark brown to black. Antennae filiform in males (Fig. 27A), length ratios of antennomeres I–XI 1.0: 0.3: 0.6: 0.7: 0.7: 0.7: 0.7: 0.7: 0.7: 0.6: 0.7, length to width ratios of antennomeres I–XI 4.0: 1.9: 3.4: 4.5: 4.6: 4.3: 4.7: 4.6: 4.8: 4.6: 4.5; similar in females (Fig. 27B), length ratios of antennomeres I–XI 1.0: 0.3: 0.6: 0.9: 0.9: 0.8: 0.8: 0.8: 0.8: 0.7: 0.8, length to width ratios of antennomeres I–XI 3.2: 1.8: 2.8: 4.2: 4.5: 4.3: 4.3: 4.6: 5.0: 5.1: 5.2. Elytra elongate, parallel-sided, 1.8-2.0× longer than wide; disc with dense, coarse punctures, arranged into longitudinal rows, with one indistinct longitudinal ridge between two longitudinal rows of punctures, with dense, short setae along ridges. Tarsomeres I of front legs slightly swollen in males. Aedeagus (Fig. 27C–E) slender, 5.5× longer than wide, parallel from apical 1/3 to near base, narrowed towards apex, apical margin medially depressed; tectum short, from near apex to middle; almost straight in lateral view, apically curved; ventral surface entirely sclerotized, triangular sclerites absent; internal sac with elongate endophallic sclerite, 0.8× as long as aedeagus, one pair of lateral sclerites elongate and hook-like, strongly recurved basally, left sclerite much longer than right sclerite. Gonocoxae (Fig. 27G) elongate, both gonocoxae fused from basal 1/3 to apical 1/3; apices convergent and narrowly rounded, each gonocoxa with eight setae along lateral margin from apex to apical 1/6; with one pair of short lateral processes at basal 2/5. Ventrite VIII (Fig. 27F) elongate and well sclerotized; disc with several long setae at sides and near apical margin, and with dense, short setae along apical margin; spiculum extremely slender. Receptacle of spermatheca (Fig. 27H) strongly swollen; pump slender and strongly curved; proximal spermathecal duct deeply inserted into receptacle, elongate and narrow.

##### Diagnosis.

**Figure 28.**
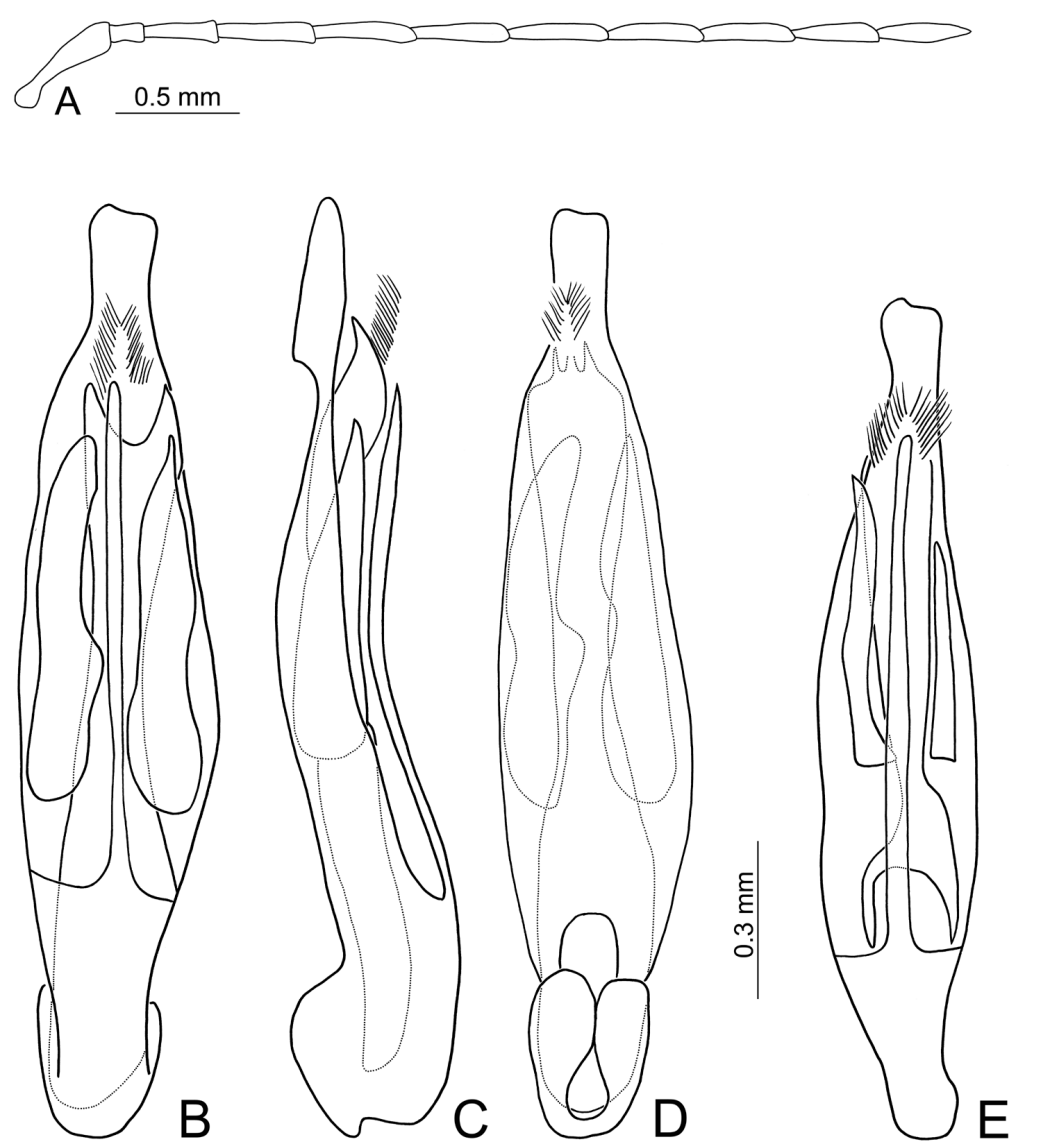
Diagnostic characters of *Borneotheopea kalimantanensis* sp. nov. **A** Antenna, male **B** Aedeagus, dorsal view **C** Aedeagus, lateral view **D** Aedeagus, ventral view **E** Aedeagus, dorsal view, from Sabah.

*Borneotheopea jakli* sp. nov. is easily distinguished from the other member of the genus, *B. kalimantanensis* sp. nov., based on the indistinct ridges on the elytra and metallic blue ventral surface (Fig. 26A–D) (distinct ridges on the elytra and yellowish brown ventral surface in *B. kalimantanensis* sp. nov. (Fig. 26E, F)). Males of *B. jakli* sp. nov. are also easily separated from those of *B. kalimantanensis* sp. nov. by the aedeagal apex directed ventrally and lacking angular processes (Fig. 27D) (the apex directed anteriorly and with angular process at apical 1/6 of aedeagus in *B. kalimantanensis* sp. nov. (Fig. 28C)), short, broad tectum (Fig. 27C) (extremely slender tectum in *B. kalimantanensis* sp. nov. (Fig. 28B)), absence of setae at apex of median elongate sclerite (Fig. 27C–E) (presence of clustered setae at apex of median elongate sclerite in *B. kalimantanensis* sp. nov. (Fig. 28B, C)).

##### Etymology.

The new species is dedicated to the Czech specialist Stanislav Jákl who collected the type specimens.

##### Distribution.

Indonesia: South Kalimantan.

#### Borneotheopea kalimantanensis sp. nov.

Taxon classificationAnimaliaColeopteraChrysomelidae

15C58A8F-6F92-59CA-A3F9-F4CE77CBE4E0

http://zoobank.org/C492882F-D6C3-4939-8F11-DE2C27F16A9F

Figs 26D–F ; 28

##### Types.

Holotype ♂ (NMPC), **INDONESIA**. South Kalimantan: Kandagan distr., 17 km NE of Laksado vill., 900 m, 3-22.IX.1997, leg. S. Jákl. Paratypes. 11♂♂ (JBCB), same data as holotype; **MALAYSIA**. Sabah: 2♂♂ (TARI), Trusmadi, 1.X.2014, leg. Y.-T. Wang; 2♂♂ (TARI), same but with “2.X.2014”.

##### Description.

Length 5.1–5.8 mm, width 1.8–2.3 mm. Body color (Fig. 26D–F) yellowish brown, head and pronotum metallic green, prosternite dark brown, elytra yellowish but laterally and apically metallic green, antenna black except three basal antennomeres paler. Antennae filiform in males (Fig. 28A), length ratios of antennomeres I–XI 1.0: 0.3: 0.8: 0.9: 0.8: 0.8: 0.8: 0.8: 0.8: 0.7: 0.8, length to width ratios of antennomeres I–XI 3.9: 1.8: 3.4: 4.4: 5.2: 5.3: 5.9: 5.8: 5.6: 4.6: 4.6. Elytra elongate, parallel-sided, 1.9-2.0× longer than wide; disc with dense, coarse punctures, arranged into longitudinal rows, with one indistinct longitudinal ridge between two longitudinal rows of punctures, with dense, short setae along ridges. Tarsomeres I of front legs slightly swollen in males. Aedeagus (Fig. 28B–D) slender, 4.7× longer than wide; widest at basal 2/5, narrowed towards apical 1/7 and basal 1/5, parallel from apical 1/7 to apex, apical margin truncate, slightly depressed medially; tectum extremely slender, from apical 1/5 to basal 1/4; recurved in lateral view, angular at apical 1/6, straight from apical 1/6 to apex; ventral surface entirely sclerotized, triangular sclerites absent; internal sac with elongate endophallic sclerite, 0.9× as long as aedeagus, apex with one pair of small processes directed ventrally, one pair of longitudinal rows of hair-like setae basally connected with apex of endophallic sclerite, one pair of lateral sclerites large and hook-like, subequal in length but with different apical shapes; basal opening medially closed.

Female unknown.

**Variation.** Specimens from Sabah have slender lateral sclerites of the internal sac (Fig. 28E).

##### Diagnosis.

*Borneotheopea kalimantanensis* sp. nov. is easily distinguished from the other member of the genus, *B. jakli* sp. nov., based on the distinct ridges on the elytra and yellowish brown ventral surface (Fig. 26E, F) (indistinct ridges on the elytra and metallic blue ventral surface in *B. jakli* sp. nov. (Fig. 26A–D). Males of B. *kalimantanensis* sp. nov. are also easily separated from those of *B. jakli* sp. nov. by the anterior directed apex and angular process at apical 1/6 of the aedeagus (Fig. 28C) (the apex of aedeagus directed ventrally and without angular processes in *B. jakli* sp. nov. (Fig. 27D)), extremely slender tectum (Fig. 28B) (short and wide tectum in *B. jakli* sp. nov. (Fig. 27C)), presence of clustered setae at apex of median elongate sclerite (Fig. 28B, C) (without setae at apex of median elongate sclerite in *B. jakli* sp. nov. (Fig. 27C–E)).

##### Etymology.

The new species is named for its type locality.

##### Distribution.

Indonesia: South Kalimantan; Malaysia: Sabah.

### Key to the species of *Pseudotheopea* gen. nov. and *Borneotheopea* gen. nov.

**Table:** 

1	Some antennomeres curved or apically broadened in male; metatibia with apical spine; aedeagus with median elongate sclerite divided, with small lateral sclerites present or absent, triangular sclerites present	**(*Pseudotheopea* gen. nov.) 2**
–	Antennomeres filiform, uniform in both sexes; metatibia without apical spines; aedeagus with median elongate sclerites undivided, with large lateral sclerites, triangular sclerites absent	**(*Borneotheopea* gen. nov.) 25**
2	Frontoclypeus modified in males, with concavity between eyes	**(*P. costata* group) 3**
–	Frontoclypeus not modified in males	**13**
3	Concavity between eyes in males semi-circular, with one central erect process and one pair of baso-lateral processes (Fig. 13A, B), Philippines	**4**
–	Concavity between eyes in males transverse, anterior margin narrowly rounded, with one central erect process in some species but without baso-lateral processes, Southeast Asia except Philippines	**5**
4	Large species, 7.0-7.2 mm long; reddish brown dorsum; without lateral process at apex of antennomere I in males	***P. costata* (Allard)**
–	Small species, 5.0-5.7 mm long; metallic blue dorsum; with lateral process at apex of antennomere I in males (Fig. 14A)	***P. gressitti* sp. nov.**
5	General body color reddish brown, but elytra metallic blue (Fig. 5A–C)	***P. aeneipennis* (Gressitt & Kimoto)**
–	Body metallic green, blue, or purple	**6**
6	Body color golden green (Figs 8A–F; 15A–F)	**7**
–	Body color metallic blue or purple	**10**
7	Ridges on elytra distinct and convex (Fig. 8D, 8F); concavity between eyes anteriorly narrowed (Fig. 9C, D), Vietnam	***P. clypealis* (Medvedev)**
–	Ridges on elytra indistinct (Figs 8A, C; 15A, B, D–E); concavity between eyes transverse (Figs 9A, B; 16A–D)	**8**
8	Presence of convex area surrounding scutellum in males (Fig. 8A); concavity between eyes wide, but without erect processes (Fig. 9A, B), India	***P. boreri* sp. nov.**
–	Lacking convex area surrounding scutellum in males (Fig. 15A, D); concavity between eyes wide and with one erect process (Fig. 16C, D) or narrow (Fig. 16A, B)	**9**
9	Concavity between eyes wide and with one erect process (Fig. 16C, D), China	***P. smaragdina* (Gressitt & Kimoto)**
–	Concavity between eyes narrow (Fig. 16A, B), Laos	***P. hsingtzungi* sp. nov.**
10	Ridges on elytra distinct and convex (Fig. 15A, C, F, H)	**11**
–	Ridges on elytra indistinct (Fig. 5D, F, H)	**12**
11	All ridges on elytra distinct and convex	***P. kimotoi* sp. nov.**
–	Distinct convex ridges intertwined with indistinct ridges	***P. leehsuehae* sp. nov.**
12	Concavity between eyes with one pair of erect processes	***P. azurea* (Gressitt & Kimoto)**
–	Concavity between eyes with median longitudinal ridge	***P. sufangae* sp. nov.**
13	Longitudinal ridges on elytra distinct	**14**
–	Longitudinal ridges on elytra indistinct or reduced	**21**
14	Elytra with extremely coarse punctures, space between punctures narrower than diameters of punctures; head and prothorax yellow except vertex and pronotum; Taiwan	***P. collaris* (Kimoto)**
–	Elytra with coarse punctures, space between punctures broader than diameters of punctures; head and prothorax metallic blue or green except mouth parts	**15**
15	Body color metallic green; longitudinal ridges on elytra apically abbreviated from apical 1/3; antennomeres III-VII straight in males; Taiwan	***P. cheni* (Lee & Bezděk)**
–	Body color metallic blue; longitudinal ridges on elytra not apically abbreviated; antennomeres III-VII more or less curved	**(*P. sauteri* species group) 16**
16	Males with longitudinal ridges on the elytra more or less reduced	**17**
–	Males with longitudinal ridges on the elytra prominent	**20**
17	Aedeagus asymmetrical, curved to the right	**18**
–	Aedeagus symmetrical	**19**
18	Aedeagus relatively slender, 10.0× longer than wide; dorsal sclerite of endophallus extremely elongate, 3.6× longer than basal piece; Laos	***P. sekerkai* (Lee & Bezděk)**
–	Aedeagus relatively broad, 9.0× longer than wide; dorsal sclerite of endophallus less elongate, 1.6× longer than basal piece; China	***P. coerulea* (Gressitt & Kimoto)**
19	Triangular sclerites of endophallus elongate; ventral sclerites absent; basal piece longer than apical piece, with longitudinal row of tiny teeth along lateral margin; India	***P. geiseri* (Lee & Bezděk)**
–	Triangular sclerites of endophallus small; ventral sclerites present; basal piece shorter than apical piece, without tiny teeth; China, Laos, Vietnam	***P. laosensis* (Lee & Bezděk)**
20	Males with antennomeres III–X moderately curved; swollen tarsomeres I of front legs not apically narrowed; Taiwan	***P. sauteri* (Chûjô)**
–	Males with antennomeres III–X straight; swollen tarsomeres I of front legs apically narrowed; China	***P. hainanensis* (Lee & Bezděk)**
21	Elytra with dense, short, erect setae	**(*P. similis* group) 24**
–	Elytra with sparse, short, erect setae	**22**
22	Elytra with extremely coarse punctures, space between punctures narrower than diameters of punctures; Japan	***P. aureoviridis* (Chûjô)**
–	Elytra with moderately coarse punctures, space between punctures broader than diameters of punctures	**23**
23	Body color sexually dimorphic, elytra yellowish brown with metallic green sides, pronotum and vertex metallic green in males; elytra entirely metallic green, prothorax and head yellow in females; hypomeron yellowish brown; antenna in males relatively shorter, antennomeres V–IX less than six times longer than wide; Taiwan	***P. kanmiyai* (Kimoto)**
–	Body color not sexually dimorphic, elytra, pronotum, and vertex metallic green or blue in both sexes; hypomeron dark or blackish brown; antenna in males more slender, antennomeres V-IX more than six times longer than wide; Taiwan	***P. irregularis* (Takizawa)**
24	Body color black (Fig. 18E, F)	***P. nigrita* (Medvedev)**
–	Body color metallic green (Fig. 12D–F)	***P. similis* (Kimoto)**
25	Ridges on elytra distinct (Fig. 26E); ventral surface yellowish brown (Fig. 26F)	***B. kalimantanensis* sp. nov.**
–	Ridges on elytra indistinct (Figs 26A, C, D); ventral surface metallic green (Fig. 26B)	***B. jakli* sp. nov.**

## Supplementary Material

XML Treatment for Theopea bicolor

XML Treatment for Theopea bicoloroides

XML Treatment for Theopea mouhoti

XML Treatment for Pseudotheopea

XML Treatment for Pseudotheopea aeneipennis

XML Treatment for Pseudotheopea azurea

XML Treatment for Pseudotheopea boreri

XML Treatment for Pseudotheopea clypealis

XML Treatment for Pseudotheopea gressitti

XML Treatment for Pseudotheopea hsingtzungi

XML Treatment for Pseudotheopea kimotoi

XML Treatment for Pseudotheopea leehsuehae

XML Treatment for Pseudotheopea smaragdina

XML Treatment for Pseudotheopea sufangae

XML Treatment for Pseudotheopea nigrita

XML Treatment for Pseudotheopea similis

XML Treatment for Borneotheopea

XML Treatment for Borneotheopea jakli

XML Treatment for Borneotheopea kalimantanensis
